# Supplementary material for: Non-canonical H3K79me2-dependent pathways promote the survival of MLL-rearranged leukemia
Source: eLife. 2021 Jul 15;10:e64960. doi: 10.7554/eLife.64960 (PMC8315800; doi:10.7554/eLife.64960)
Supplement: Source data 1. [file elife-64960-data1.zip › source data folder 1/Figure 1 source data 11 1C blot labels.pdf]

Figure 1C

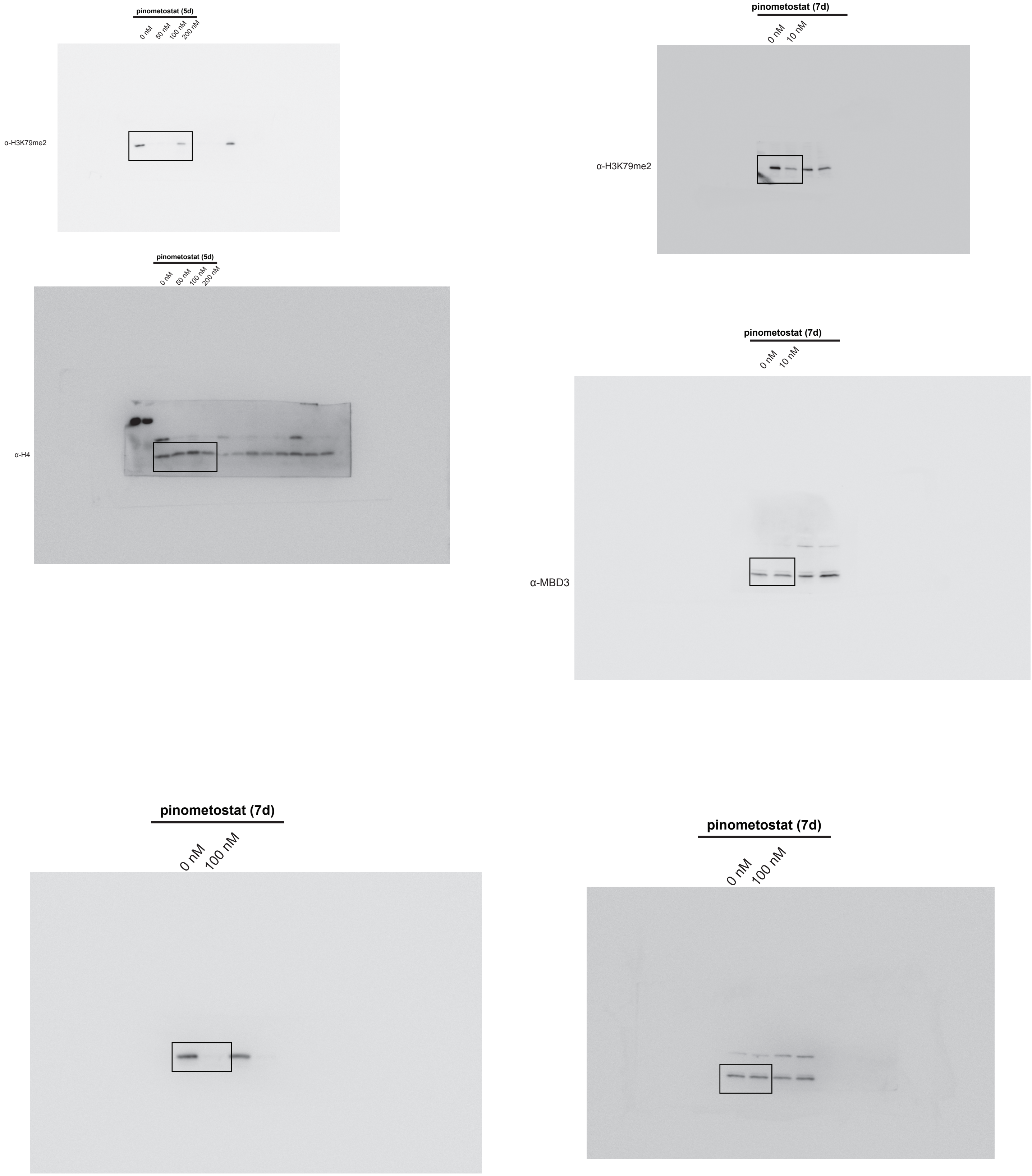
pinometostat (5d)
0 nM
50 nM
100 nM
200 nM
α-H3K79me2
pinometostat (7d)
0 nM
10 nM
α-H3K79me2
pinometostat (5d)
0 nM
50 nM
100 nM
200 nM
α-H4
pinometostat (7d)
0 nM
10 nM
α-MBD3
pinometostat (7d)
0 nM
100 nM
pinometostat (7d)
0 nM
100 nM
