# Supplementary figures and images for: Non-canonical H3K79me2-dependent pathways promote the survival of MLL-rearranged leukemia
Source: eLife. 2021 Jul 15;10:e64960. doi: 10.7554/eLife.64960 (PMC8315800; doi:10.7554/eLife.64960)

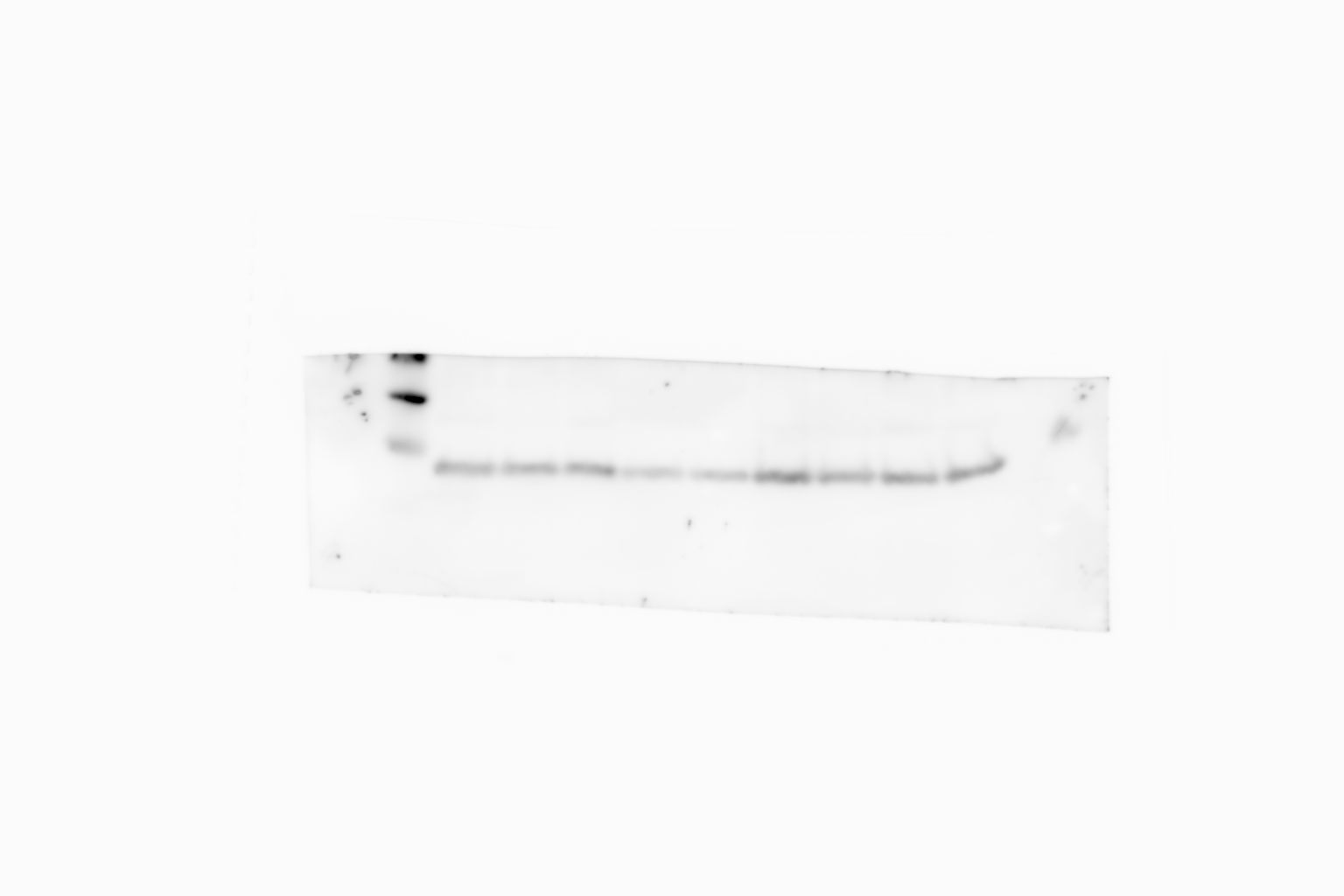

Supplement: Source data 1. [file elife-64960-data1.zip › source data folder 1/Figure 1 source data 10 1E H4 bottom.tif]

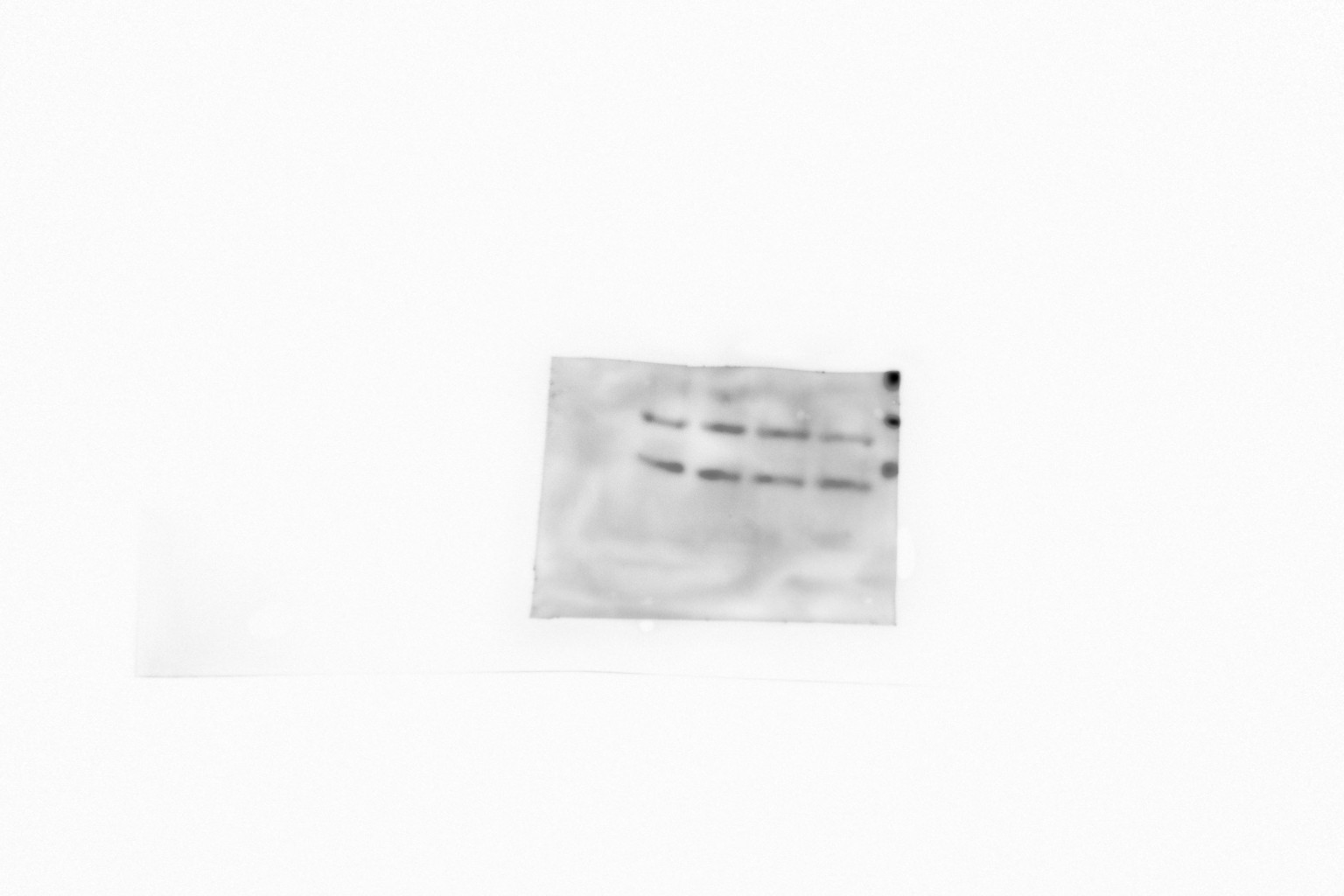

Supplement: Source data 1. [file elife-64960-data1.zip › source data folder 1/Figure 1 source data 7 1E H4 top.tif]

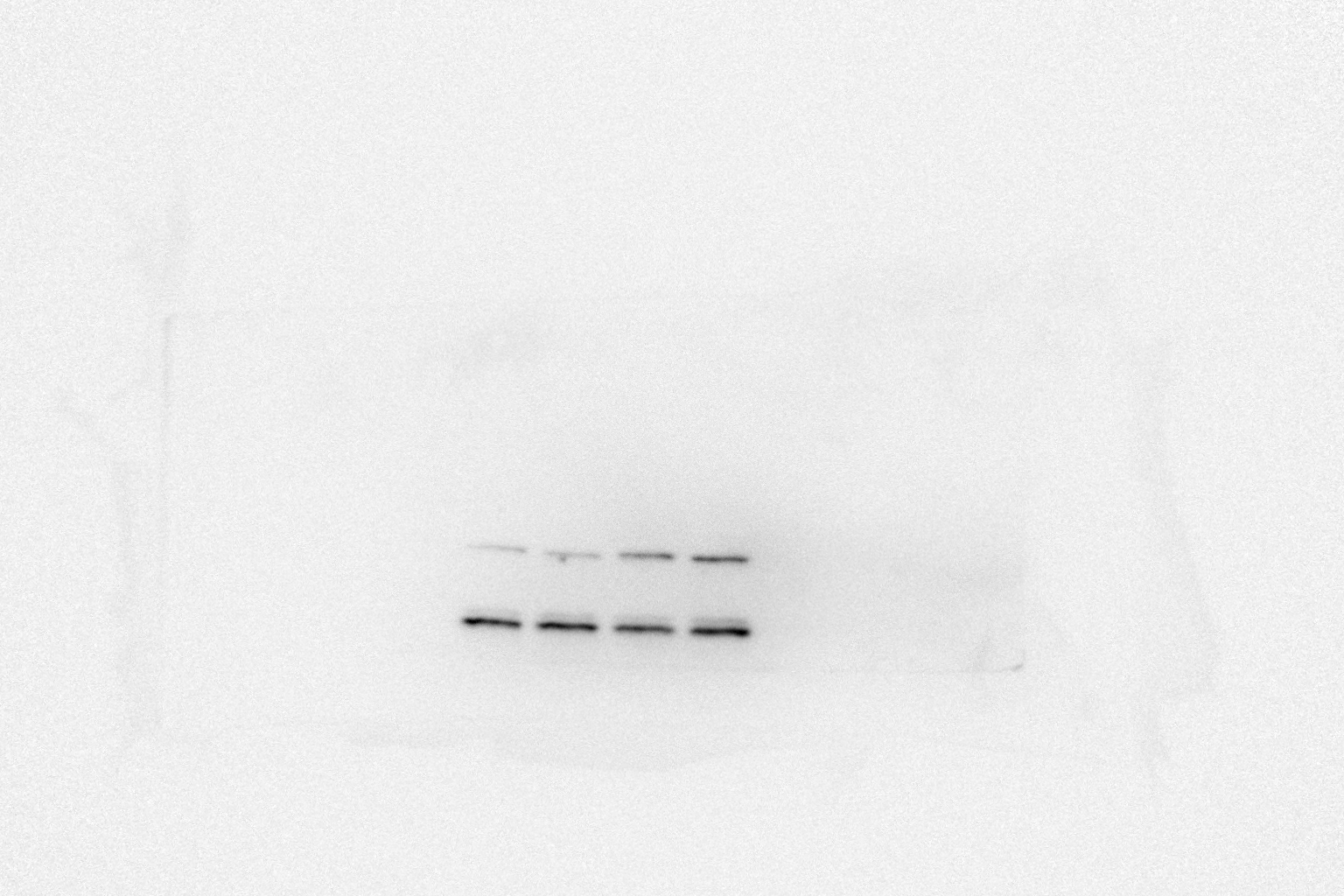

Supplement: Source data 1. [file elife-64960-data1.zip › source data folder 1/Figure 1 source data 6 1C MBD3 100 nM.tif]

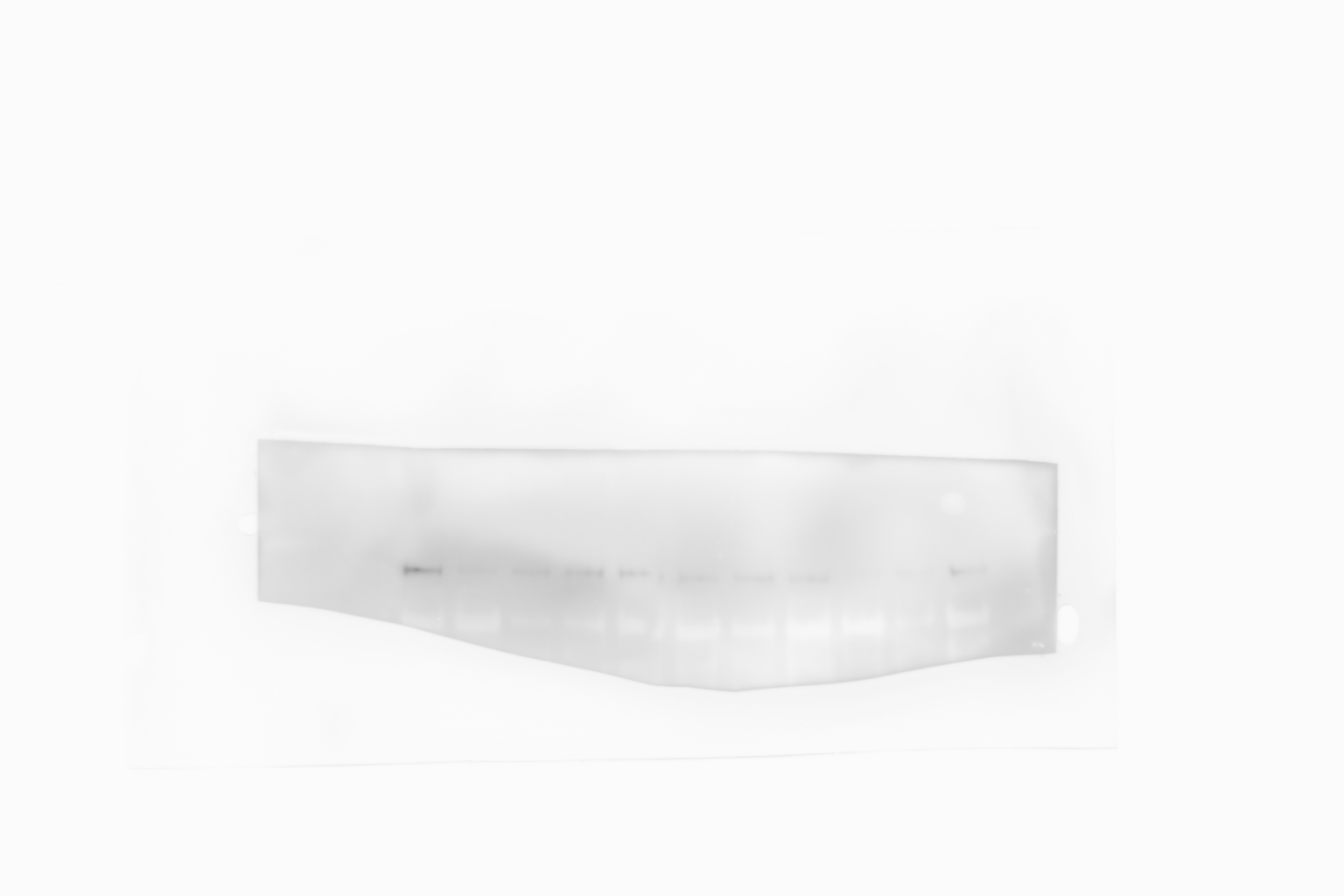

Supplement: Source data 1. [file elife-64960-data1.zip › source data folder 1/Figure 4 figure supplement 1 source data 3 S4B p-STAT5.tif]

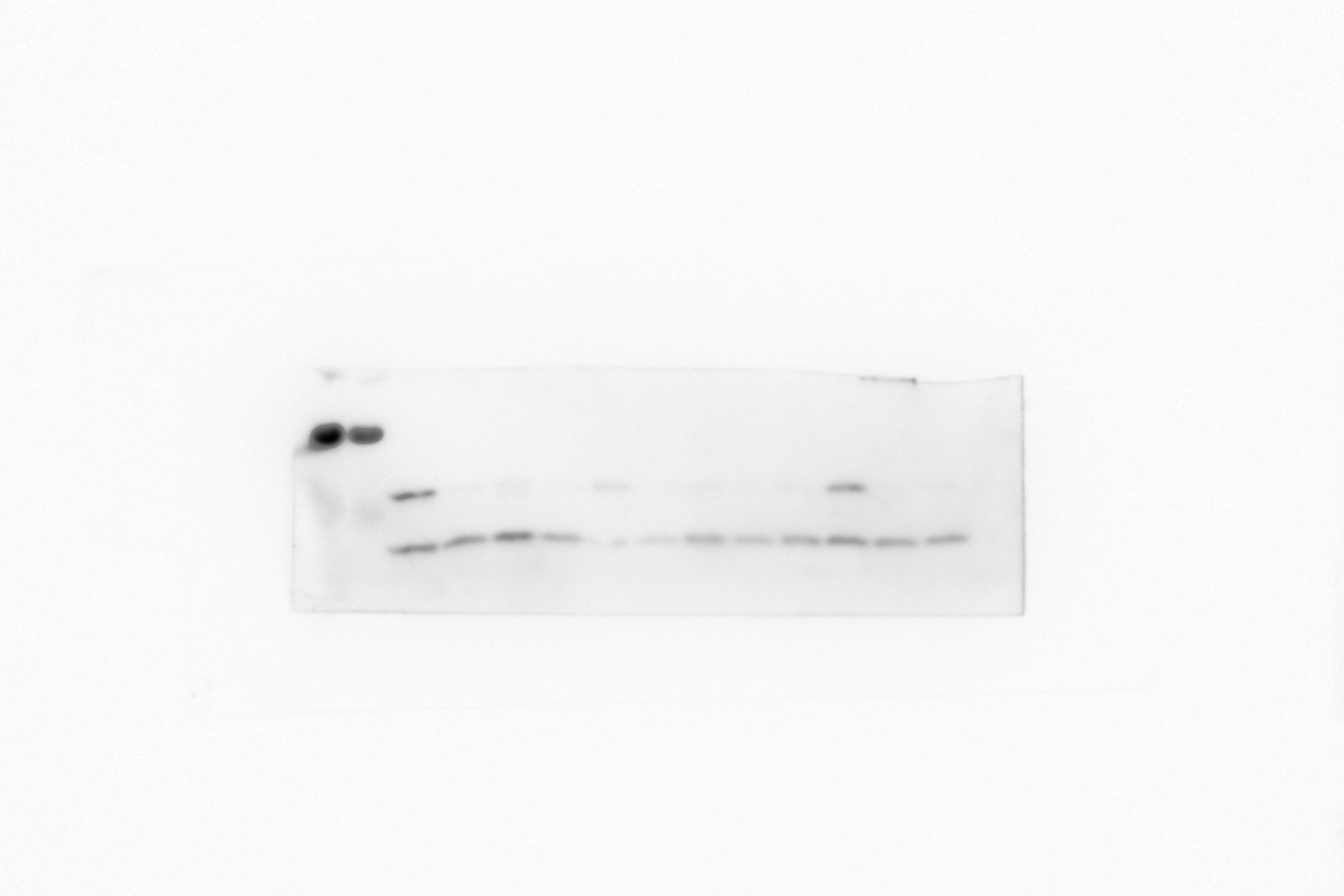

Supplement: Source data 1. [file elife-64960-data1.zip › source data folder 1/Figure 1 source data 4 1C H4 top.tif]

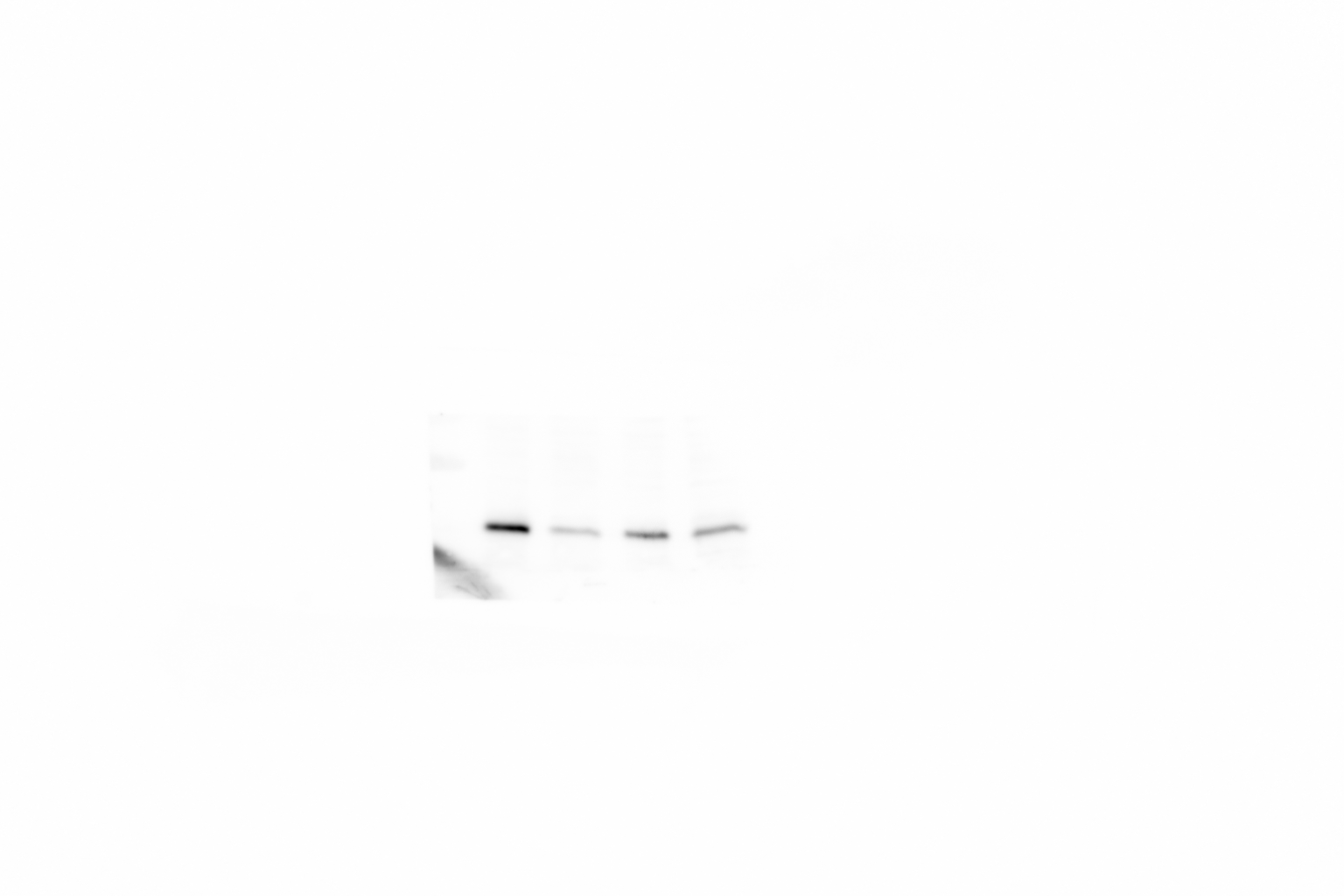

Supplement: Source data 1. [file elife-64960-data1.zip › source data folder 1/Figure 1 source data 1 1C H3K79me2 10 nM.tif]

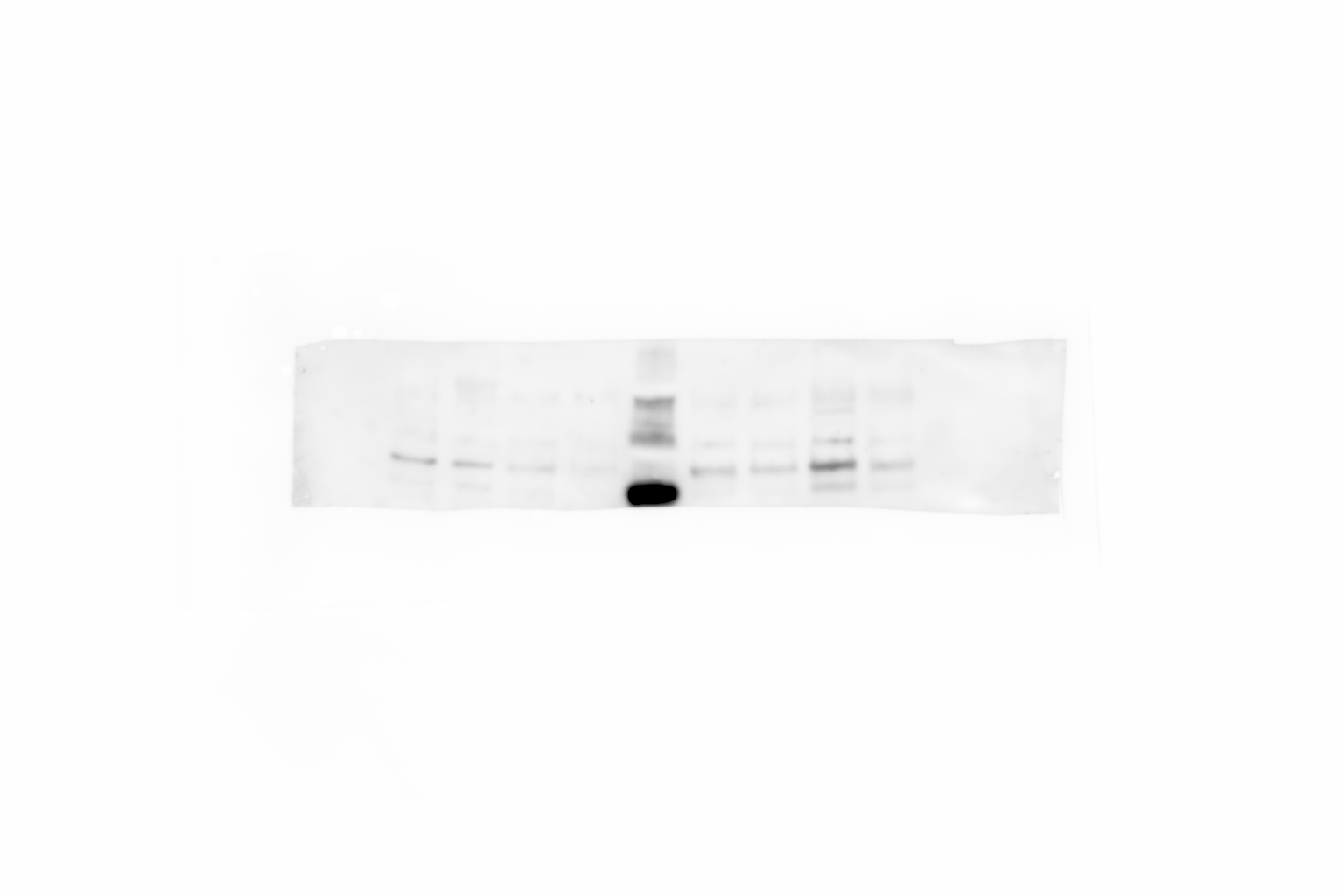

Supplement: Source data 1. [file elife-64960-data1.zip › source data folder 1/Figure 1 source data 8 1E HOXA9.tif]

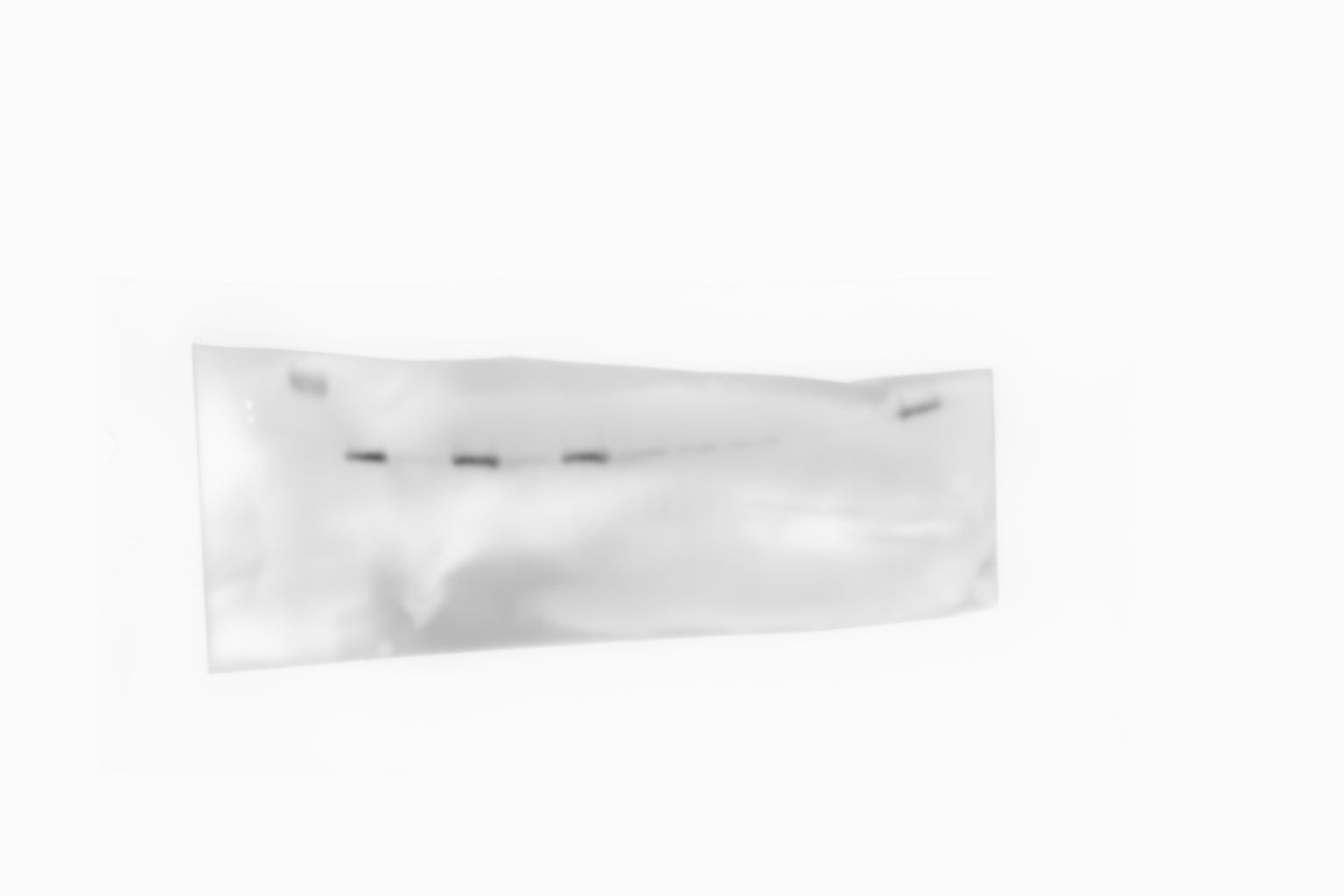

Supplement: Source data 1. [file elife-64960-data1.zip › source data folder 1/Figure 4 figure supplement 1 source data 2 S4B H3K79me2.tif]

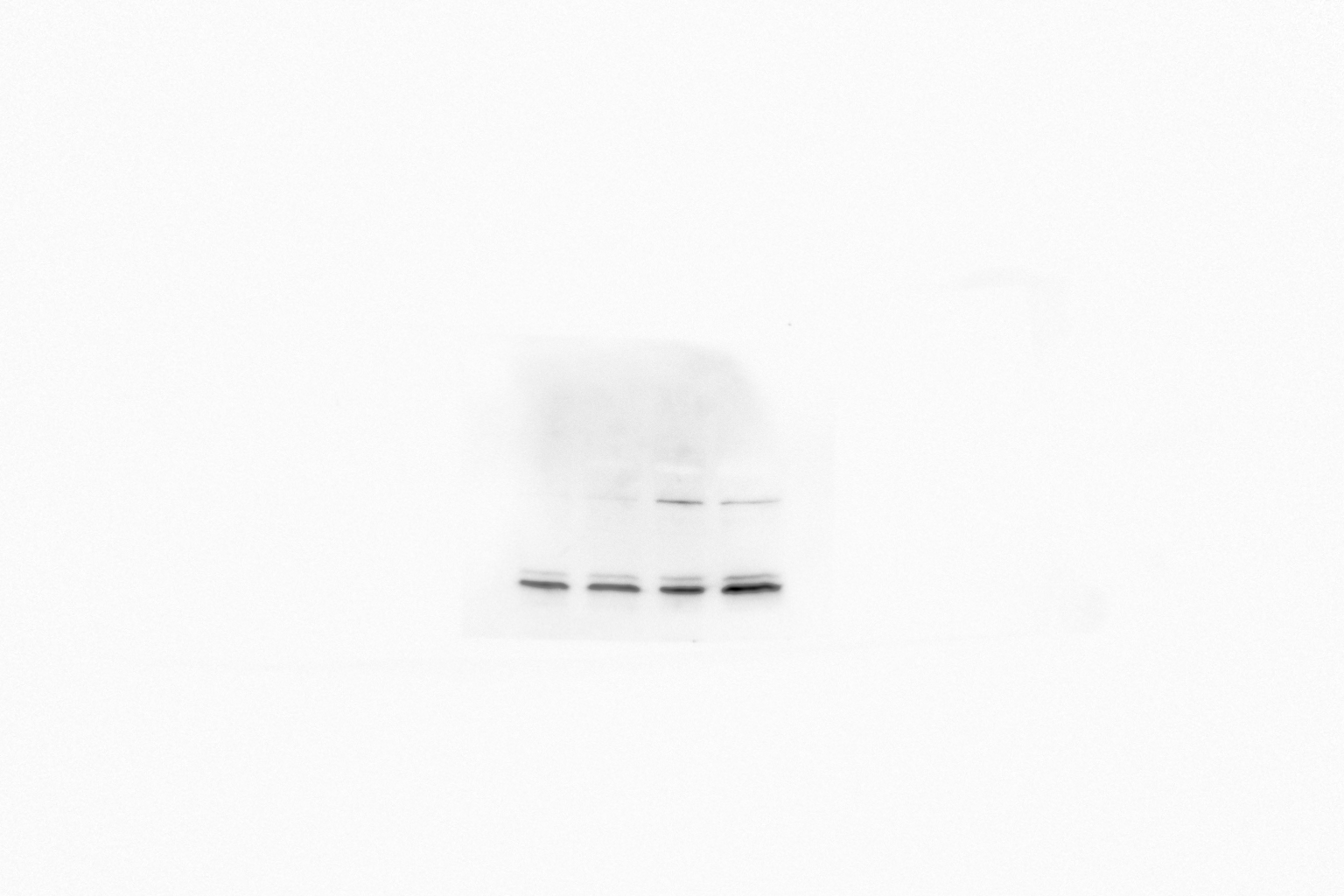

Supplement: Source data 1. [file elife-64960-data1.zip › source data folder 1/Figure 1 source data 5 1C MBD3 10 nM.tif]

Figure 2H

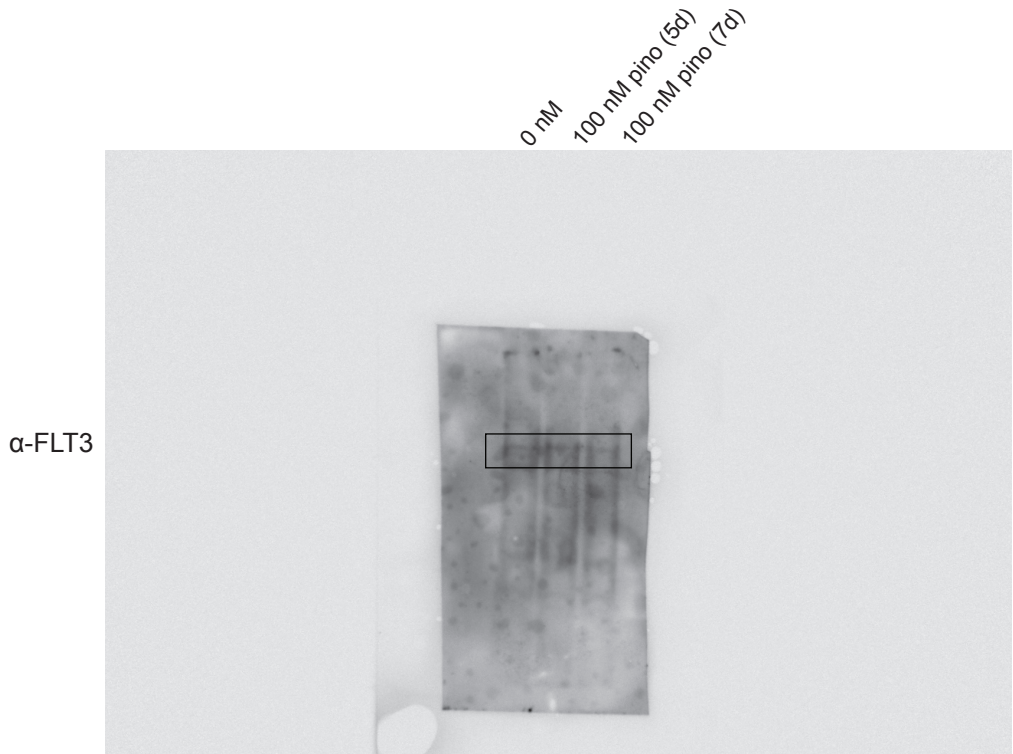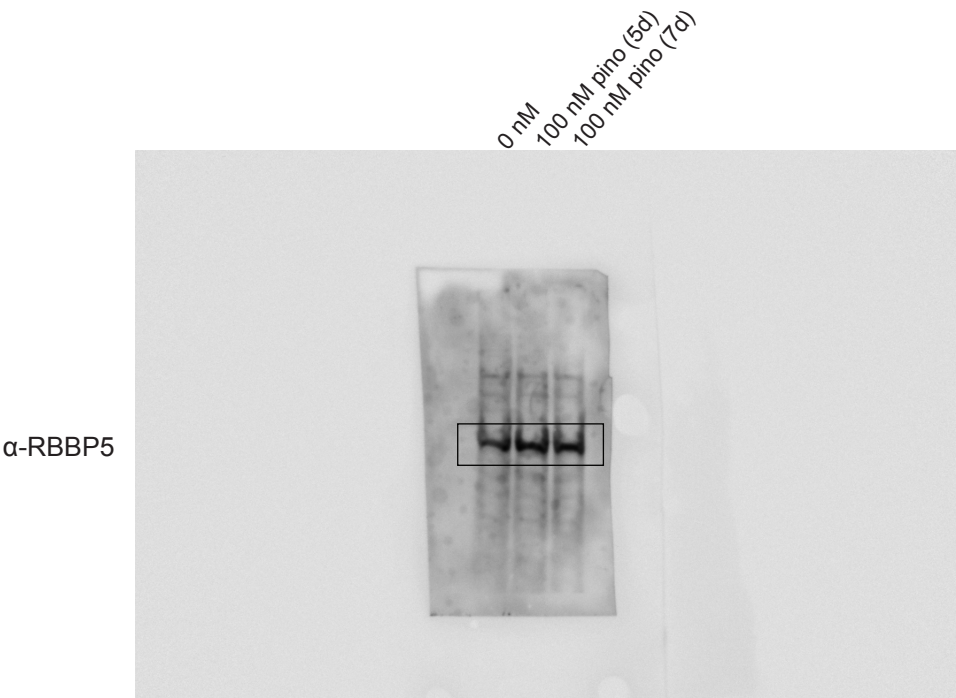

Supplement: Source data 1. [file elife-64960-data1.zip › source data folder 1/Figure 2 source data 3 2H blot labels.pdf]

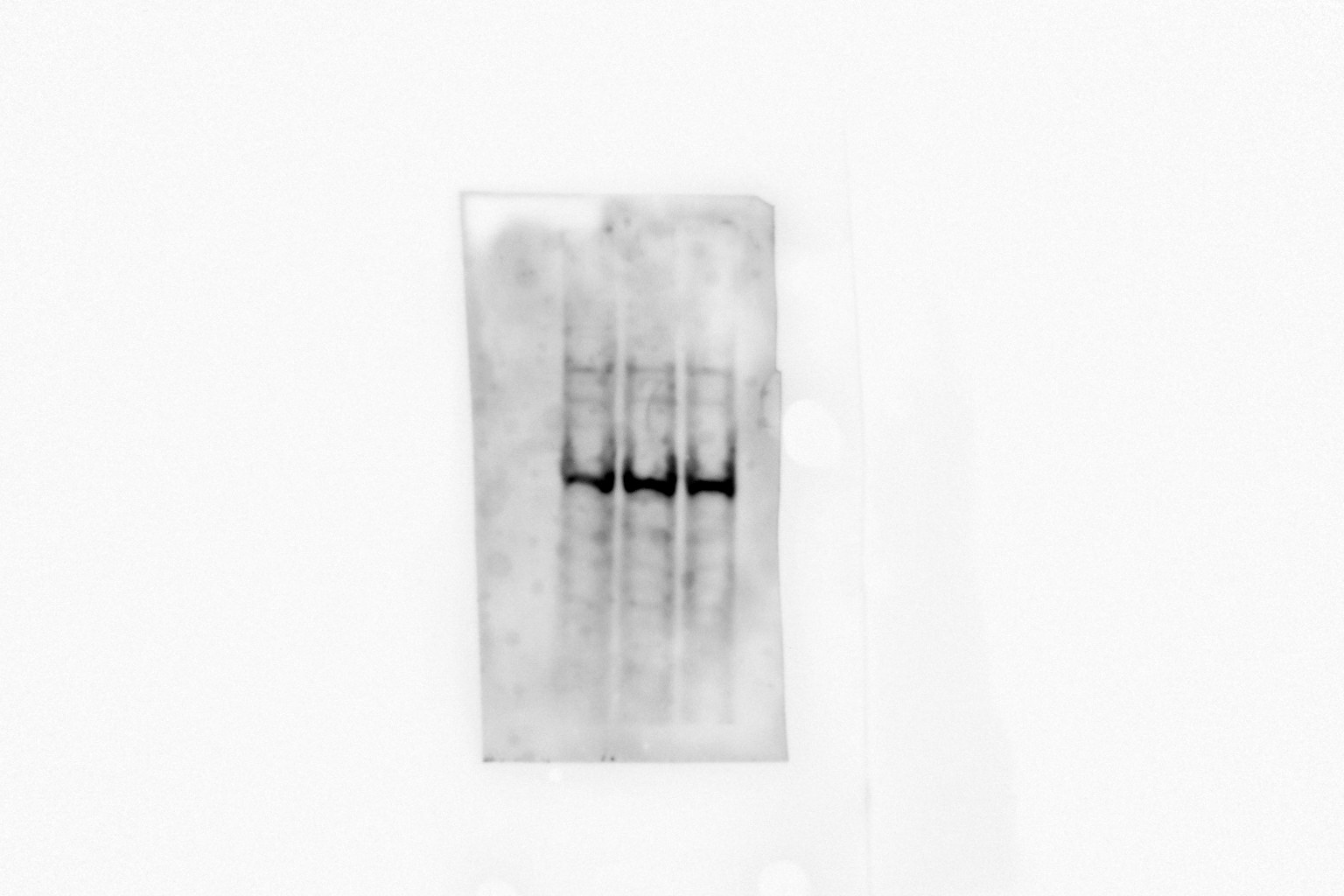

Supplement: Source data 1. [file elife-64960-data1.zip › source data folder 1/Figure 2 source data 2 2H RBBP5.tif]

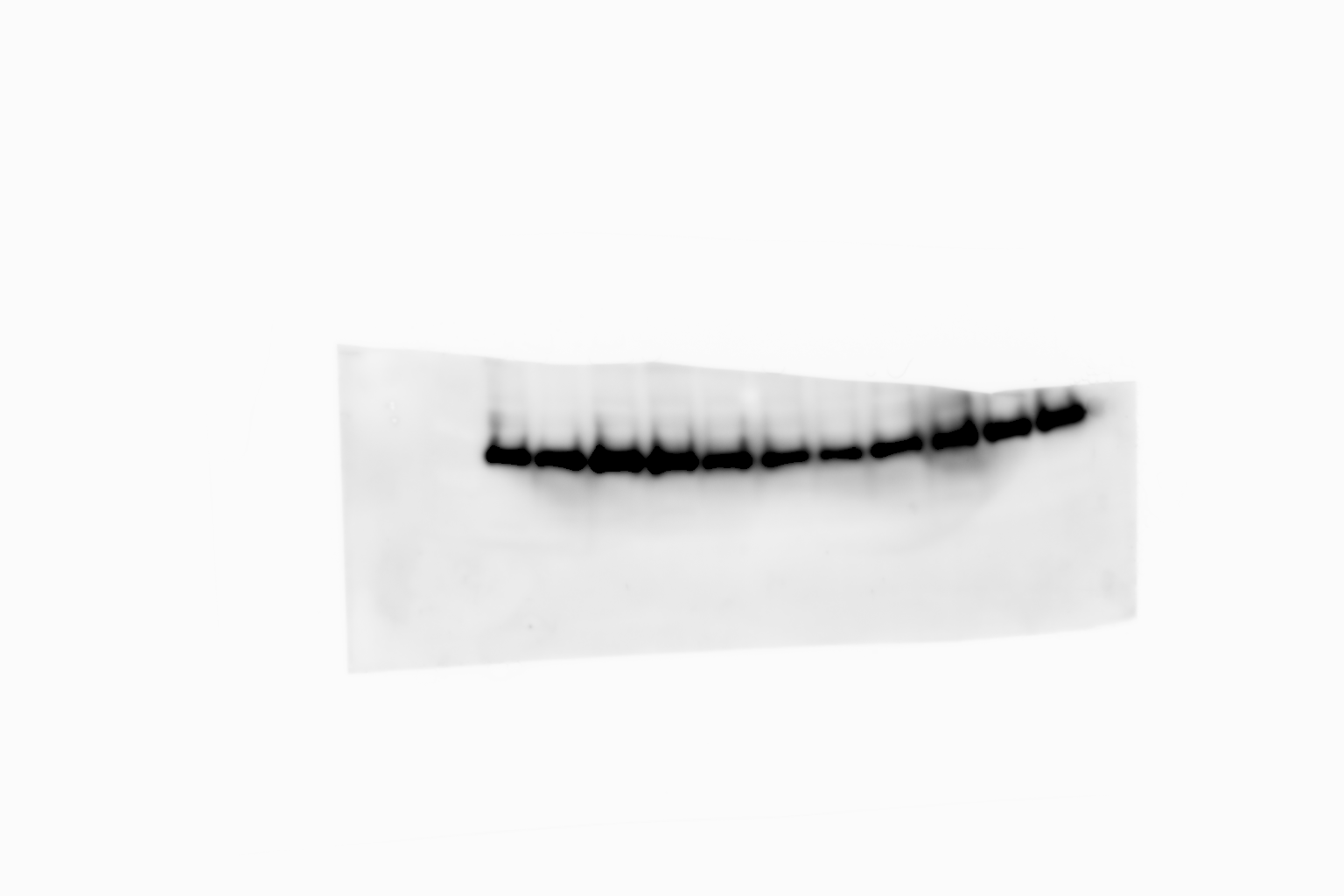

Supplement: Source data 1. [file elife-64960-data1.zip › source data folder 1/Figure 4 figure supplement 1 source data 1 S4B H2B.tif]

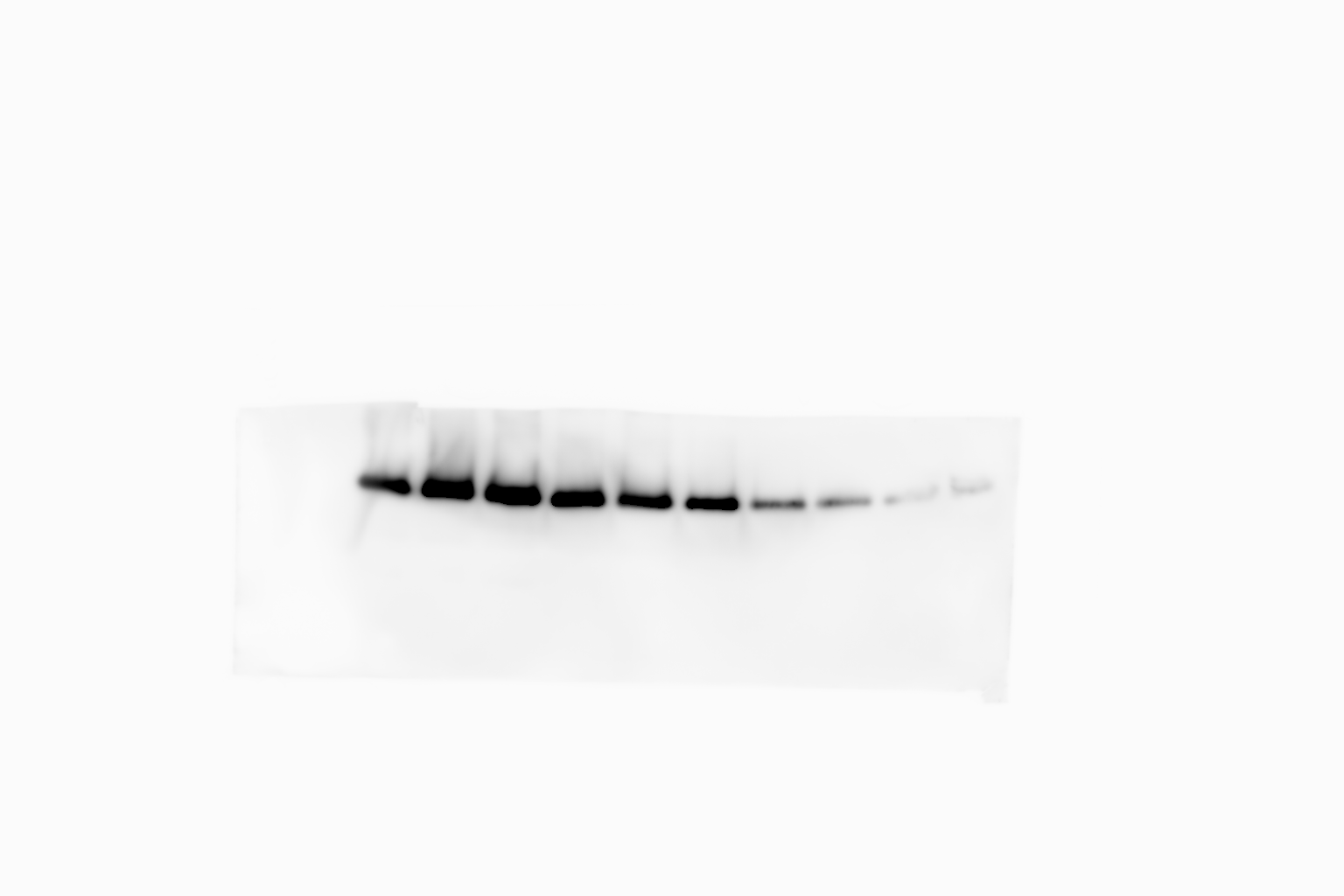

Supplement: Source data 1. [file elife-64960-data1.zip › source data folder 1/Figure 4 figure supplement 1 source data 4 S4C H3.tif]

Figure 1E

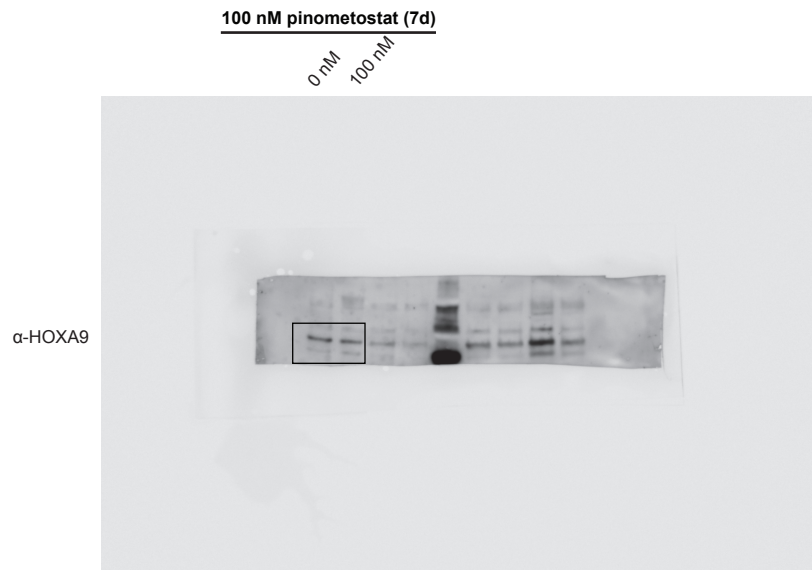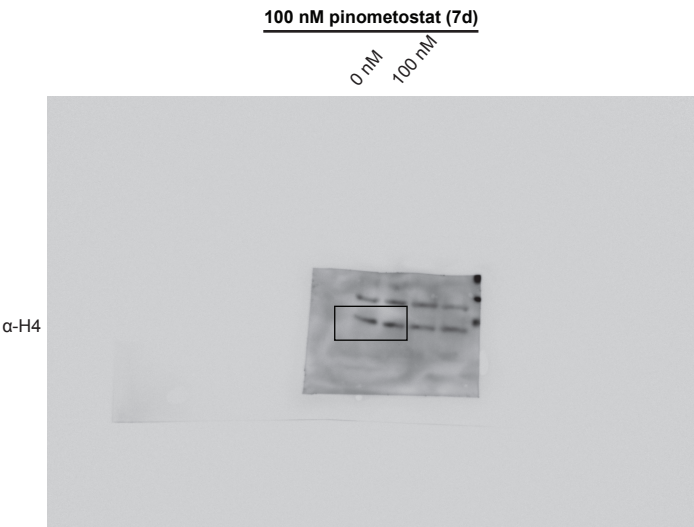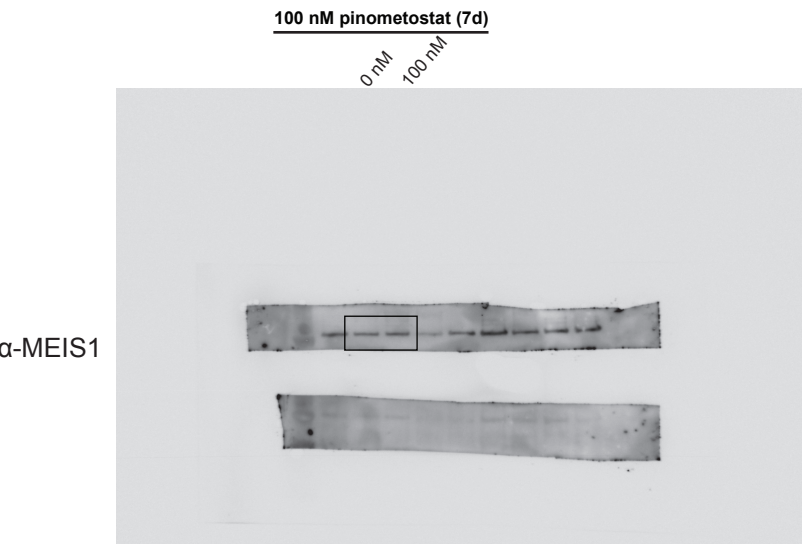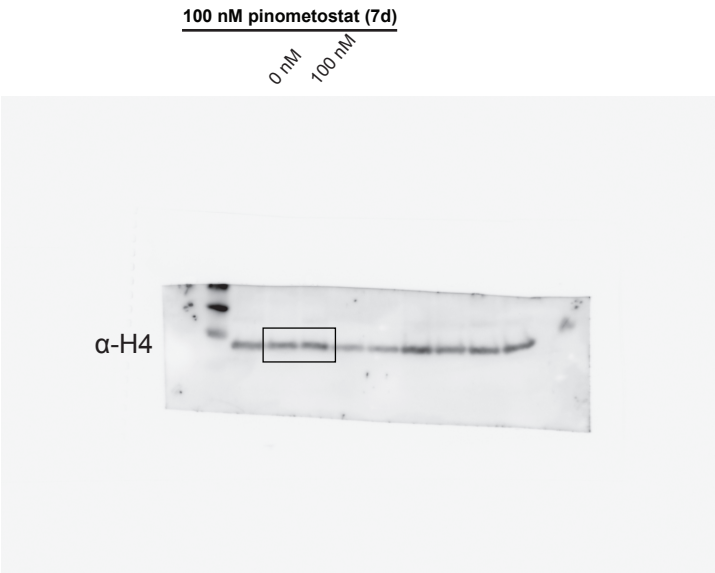

Supplement: Source data 1. [file elife-64960-data1.zip › source data folder 1/Figure 1 source data 12 1E blot labels.pdf]

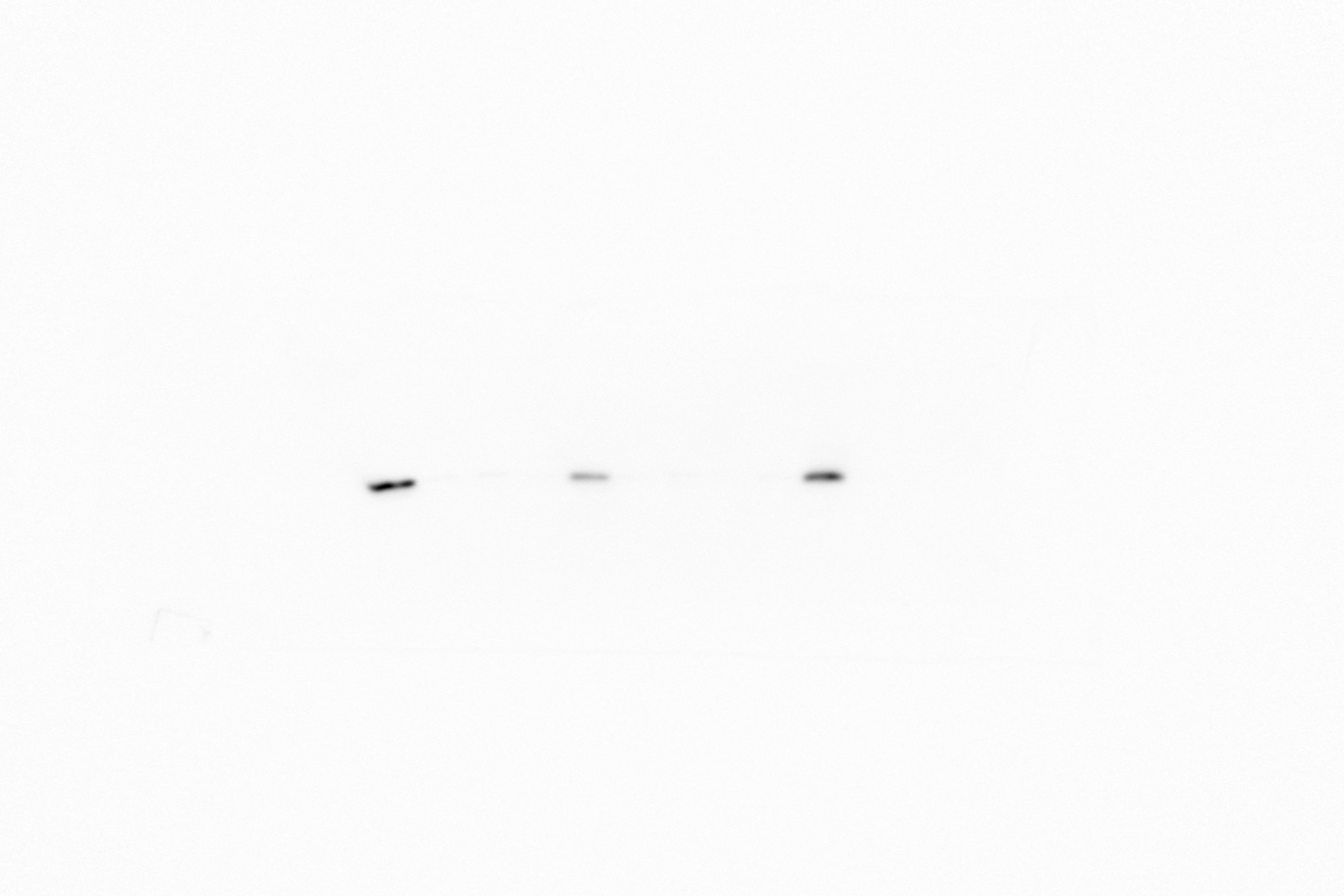

Supplement: Source data 1. [file elife-64960-data1.zip › source data folder 1/Figure 1 source data 3 1C H3K79me2 top.tif]

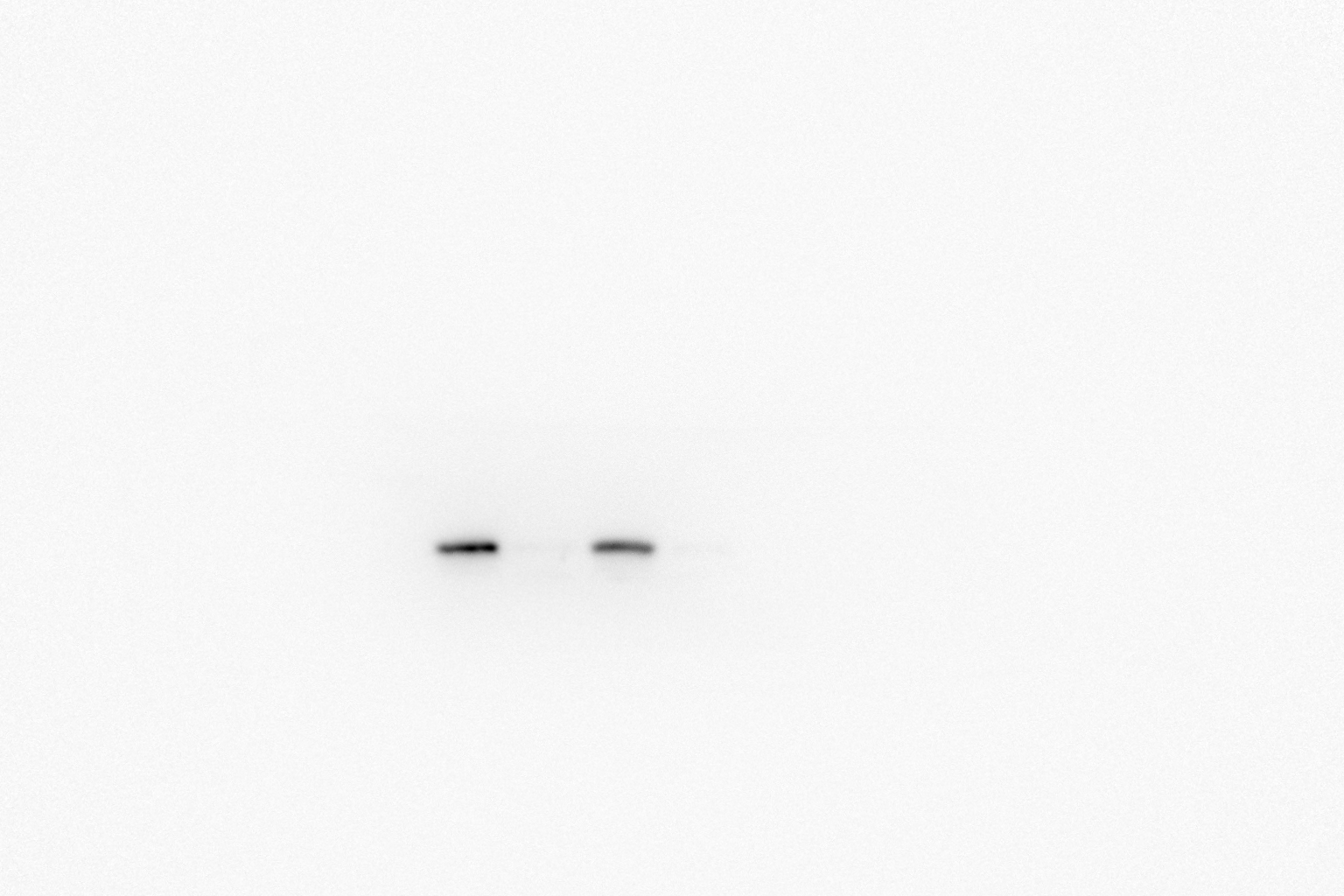

Supplement: Source data 1. [file elife-64960-data1.zip › source data folder 1/Figure 1 source data 2 1C H3K79me2 100 nM.tif]

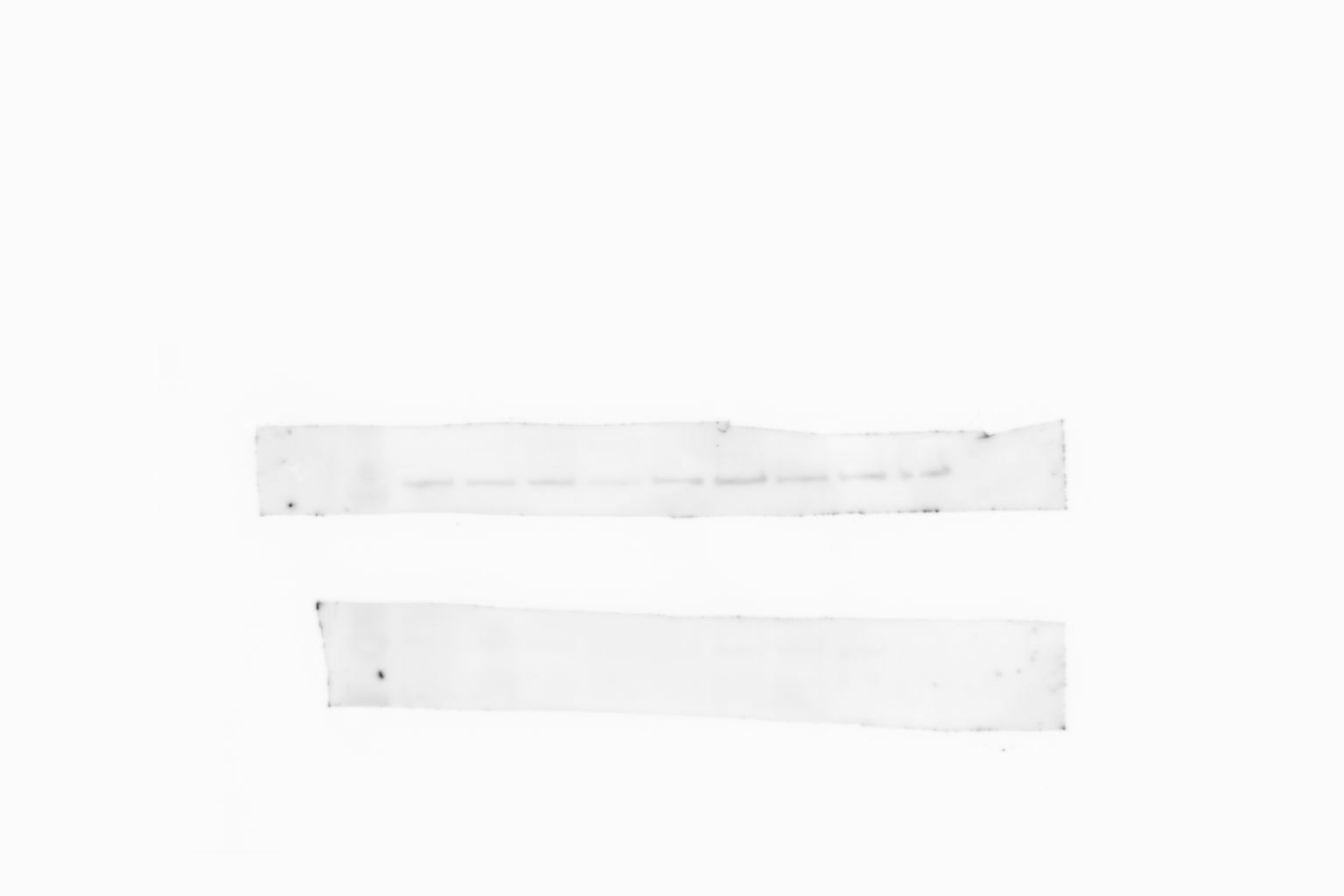

Supplement: Source data 1. [file elife-64960-data1.zip › source data folder 1/Figure 1 source data 9 1E MEIS1.tif]

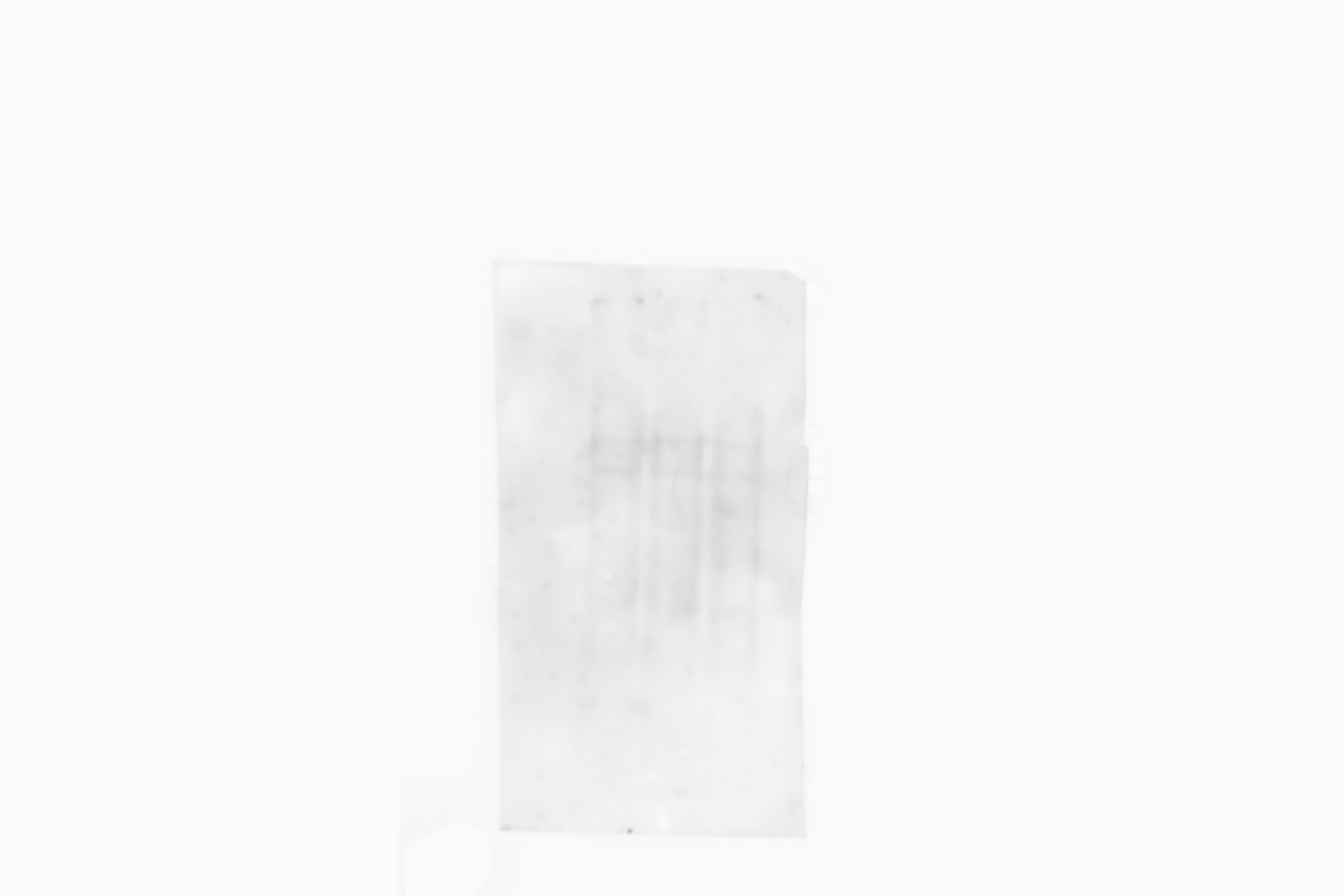

Supplement: Source data 1. [file elife-64960-data1.zip › source data folder 1/Figure 2 source data 1 2H FLT3.tif]

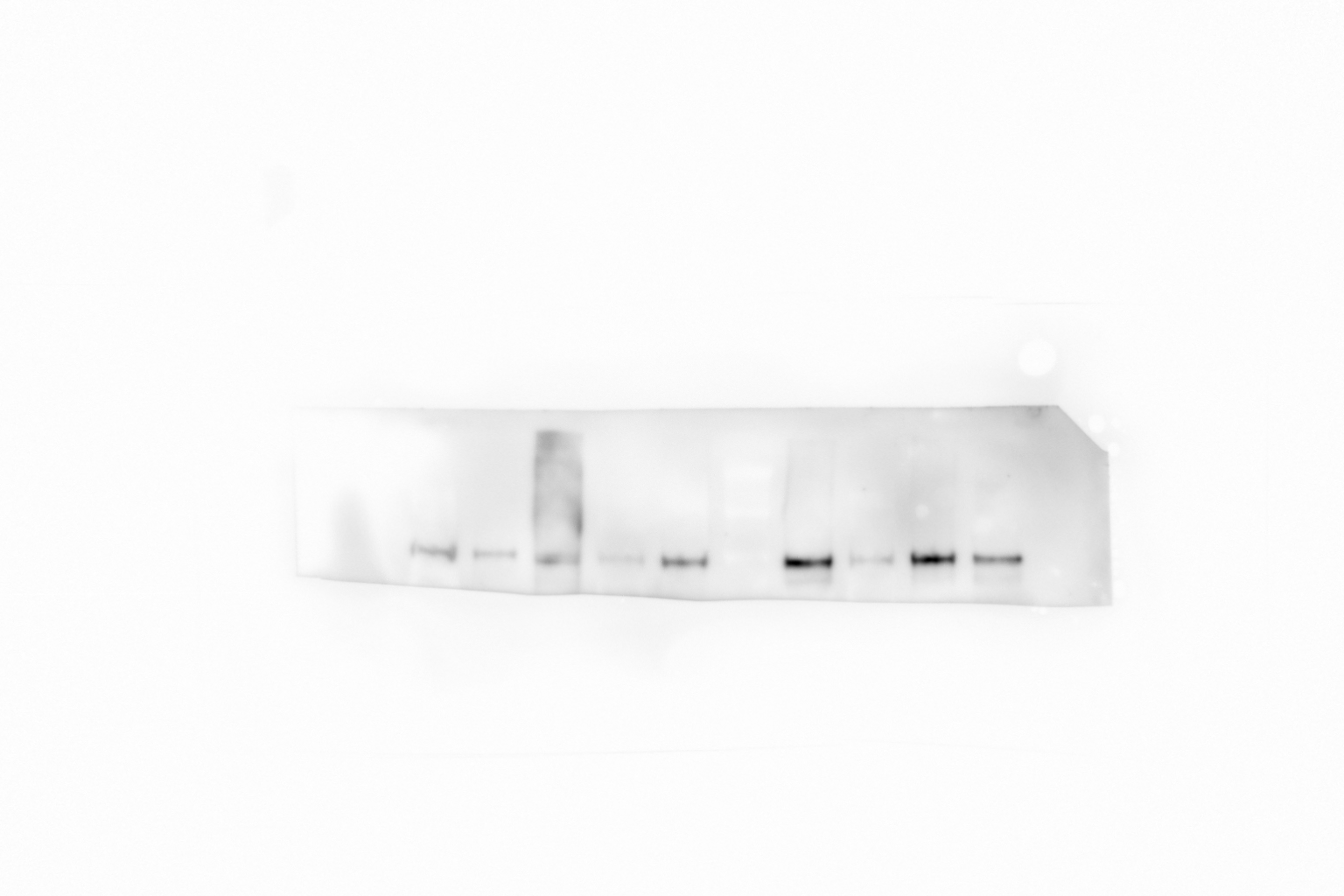

Supplement: Source data 2. [file elife-64960-data2.zip › source data folder 2/Figure 4 source data 7 4B p-STAT5 MV4;11.tif]

Figure 4B (right)

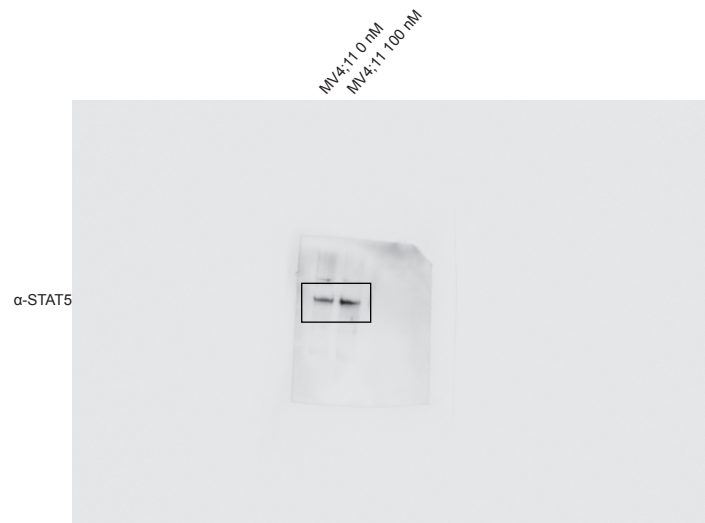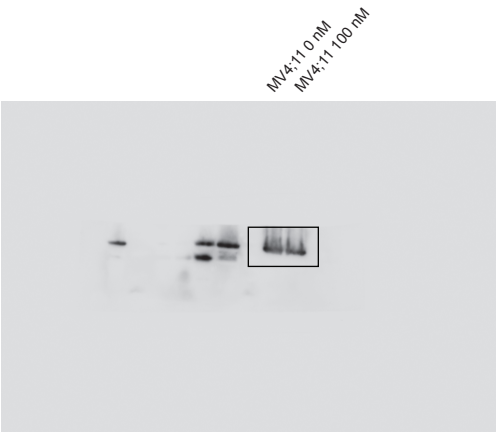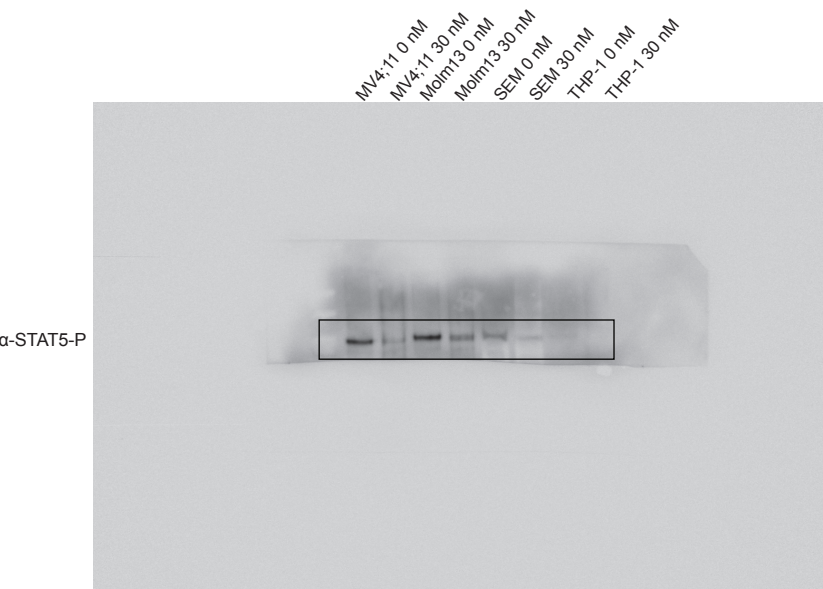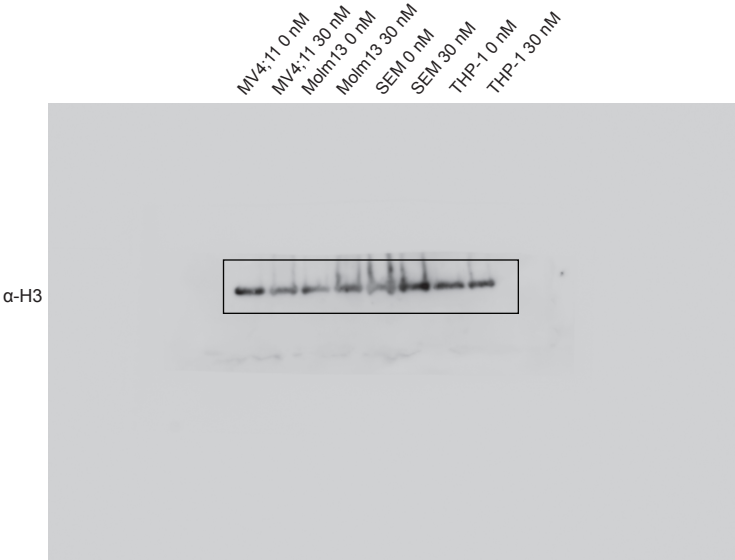

Supplement: Source data 2. [file elife-64960-data2.zip › source data folder 2/Figure 4 source data 17 4B right blot labels.pdf]

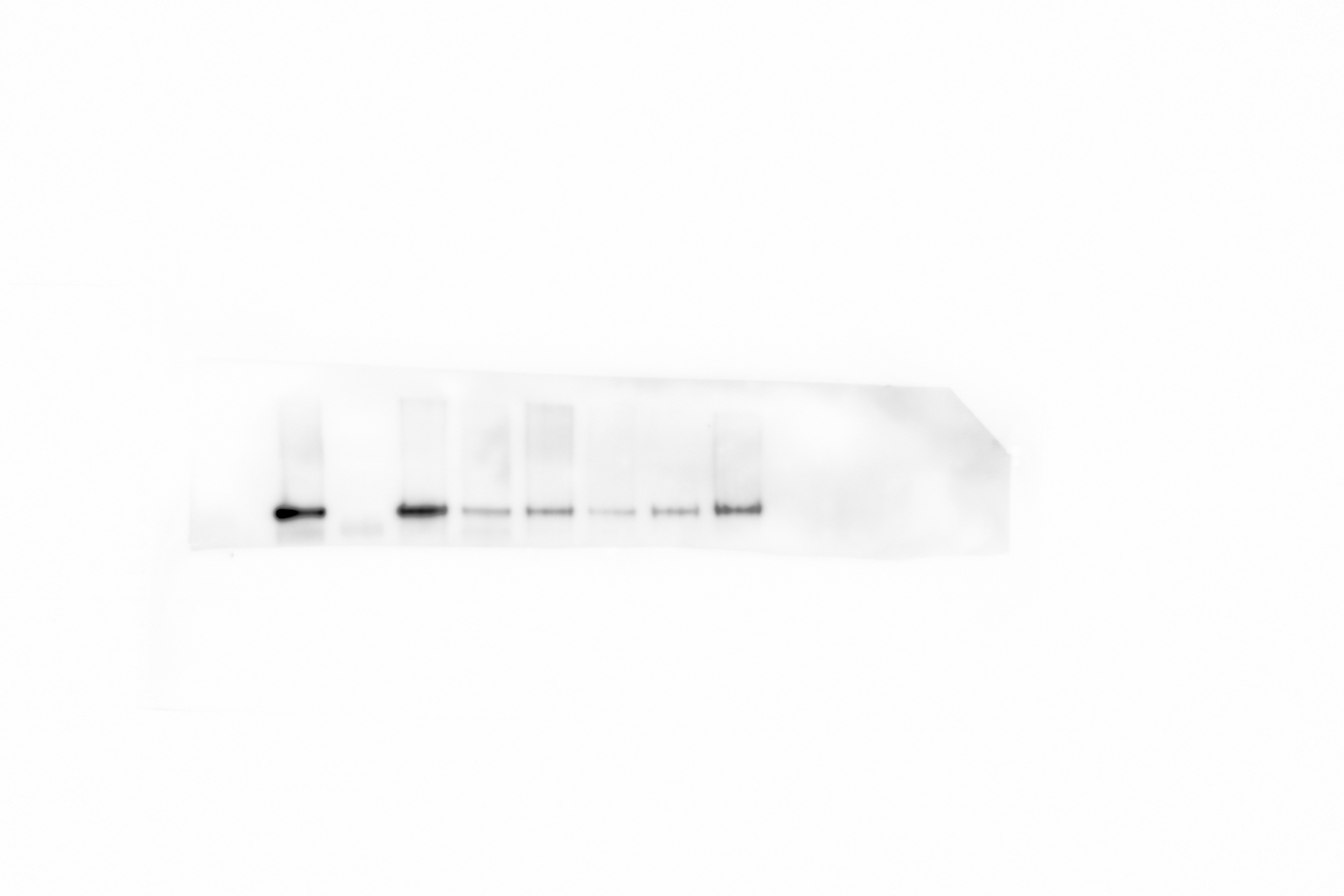

Supplement: Source data 2. [file elife-64960-data2.zip › source data folder 2/FIgure 4 source data 9 4B p-STAT5A pino.tif]

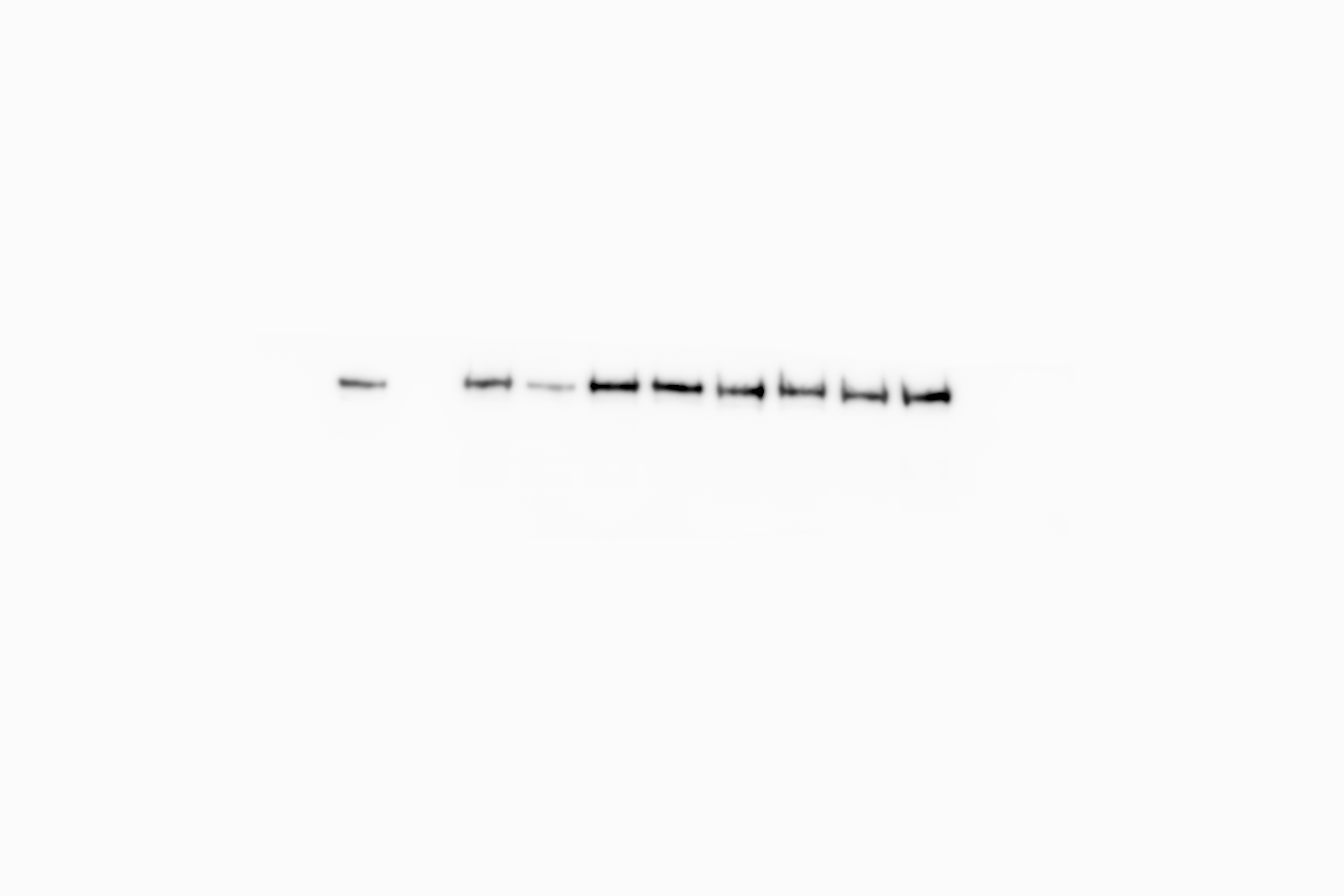

Supplement: Source data 2. [file elife-64960-data2.zip › source data folder 2/Figure 4 source data 6 4B HNRNPK pino.tif]

Supplementary Figure 4C

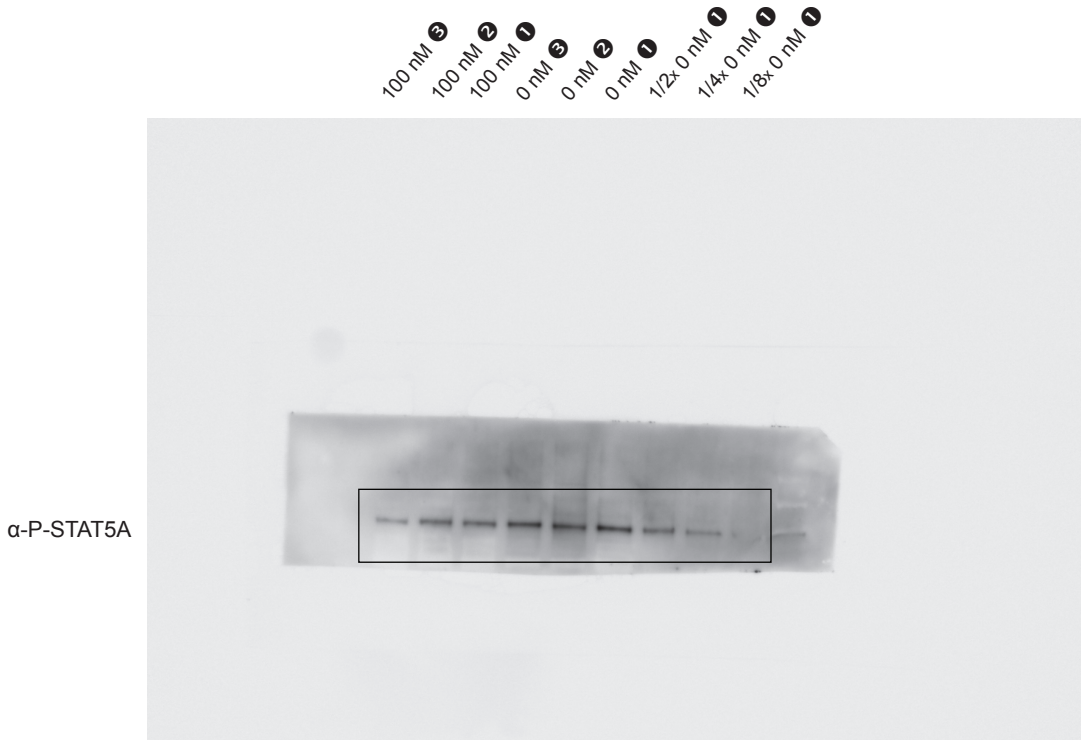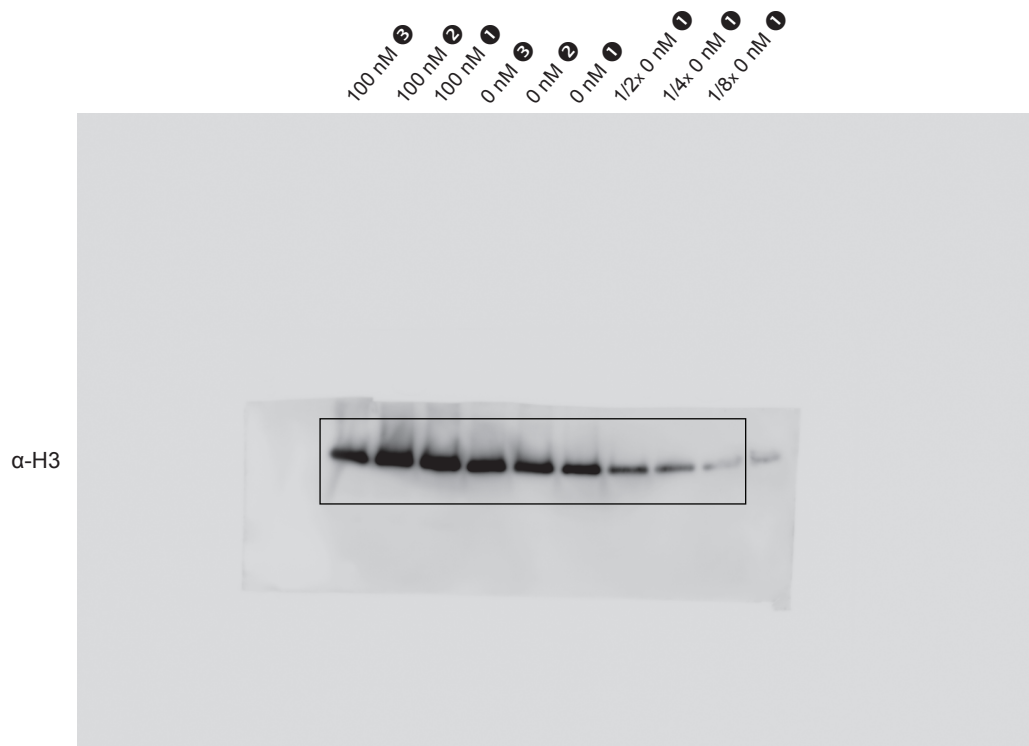

Supplement: Source data 2. [file elife-64960-data2.zip › source data folder 2/Figure 4 figure supplement 1 source data 11 4C blot labels.pdf]

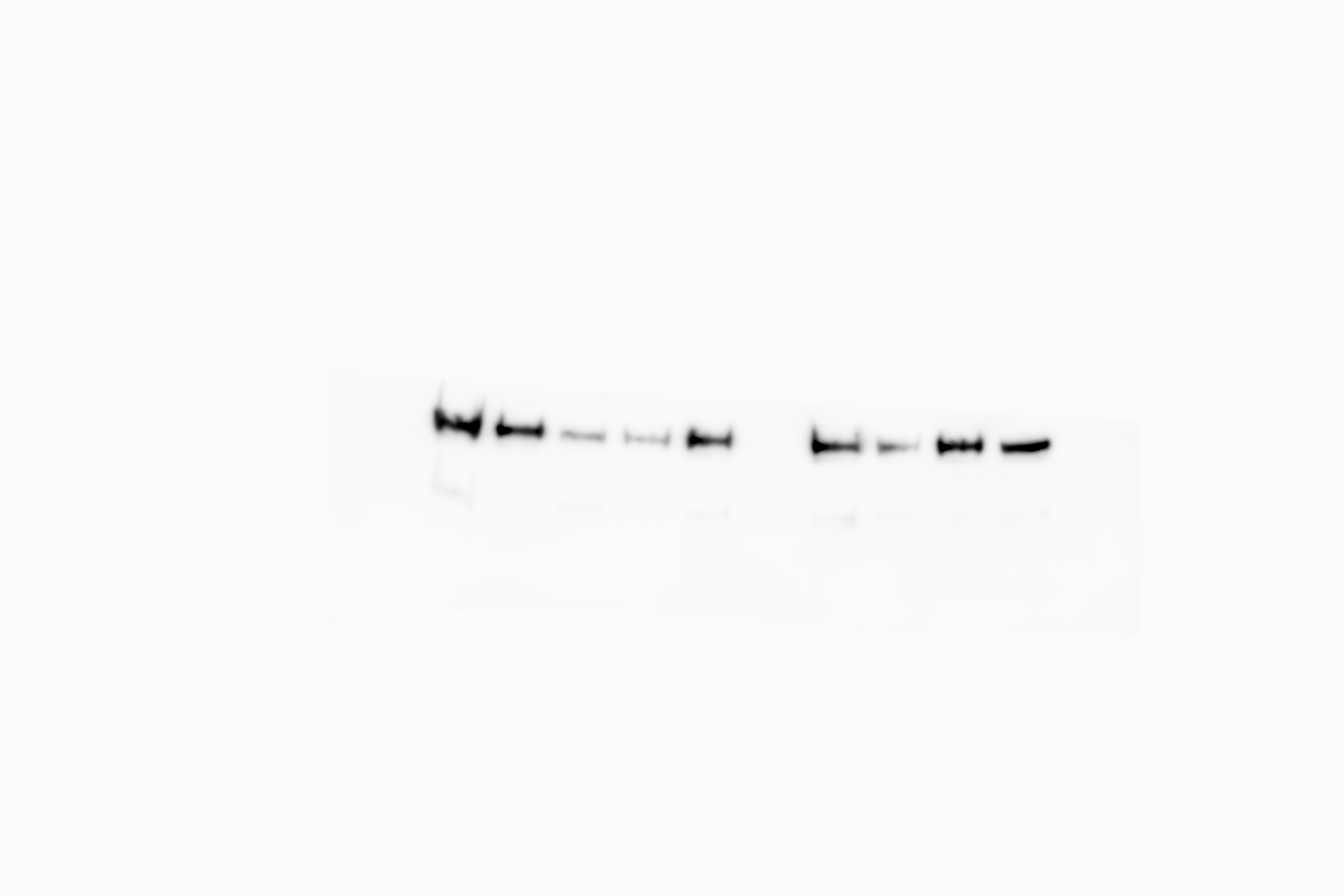

Supplement: Source data 2. [file elife-64960-data2.zip › source data folder 2/Figure 4 source data 5 4B HNRNPK MV4;11.tif]

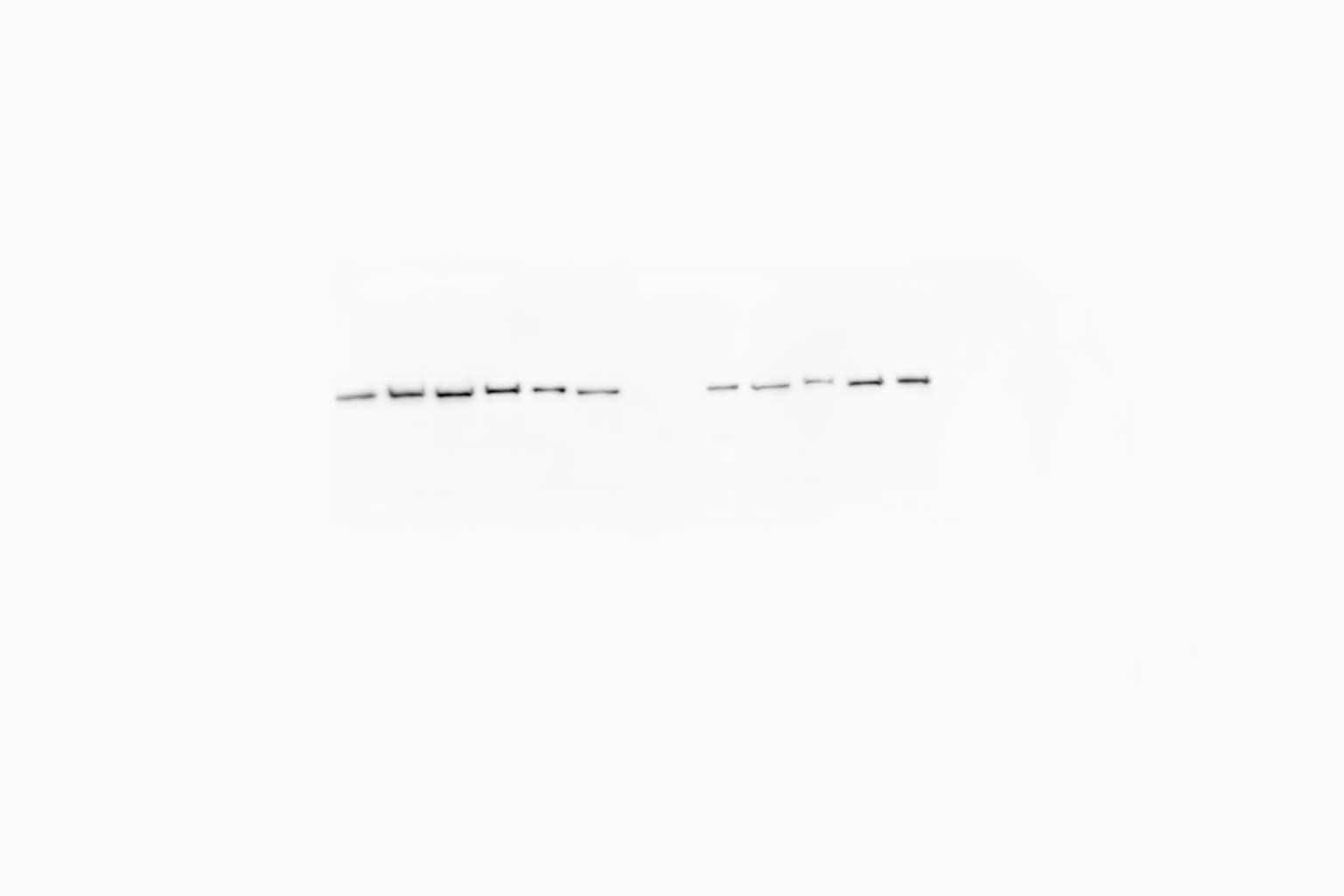

Supplement: Source data 2. [file elife-64960-data2.zip › source data folder 2/Figure 4 figure supplement 1 source data 9 S4J HNRNPK.tif]

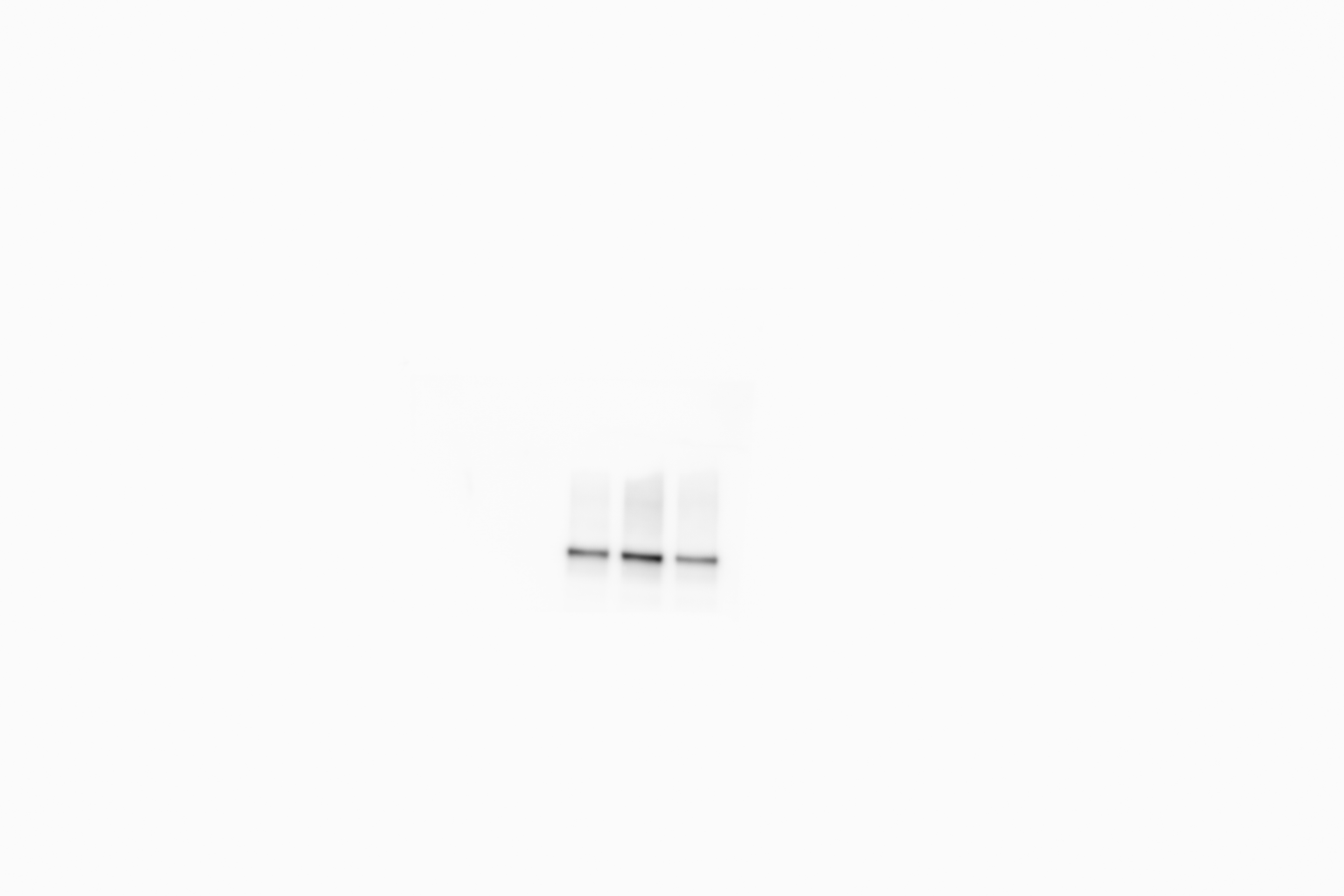

Supplement: Source data 2. [file elife-64960-data2.zip › source data folder 2/Figure 4 figure supplement 1 source data 7 S4I p-STAT5.tif]

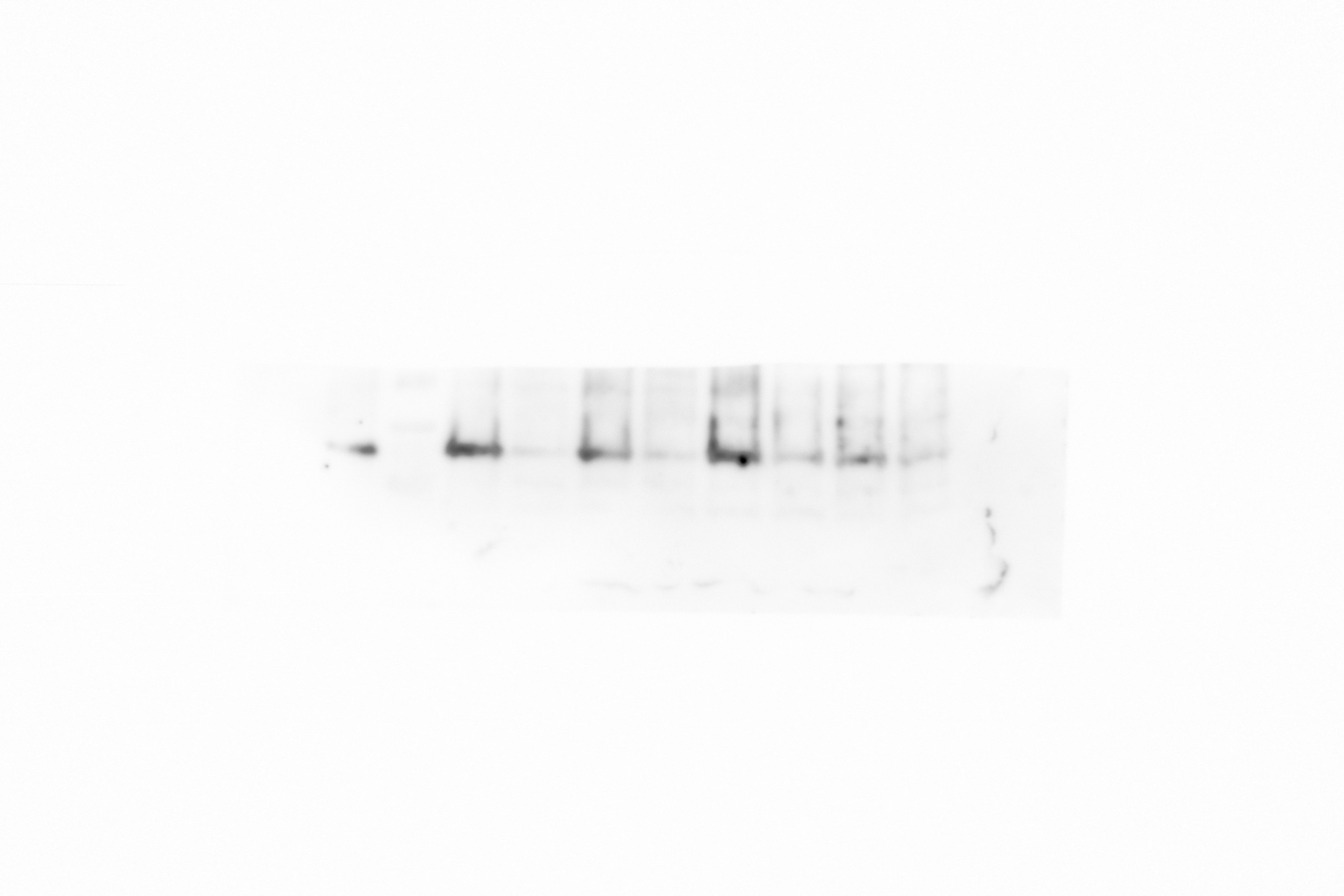

Supplement: Source data 2. [file elife-64960-data2.zip › source data folder 2/Figure 4 source data 4 4B H3K79me2 pino.tif]

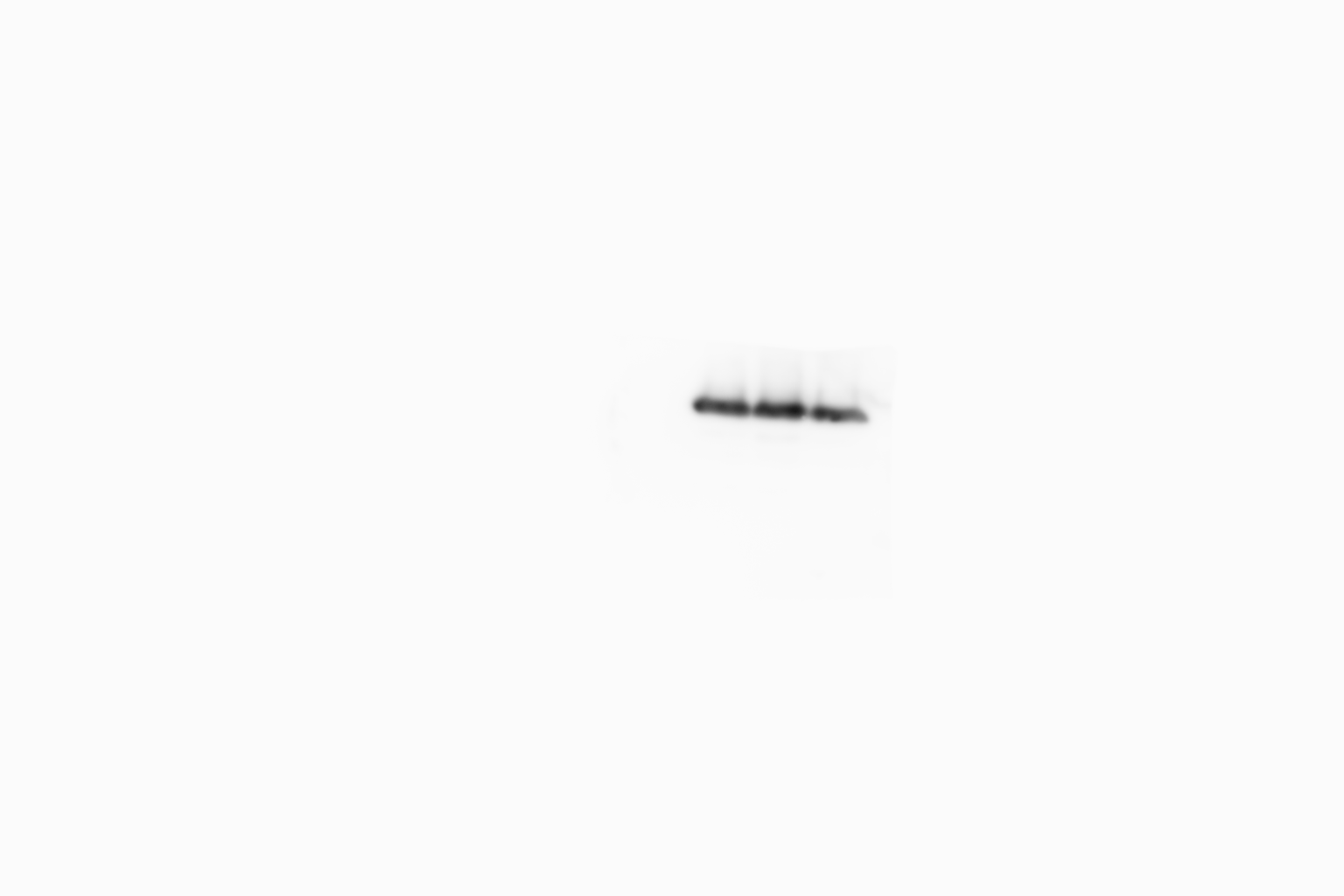

Supplement: Source data 2. [file elife-64960-data2.zip › source data folder 2/Figure 4 figure supplement 1 source data 6 S4I H3.tif]

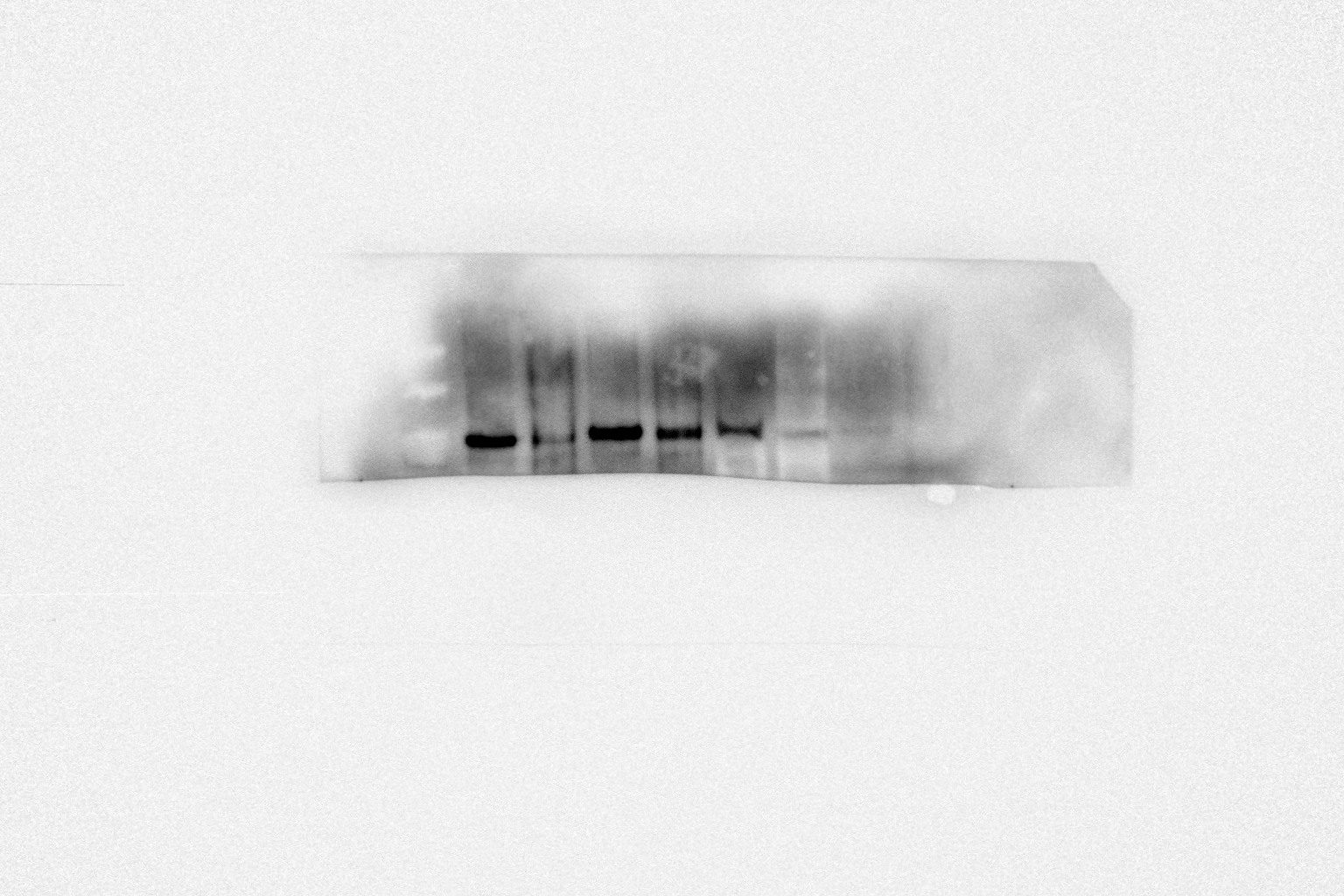

Supplement: Source data 2. [file elife-64960-data2.zip › source data folder 2/Figure 4 source data 8 4B p-STAT5 tandutinib.tif]

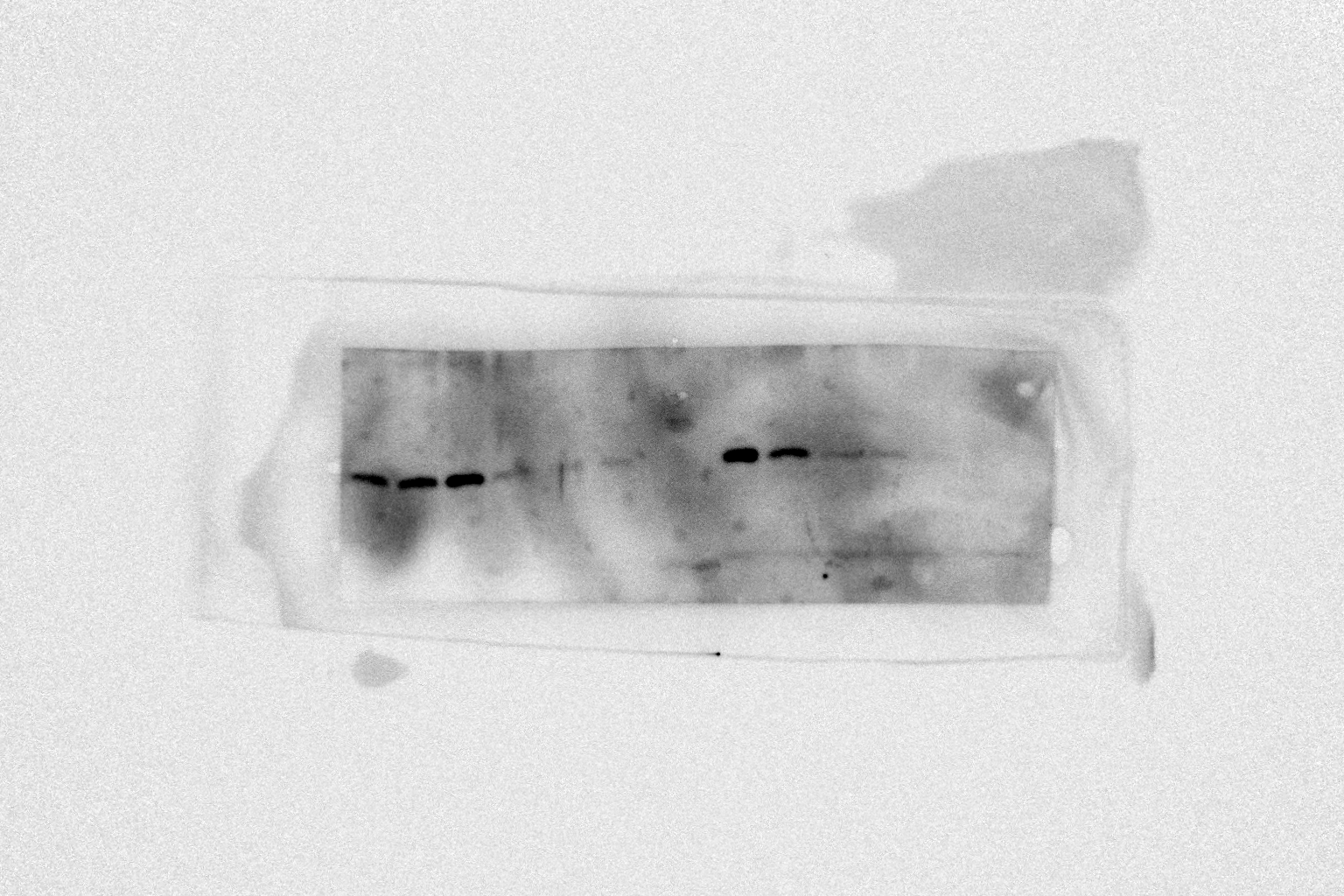

Supplement: Source data 2. [file elife-64960-data2.zip › source data folder 2/Figure 4 figure supplement 1 source data 8 S4J H3K79me2.tif]

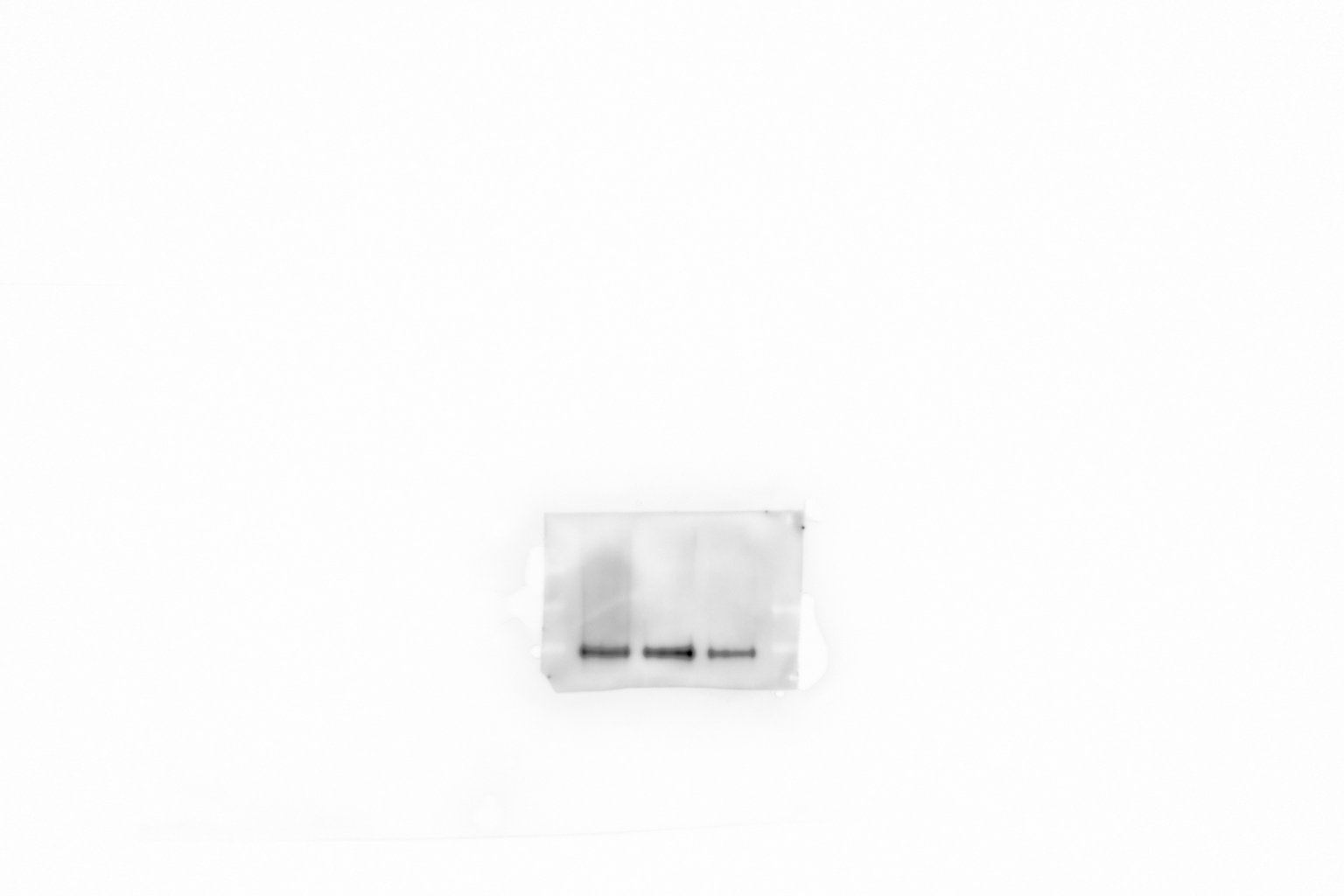

Supplement: Source data 2. [file elife-64960-data2.zip › source data folder 2/FIgure 4 source data 15 4J p-STAT5 EOL1.tif]

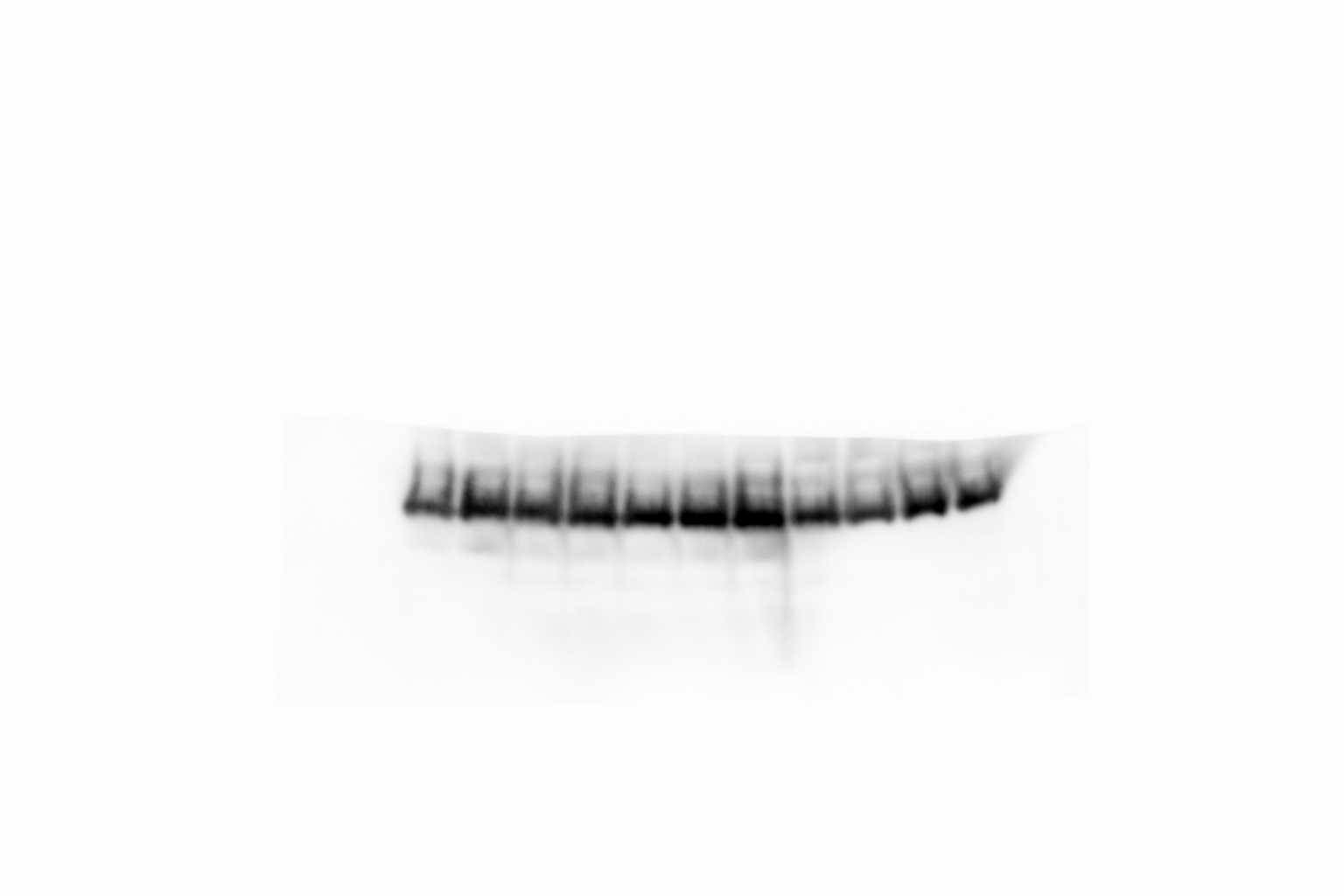

Supplement: Source data 2. [file elife-64960-data2.zip › source data folder 2/Figure 4 source data 11 4J H2B.tif]

Supplementary Figure 4I

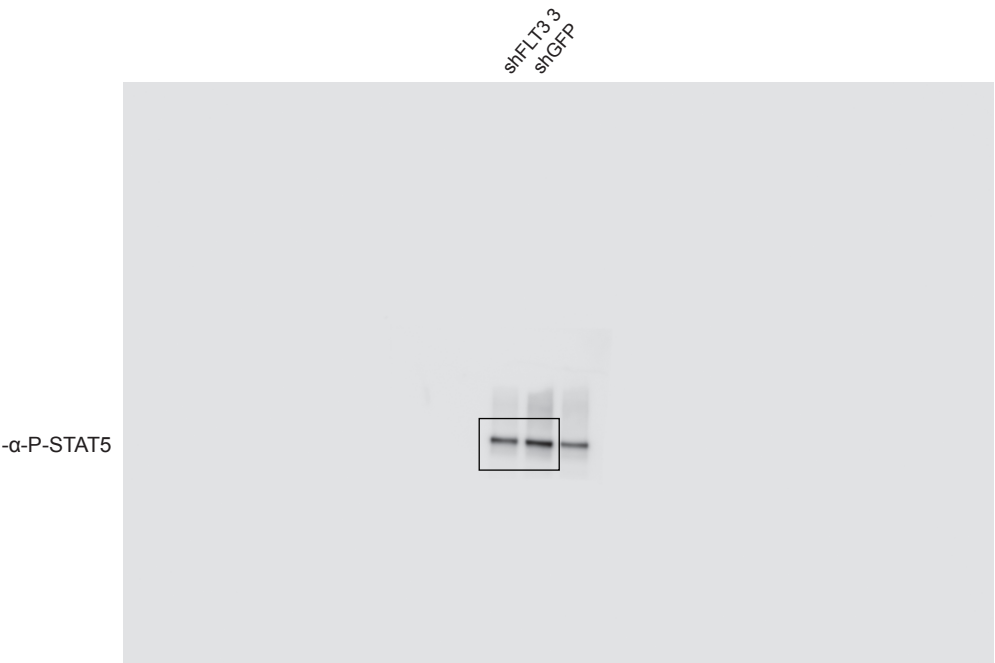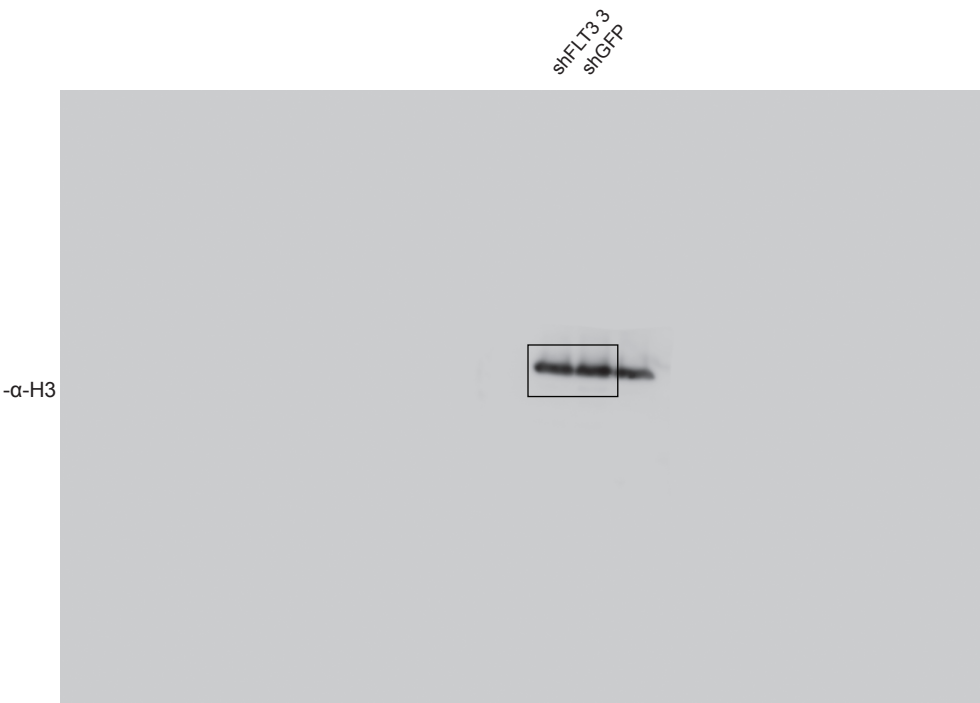

Supplement: Source data 2. [file elife-64960-data2.zip › source data folder 2/Figure 4 figure supplement 1 source data 12 4I blot labels.pdf]

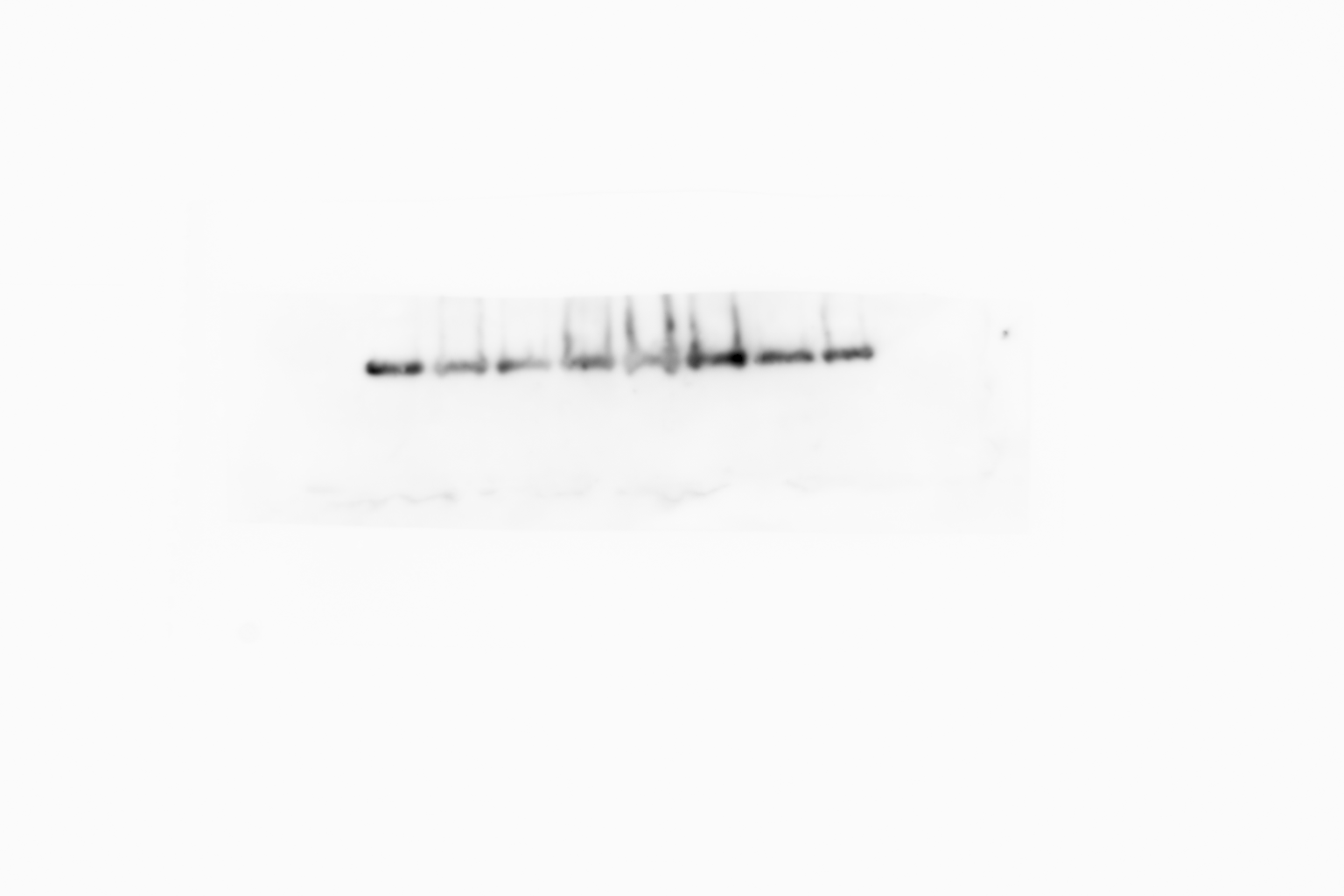

Supplement: Source data 2. [file elife-64960-data2.zip › source data folder 2/Figure 4 source data 2 4B H3 tandutinib.tif]

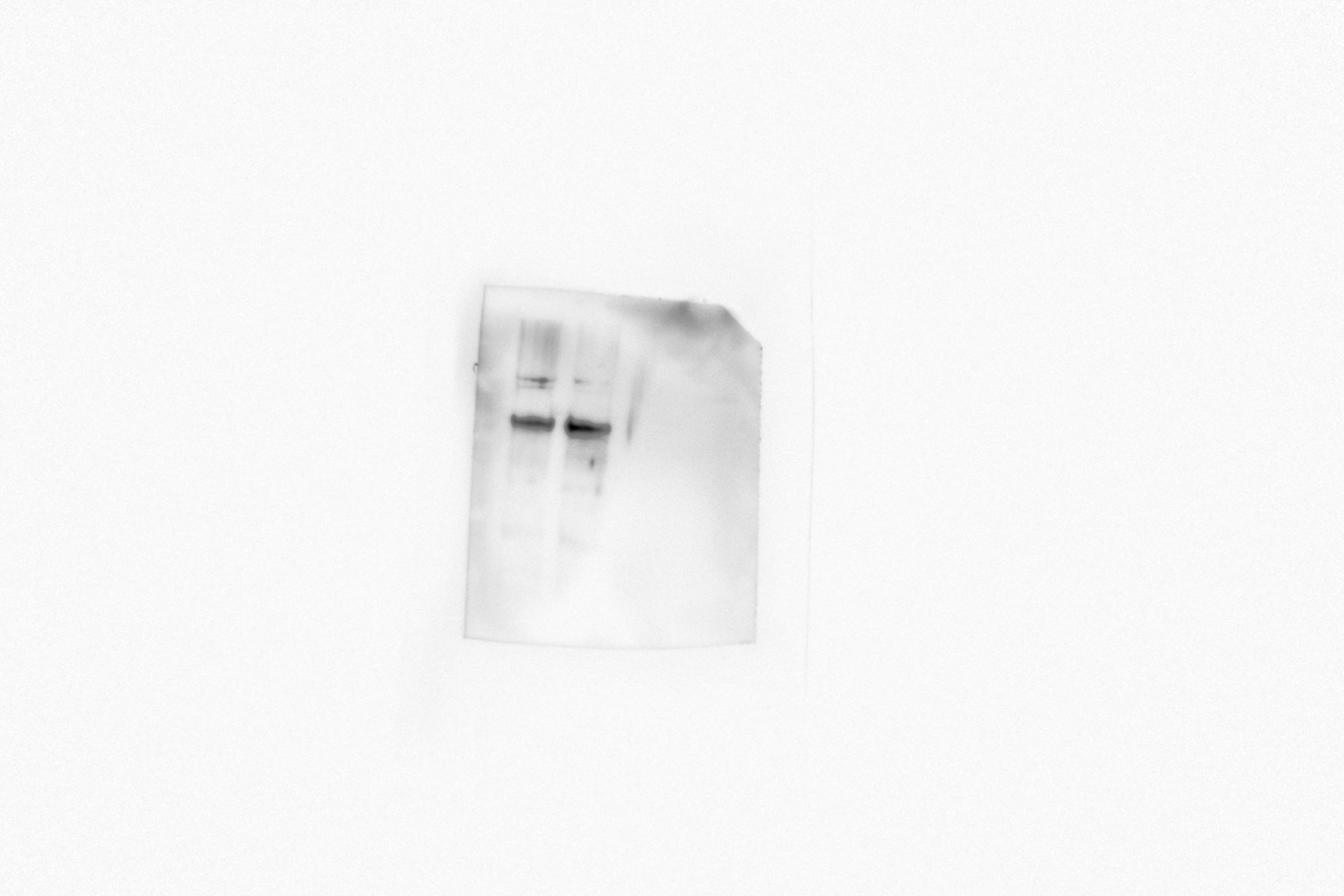

Supplement: Source data 2. [file elife-64960-data2.zip › source data folder 2/Figure 4 source data 10 4B STAT5.tif]

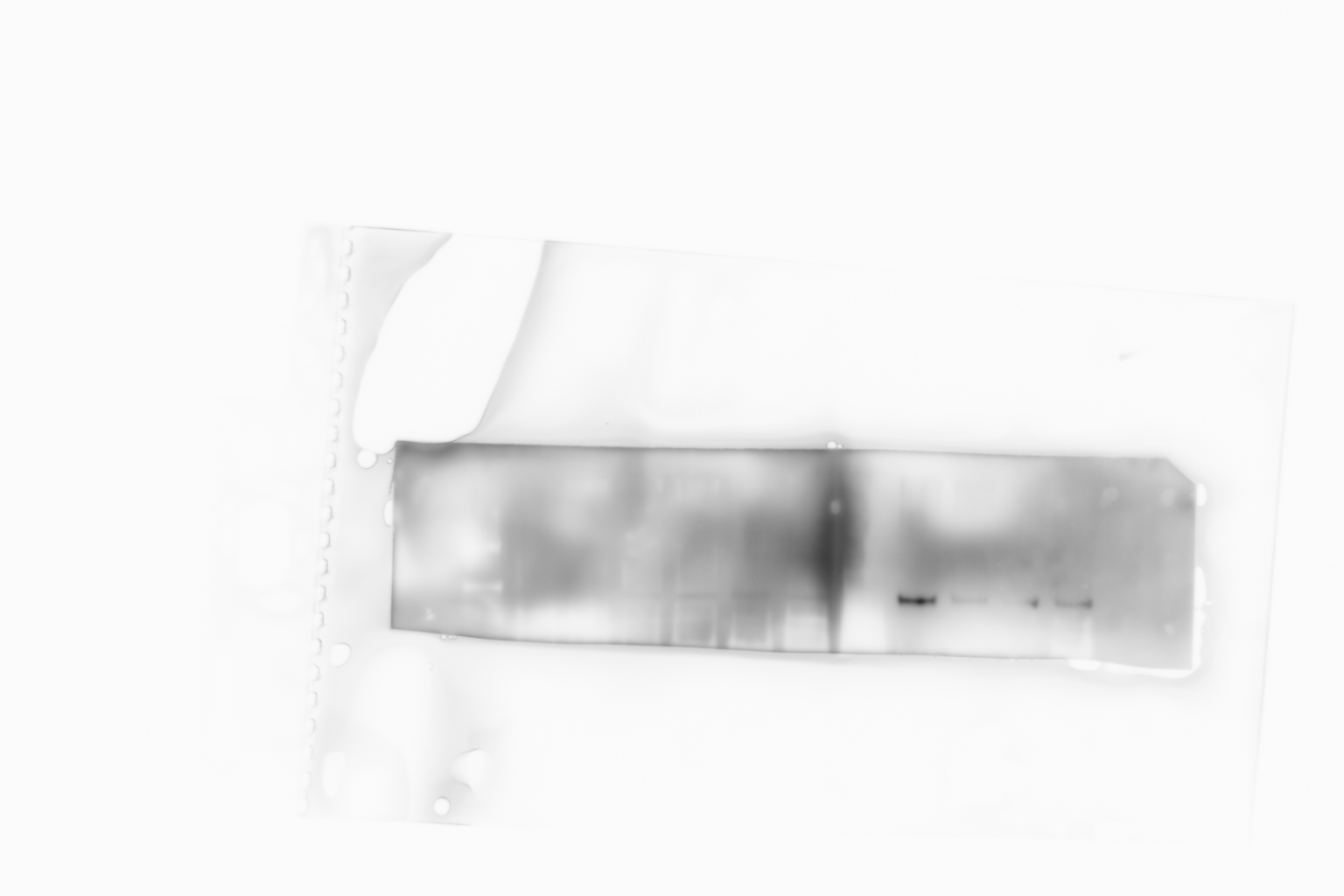

Supplement: Source data 2. [file elife-64960-data2.zip › source data folder 2/Figure 4 source data 15 4J p-STAT5 PL21.tif]

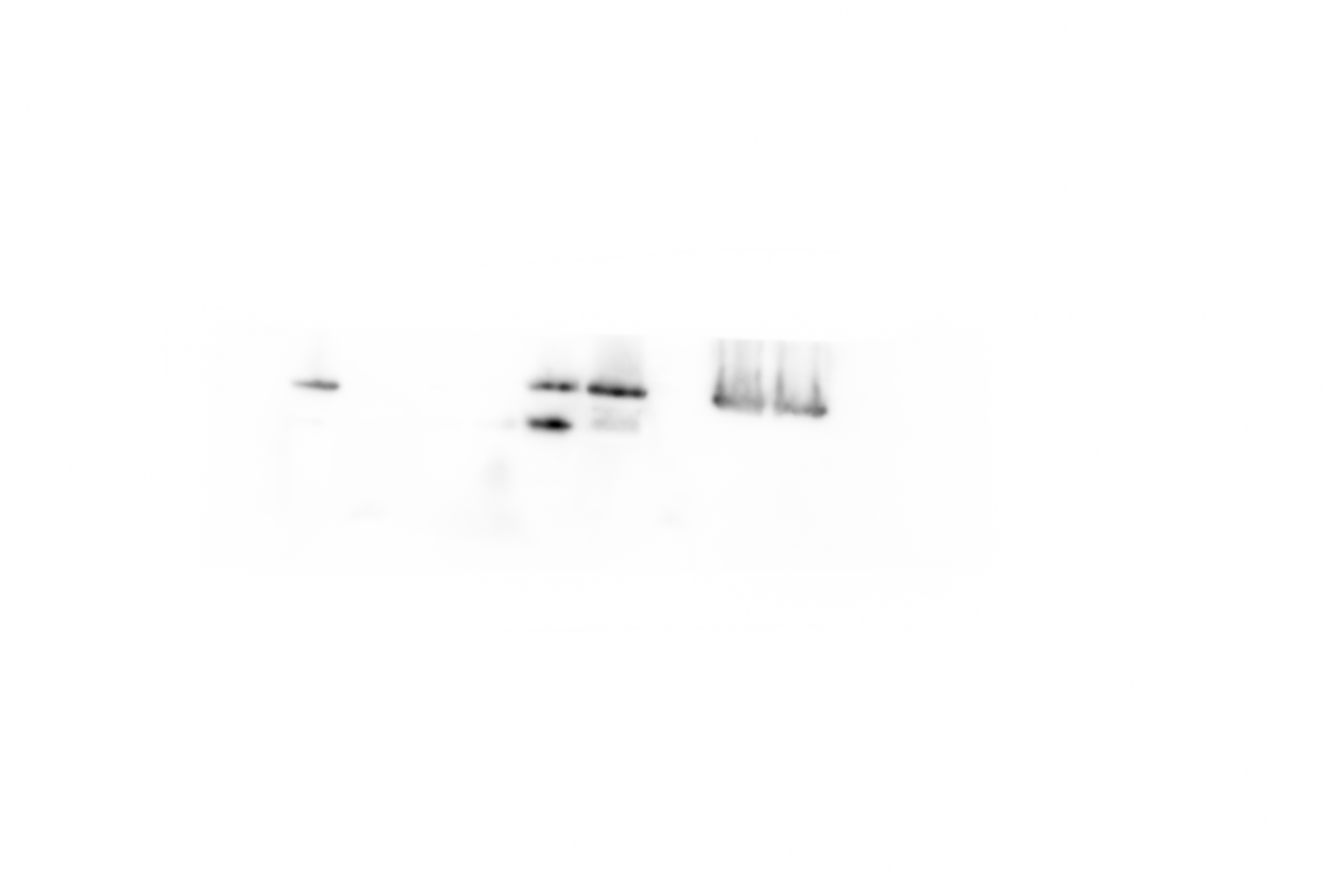

Supplement: Source data 2. [file elife-64960-data2.zip › source data folder 2/Figure 4 source data 1 4B H3 left.tif]

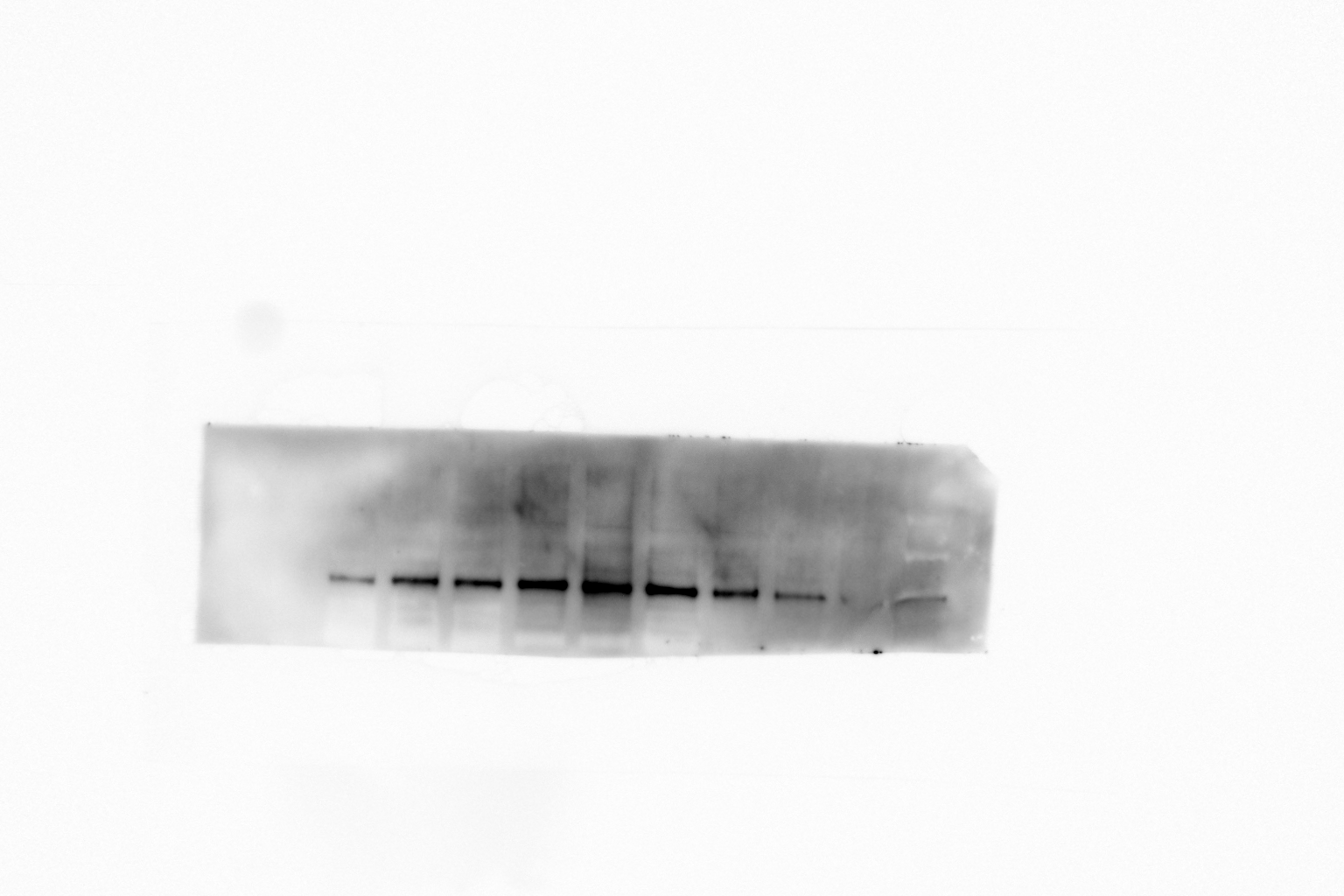

Supplement: Source data 2. [file elife-64960-data2.zip › source data folder 2/Figure 4 figure supplement 1 source data 5 S4C p-STAT5.tif]

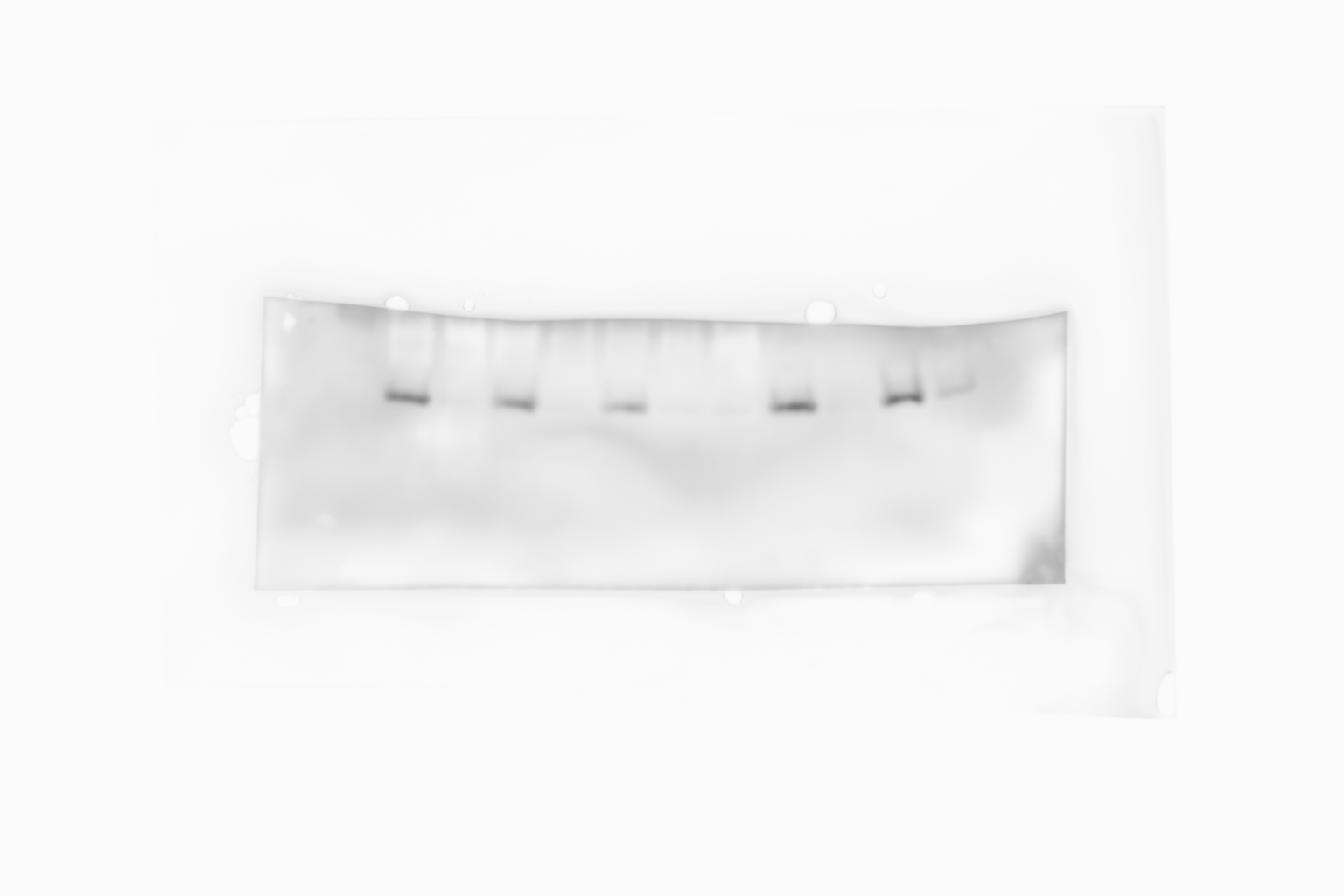

Supplement: Source data 2. [file elife-64960-data2.zip › source data folder 2/Figure 4 source data 13 4J H3K79me2 PL21.tif]

Figure 4J

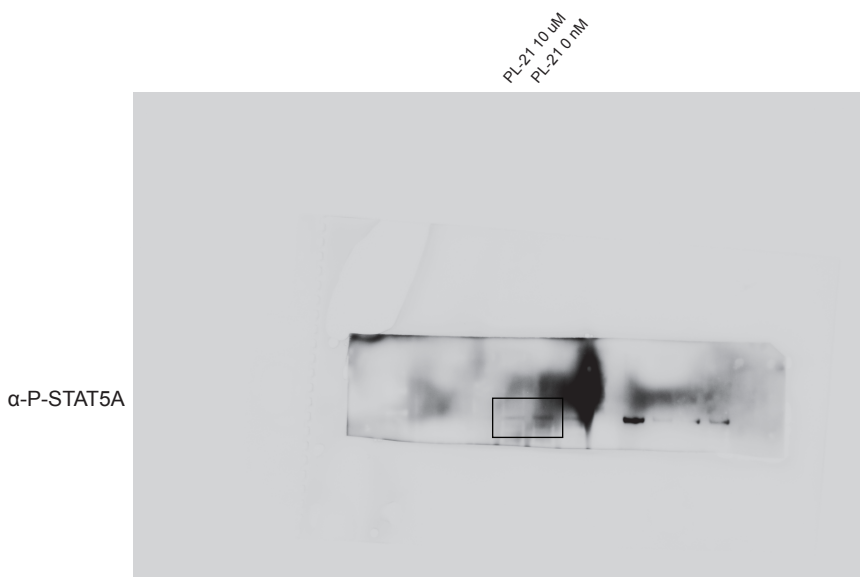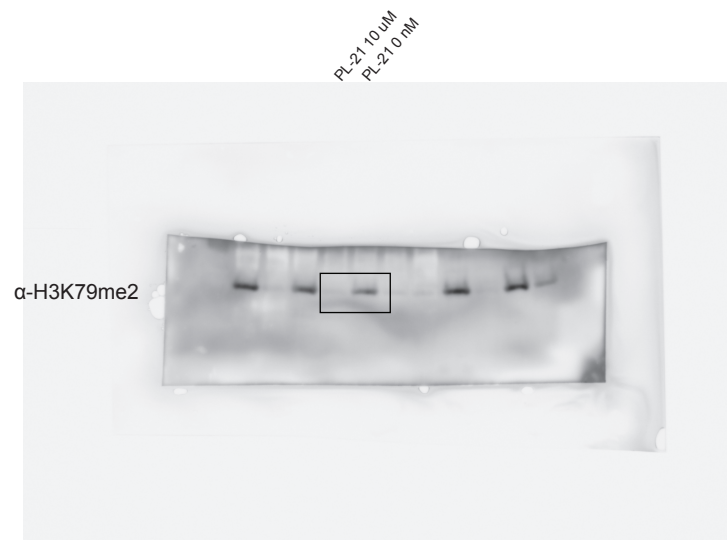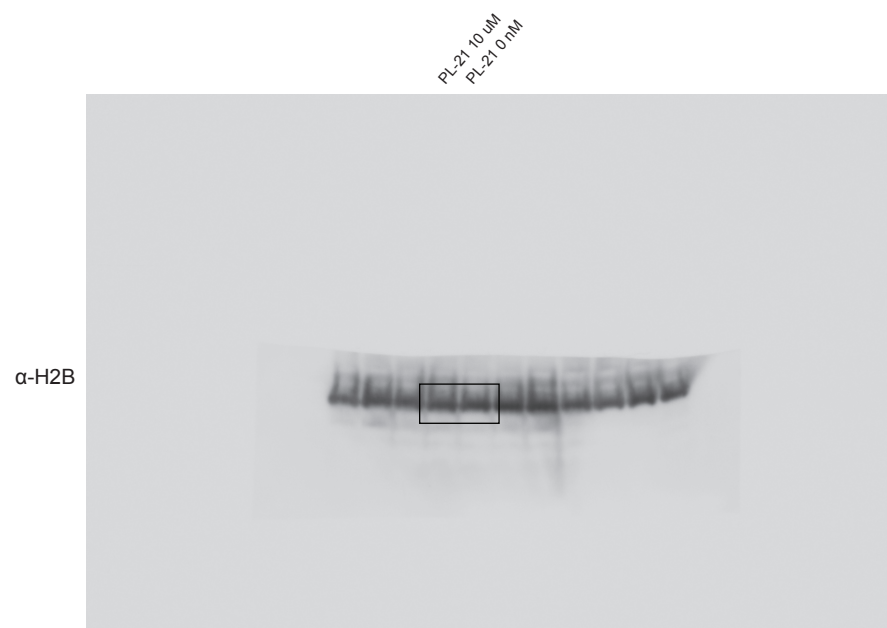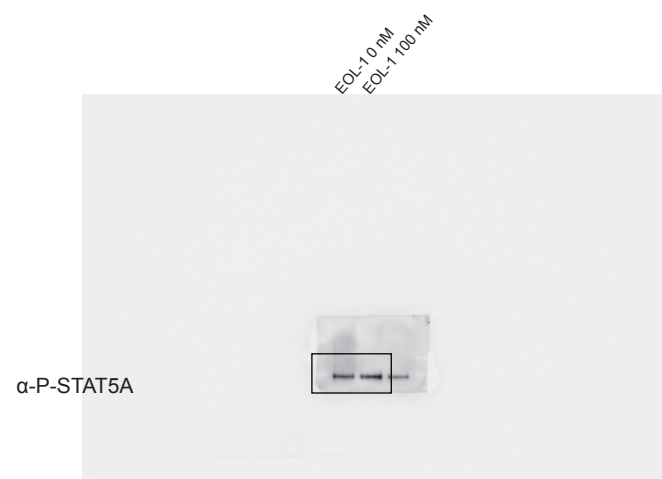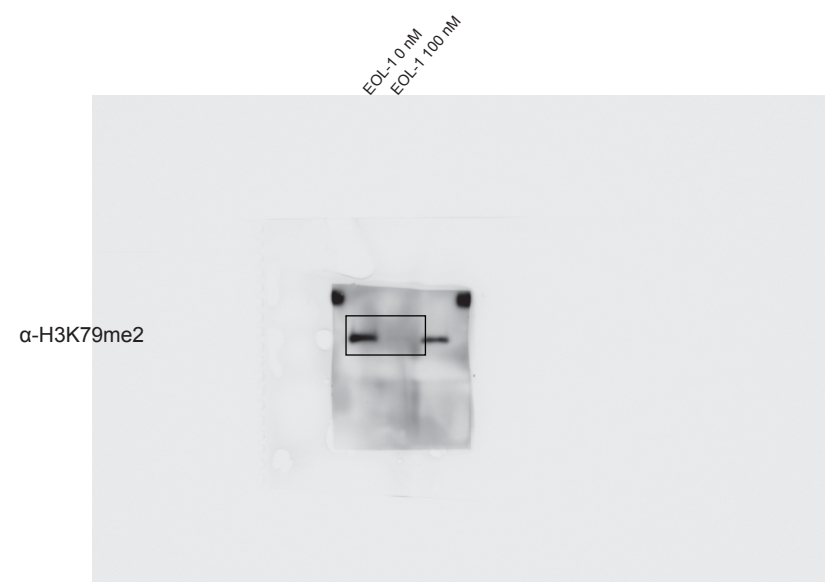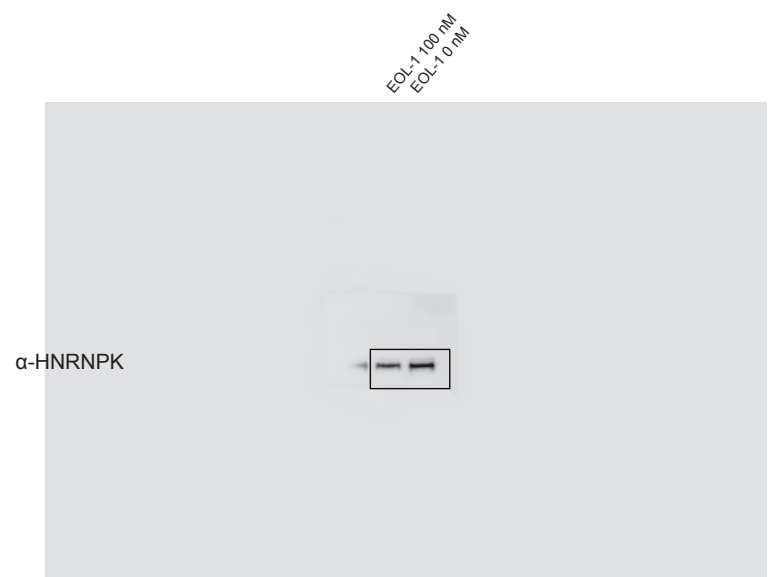

Supplement: Source data 2. [file elife-64960-data2.zip › source data folder 2/Figure 4 source data 18 4J blot labels.pdf]

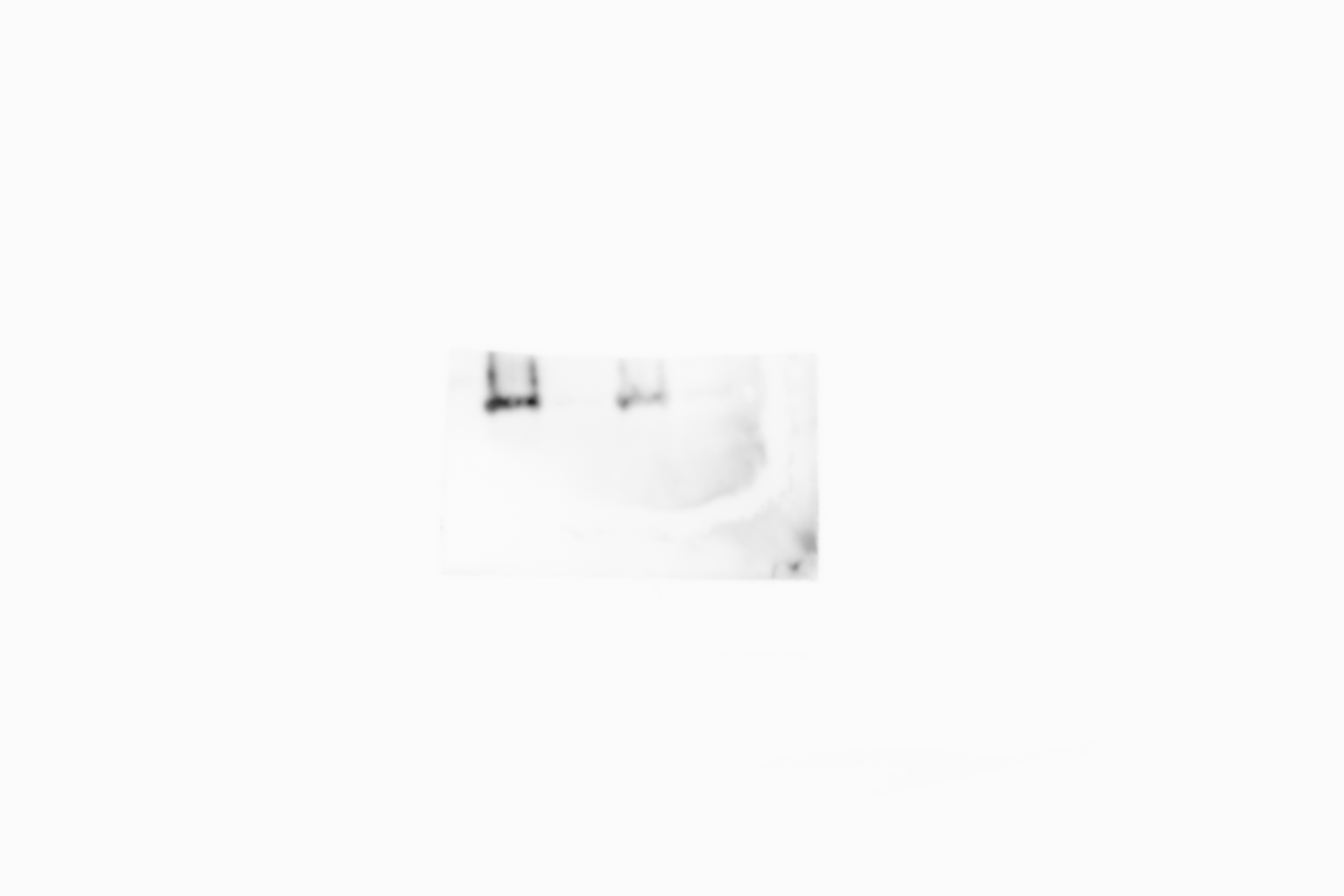

Supplement: Source data 2. [file elife-64960-data2.zip › source data folder 2/Figure 4 source data 3 4B H3K79me2 MV4;11.tif]

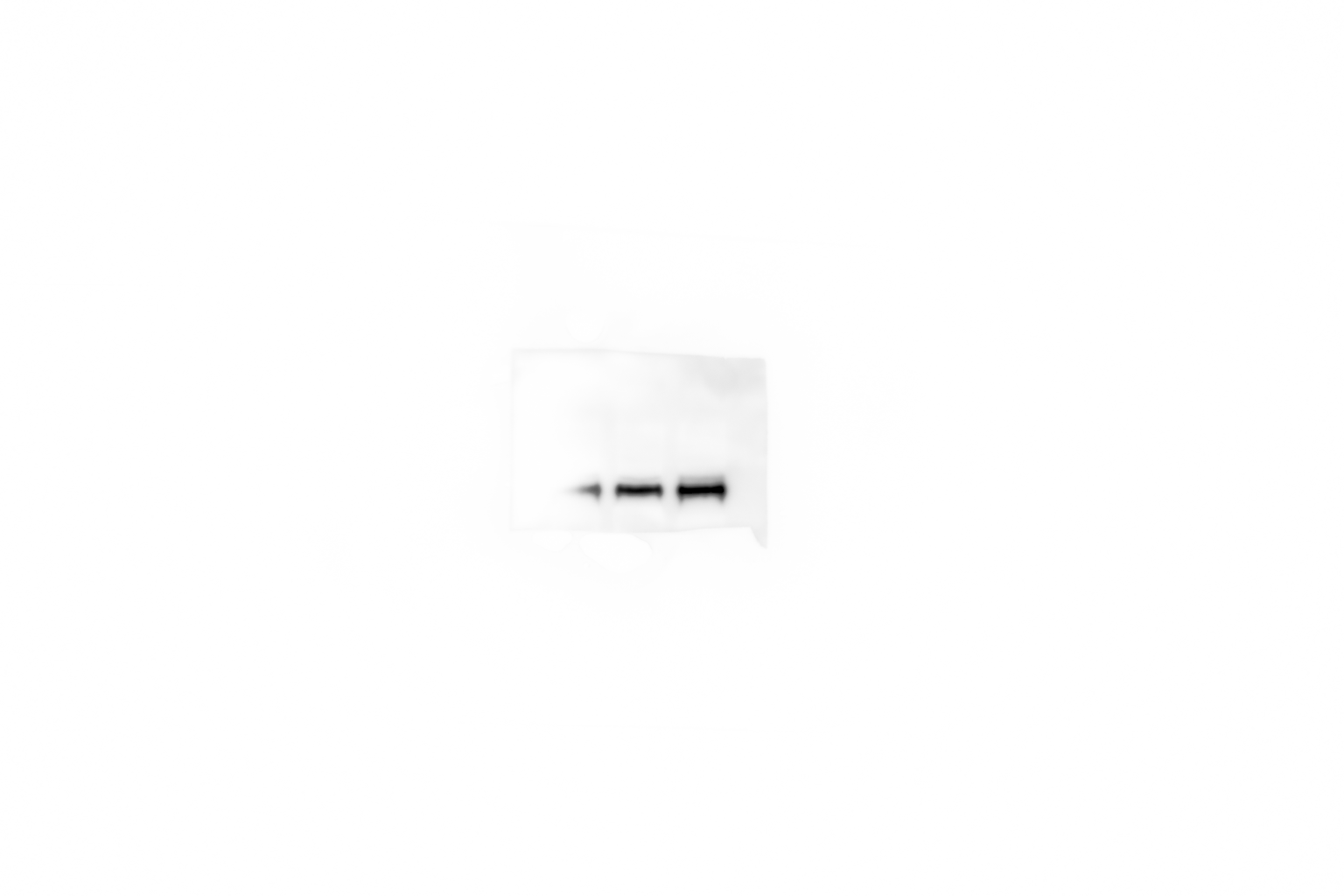

Supplement: Source data 2. [file elife-64960-data2.zip › source data folder 2/Figure 4 source data 14 4J HNRNPK.tif]

Supplementary Figure 4J

MV4;11 100 nM pino  
day 0 day 1 day 2 day 3 day 4

-α-H3K79me2

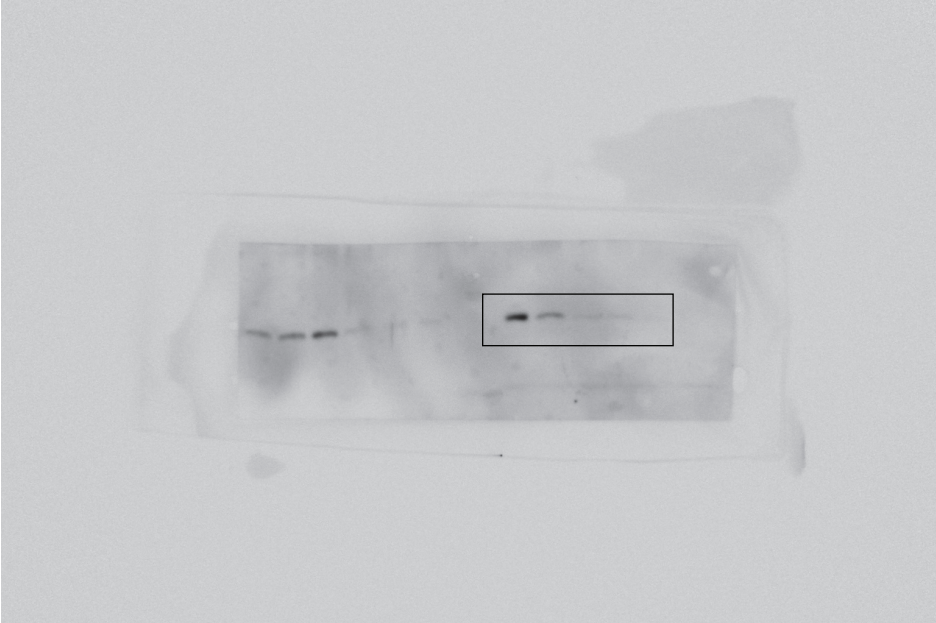

MV4;11 100 nM pino  
day 0 day 1 day 2 day 3 day 4

-α-HNRNPK

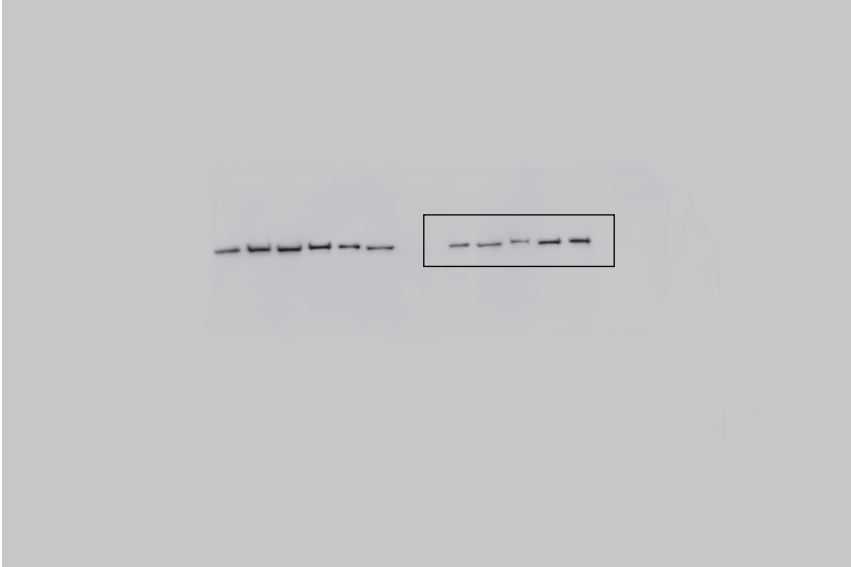

Supplement: Source data 2. [file elife-64960-data2.zip › source data folder 2/Figure 4 figure supplement 1 source data 13 4J blot labels.pdf]

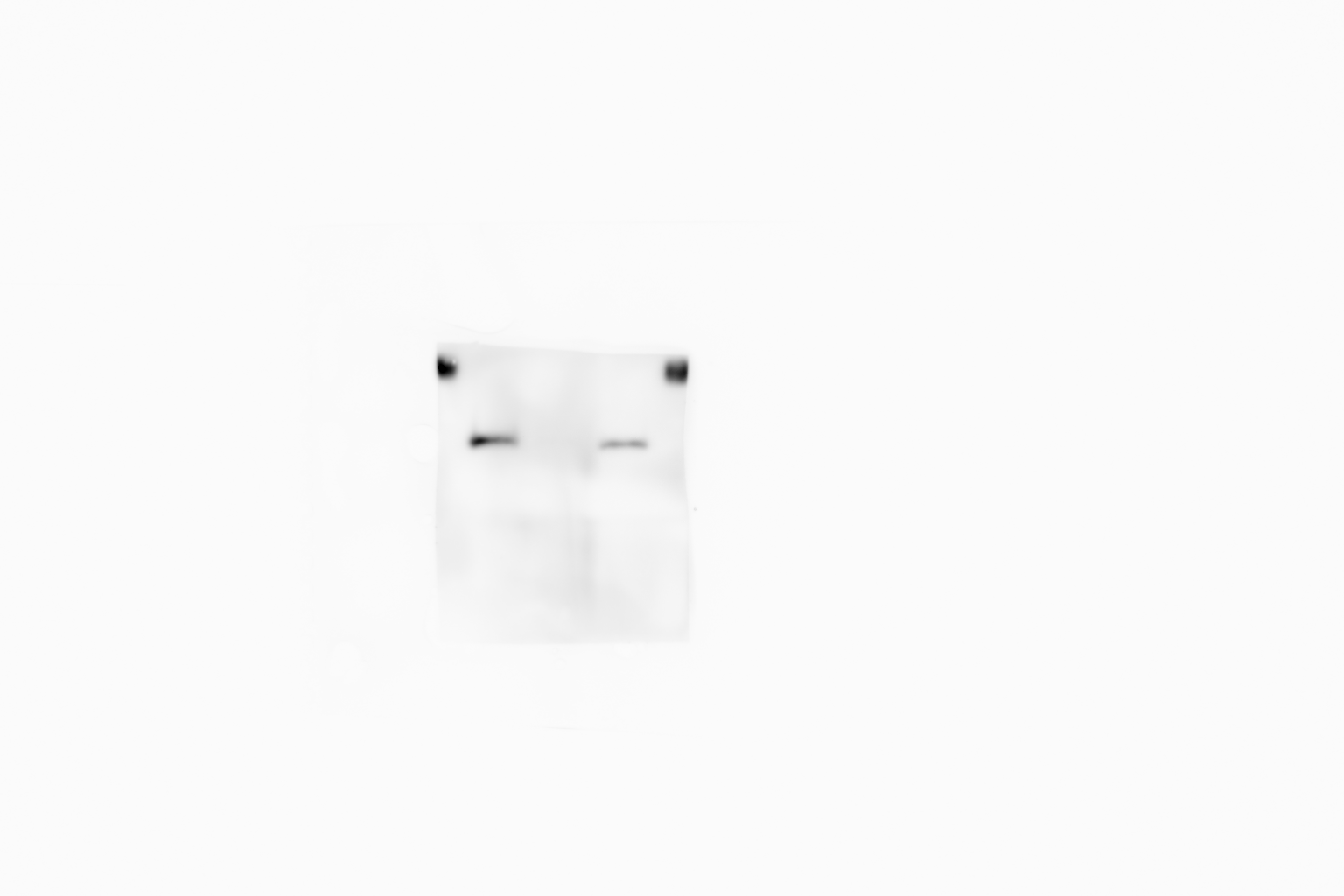

Supplement: Source data 2. [file elife-64960-data2.zip › source data folder 2/Figure 4 source data 12 4J H3K79me2 EOL1.tif]

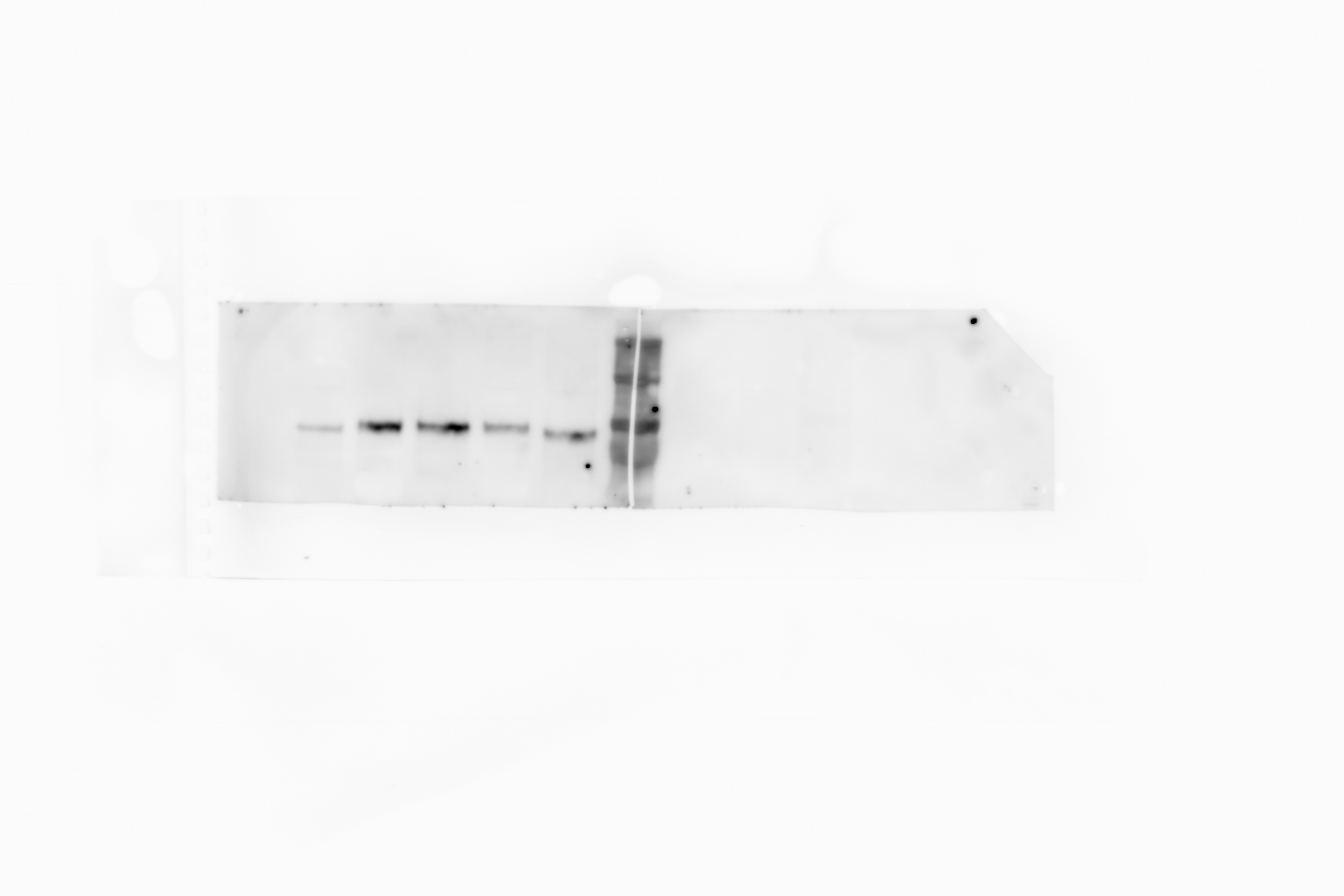

Supplement: Source data 3. [file elife-64960-data3.zip › source data folder 3/Figure 5 figure supplement 1 source data 2 S5A STAT5.tif]

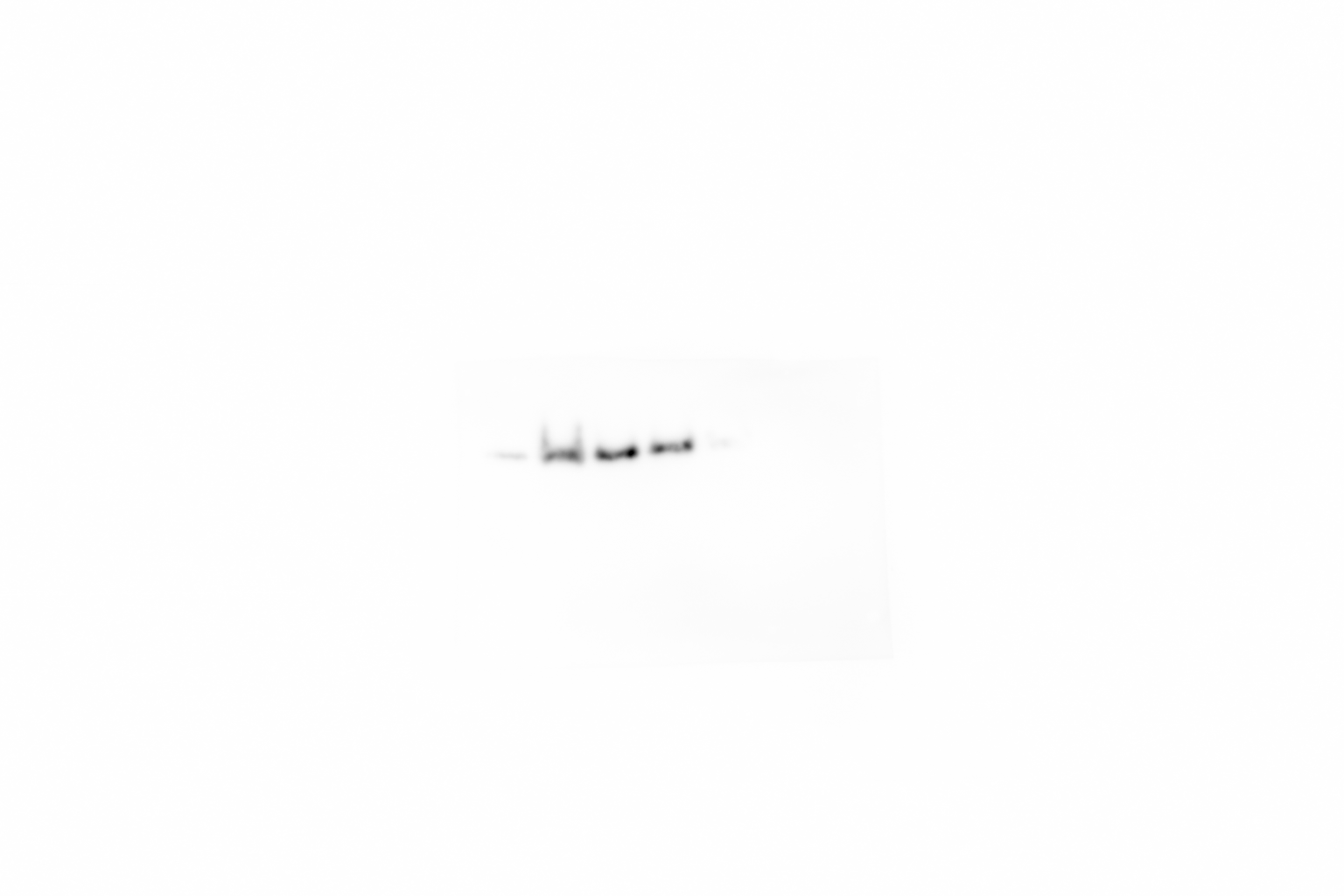

Supplement: Source data 3. [file elife-64960-data3.zip › source data folder 3/Figure 7 figure supplement 1 source data 4 S7B H3K4me3.tif]

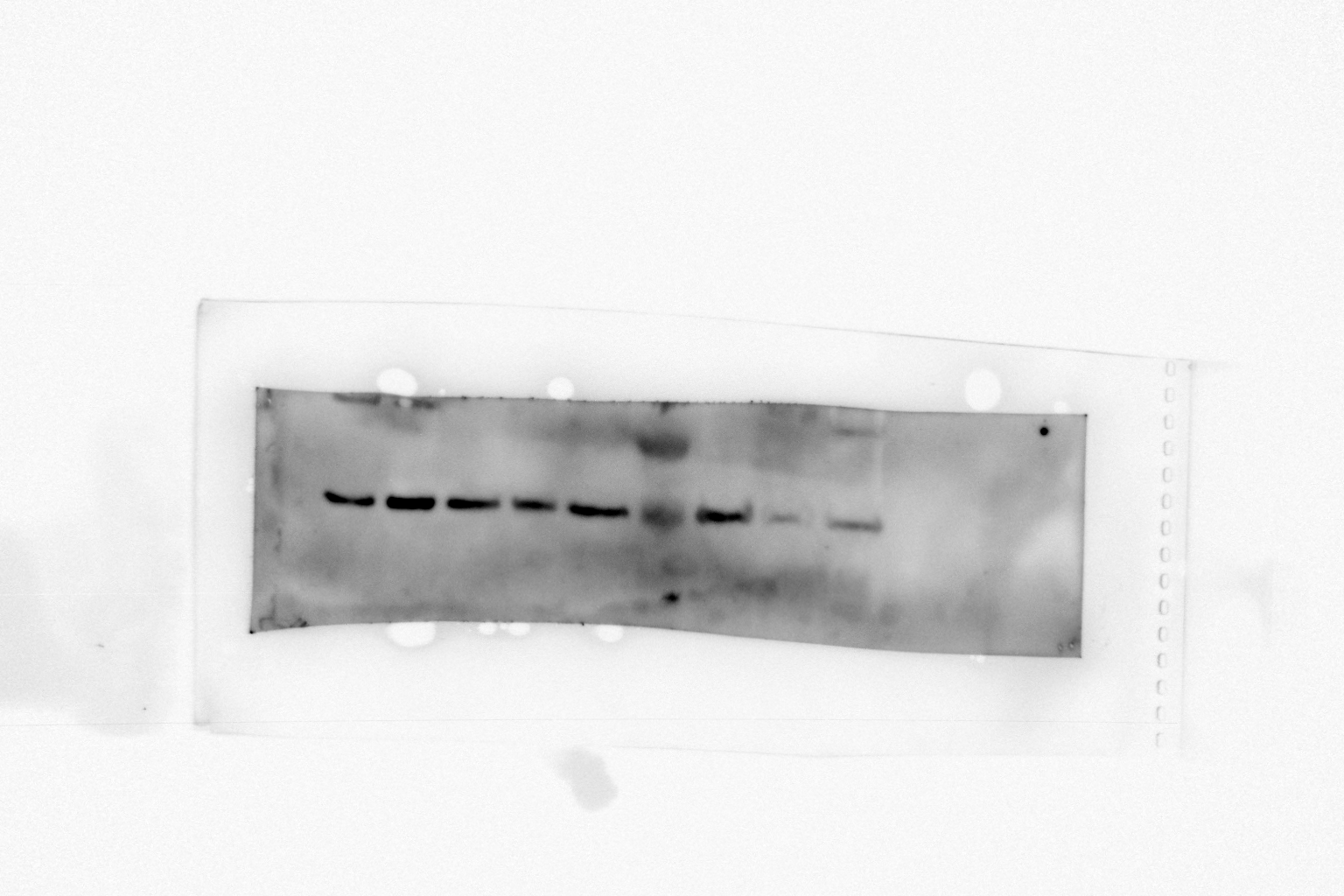

Supplement: Source data 3. [file elife-64960-data3.zip › source data folder 3/Figure 5 figure supplement 1 source data 1 S5A GAPDH.tif]

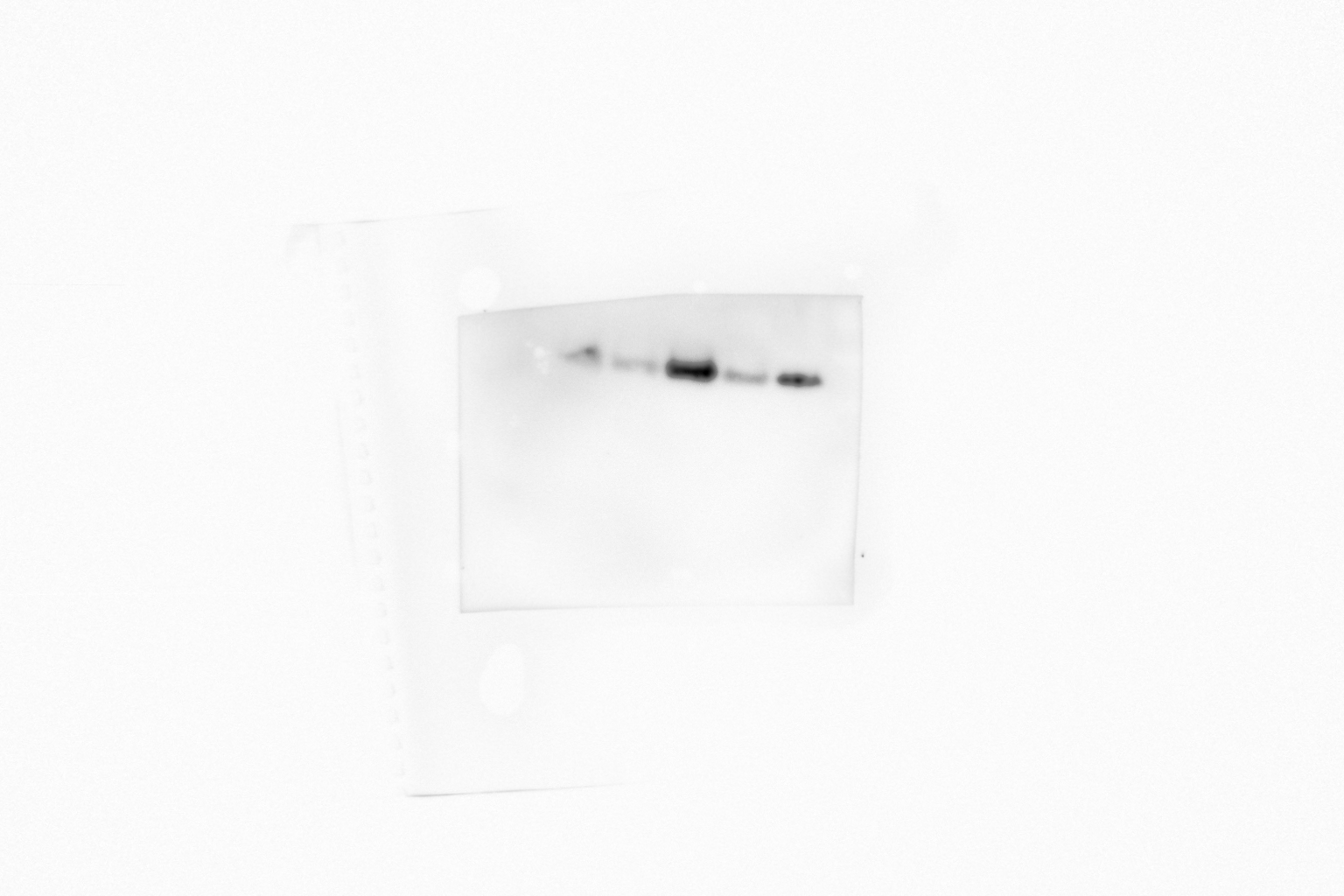

Supplement: Source data 3. [file elife-64960-data3.zip › source data folder 3/Figure 6 source data 5 6D H3K27me3.tif]

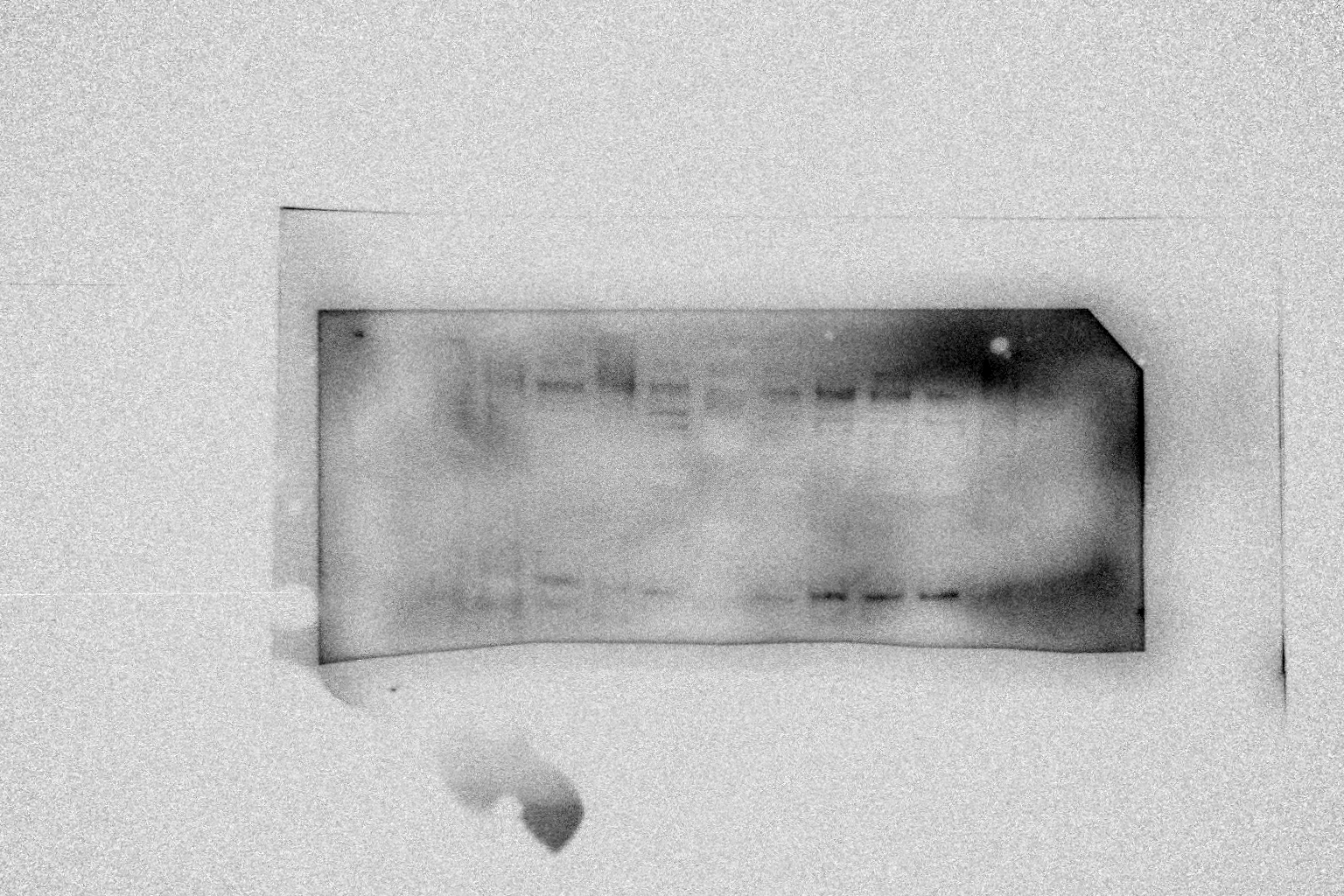

Supplement: Source data 3. [file elife-64960-data3.zip › source data folder 3/Figure 7 figure supplement 1 source data 3 S7B GAPDH.tif]

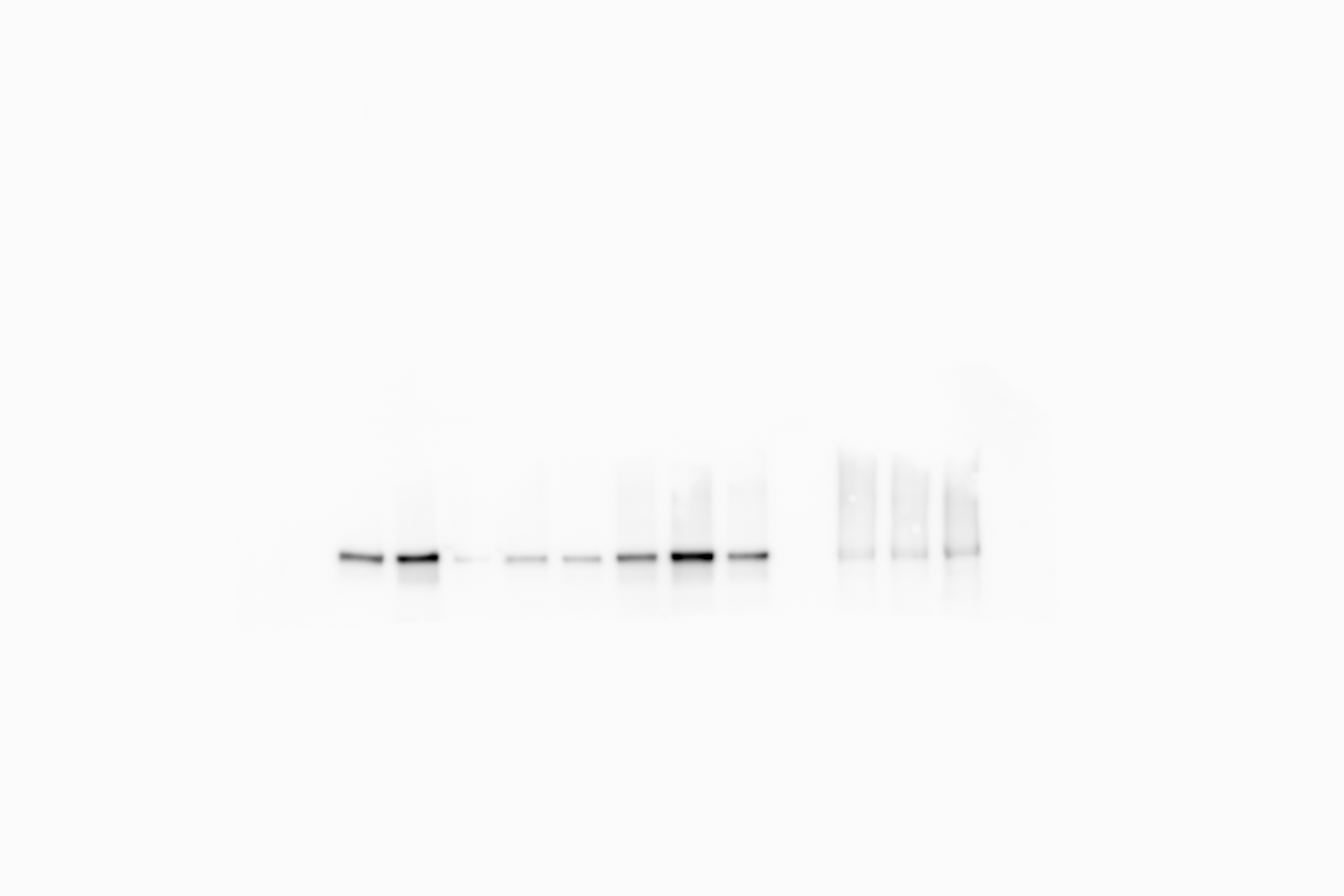

Supplement: Source data 3. [file elife-64960-data3.zip › source data folder 3/Figure 6 figure supplement 1 source data 2 S6C p-STAT5.tif]

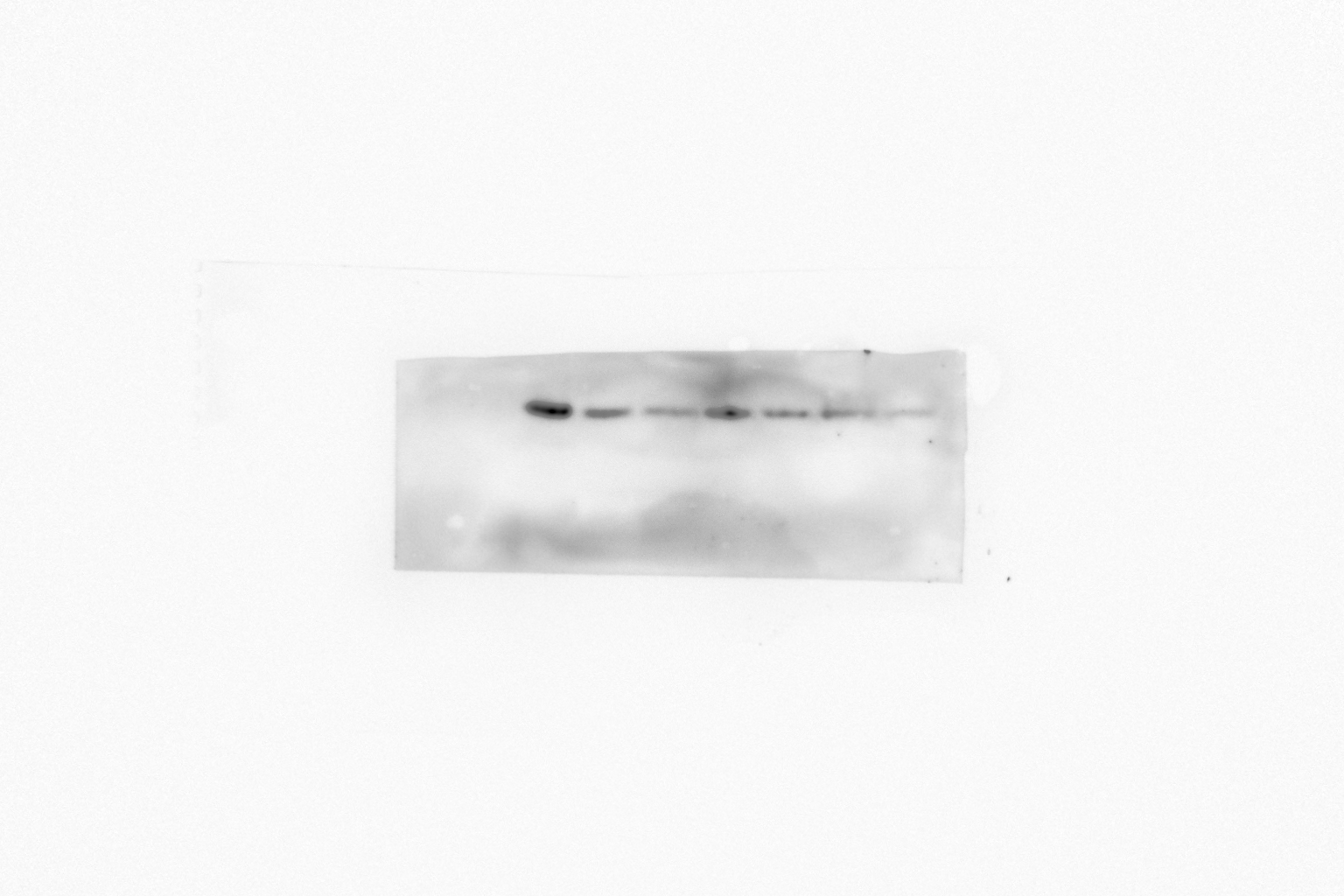

Supplement: Source data 3. [file elife-64960-data3.zip › source data folder 3/Figure 6 source data 2 6B H3K27me3.tif]

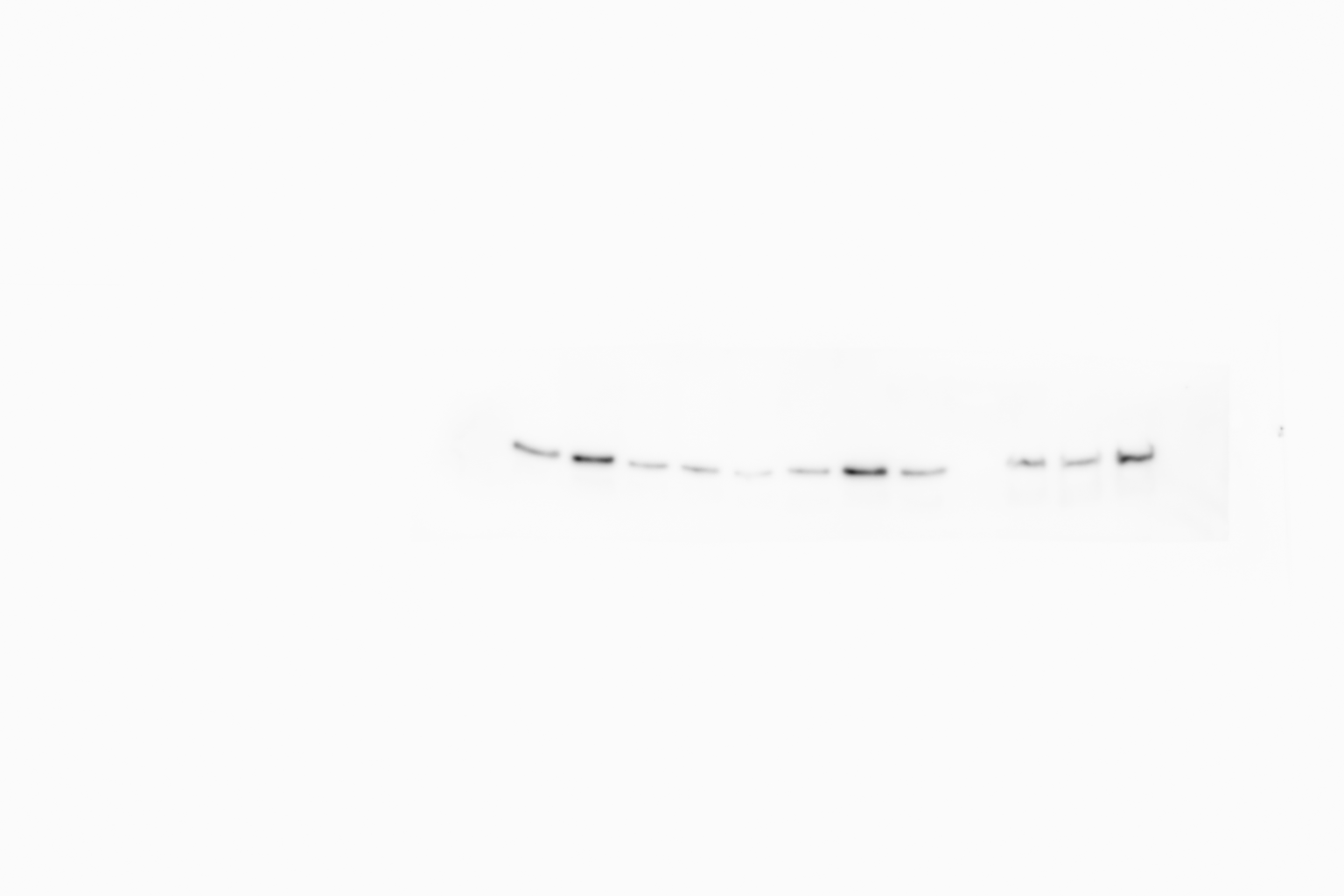

Supplement: Source data 3. [file elife-64960-data3.zip › source data folder 3/Figure 6 figure supplement 1 source data 1 S6C GAPDH.tif]

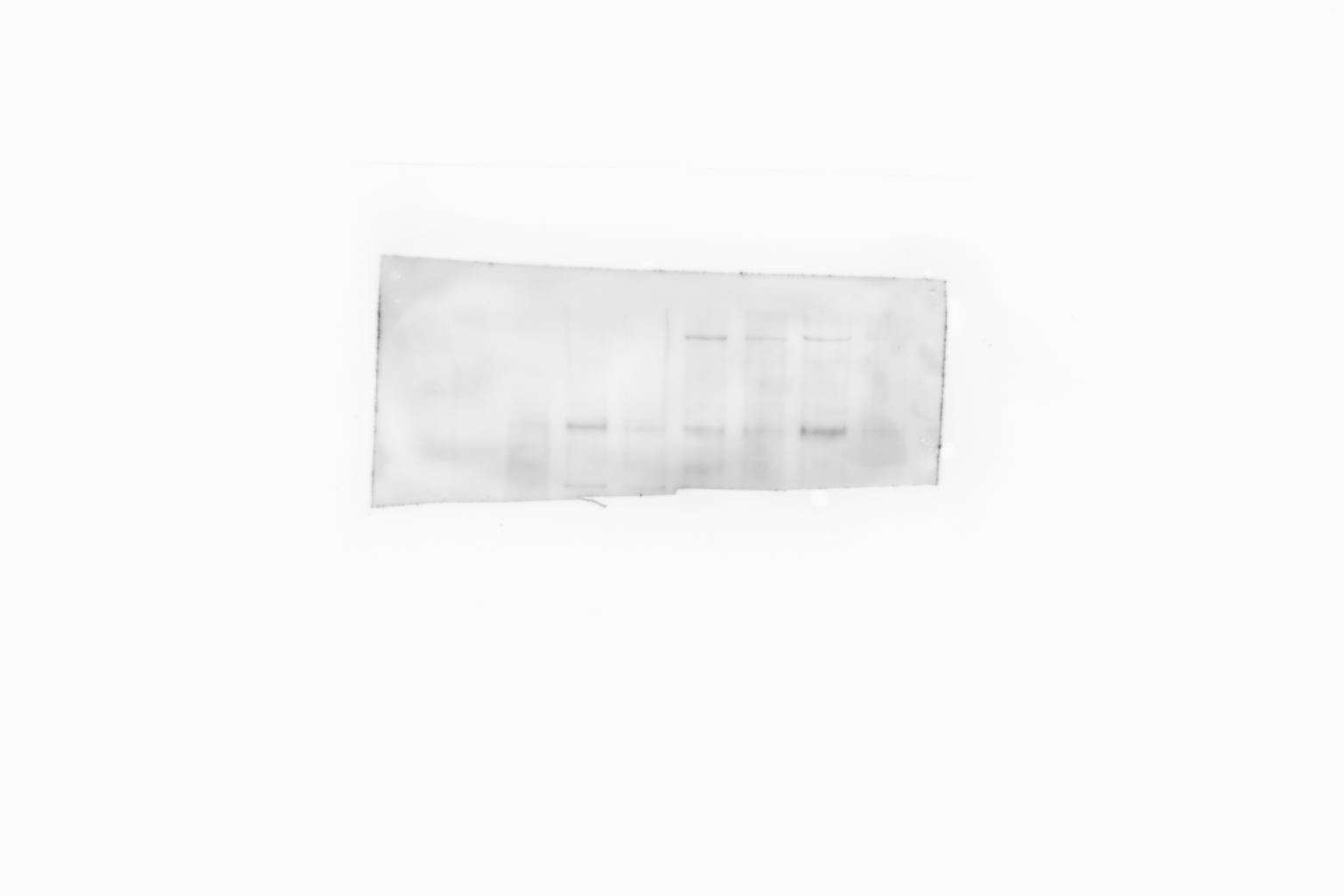

Supplement: Source data 3. [file elife-64960-data3.zip › source data folder 3/Figure 6 source data 1 6B EZH2.tif]

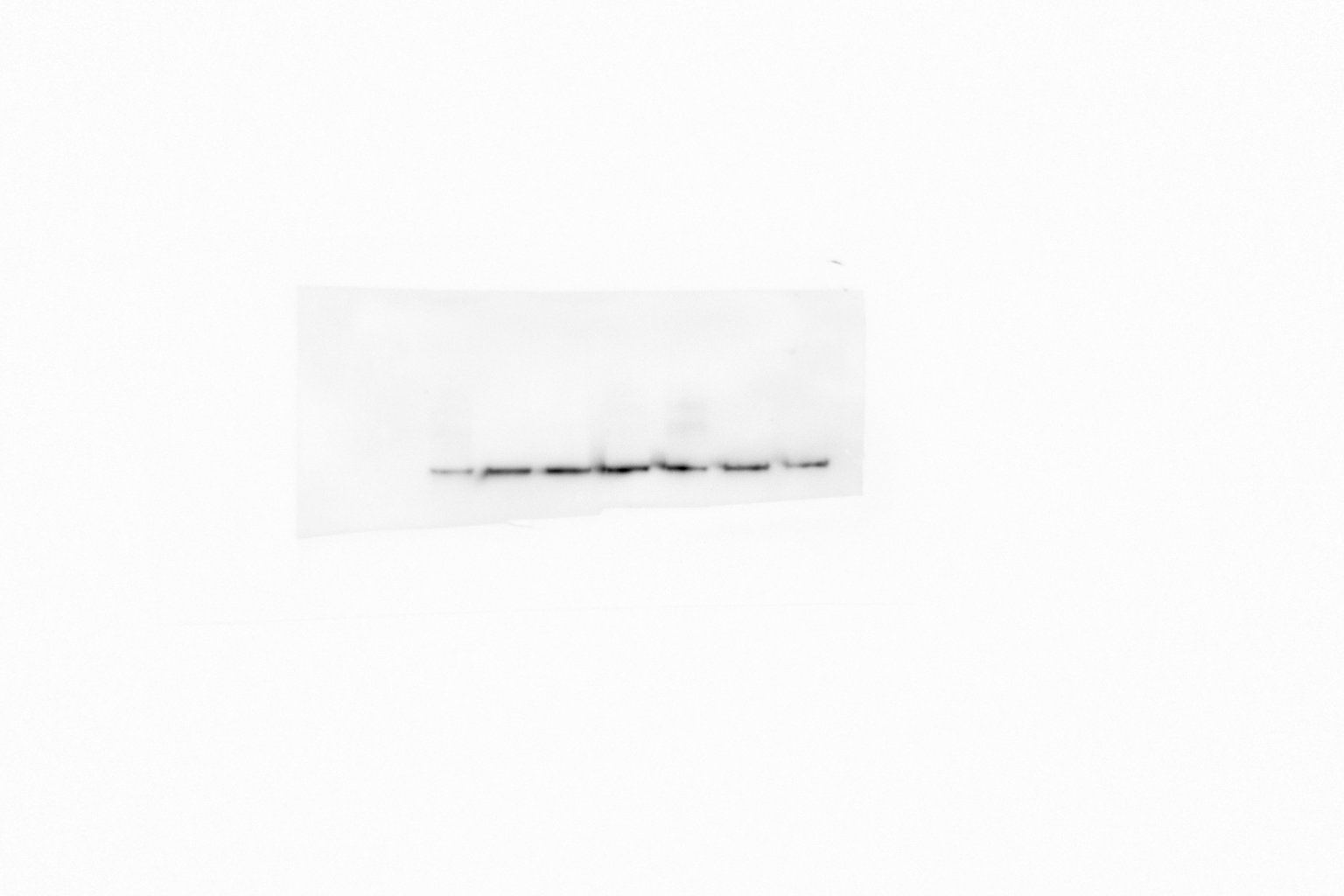

Supplement: Source data 3. [file elife-64960-data3.zip › source data folder 3/Figure 6 source data 3 6B LEDGF.tif]

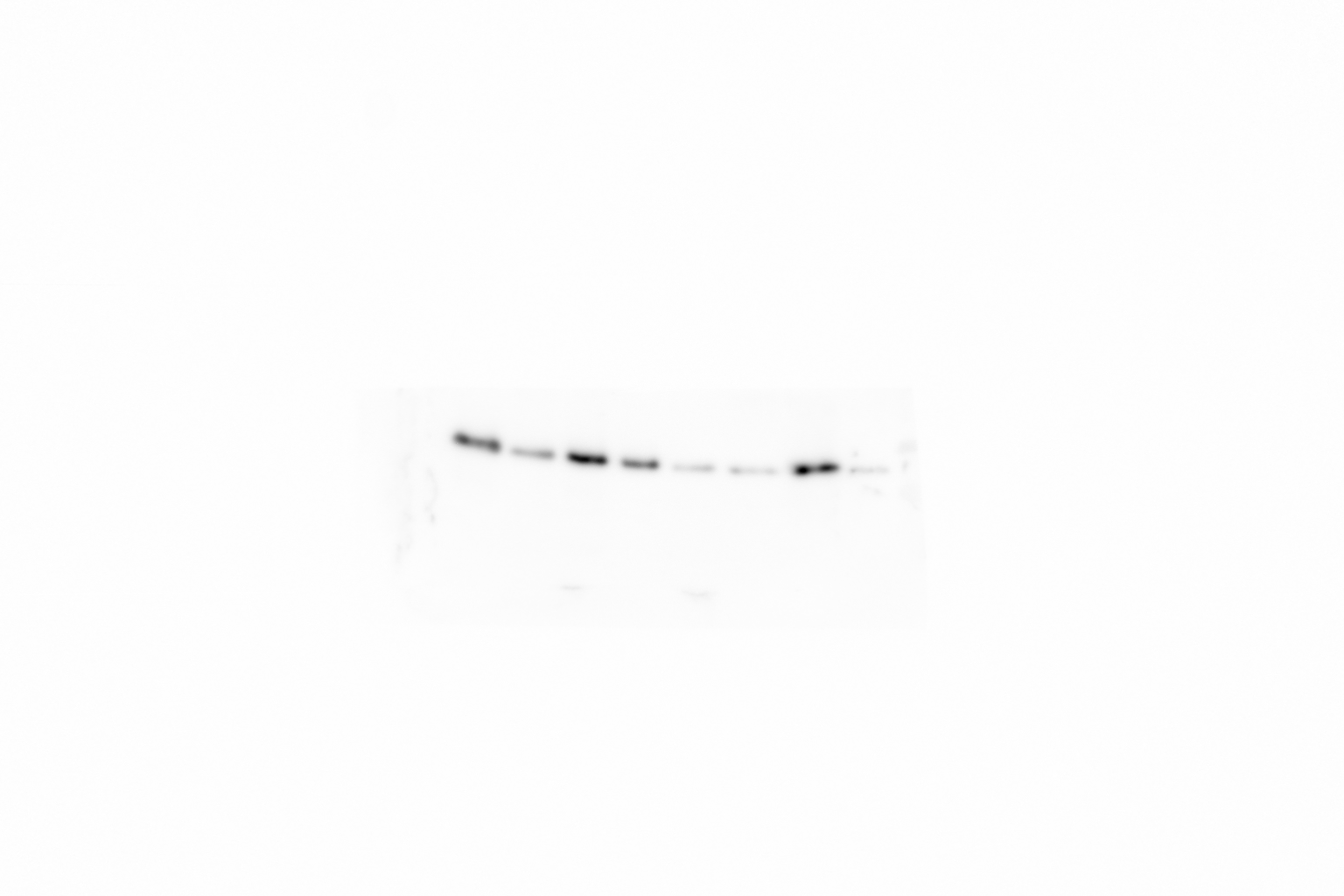

Supplement: Source data 3. [file elife-64960-data3.zip › source data folder 3/Figure 7 figure supplement 1 source data 6 S7C H3K79me2.tif]

Figure 6D

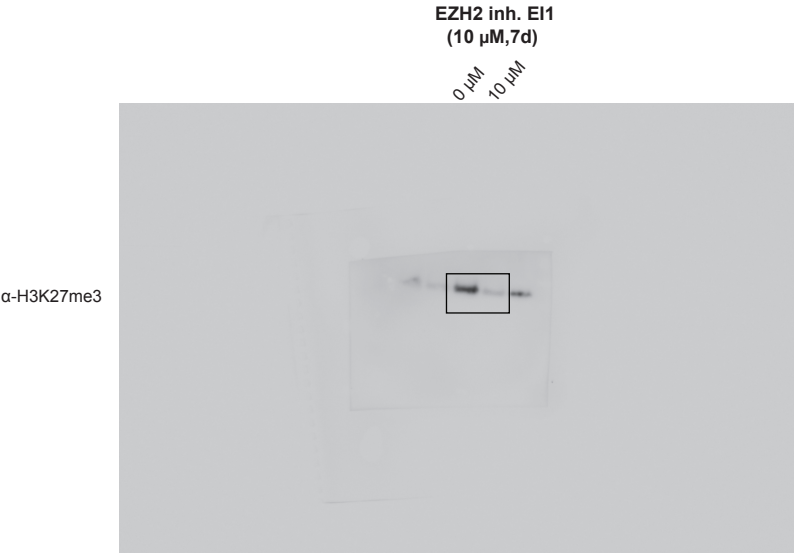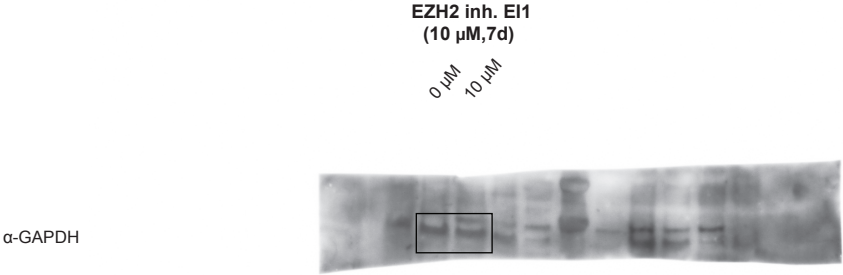

Supplement: Source data 3. [file elife-64960-data3.zip › source data folder 3/Figure 6 source data 7 6D blot labels.pdf]

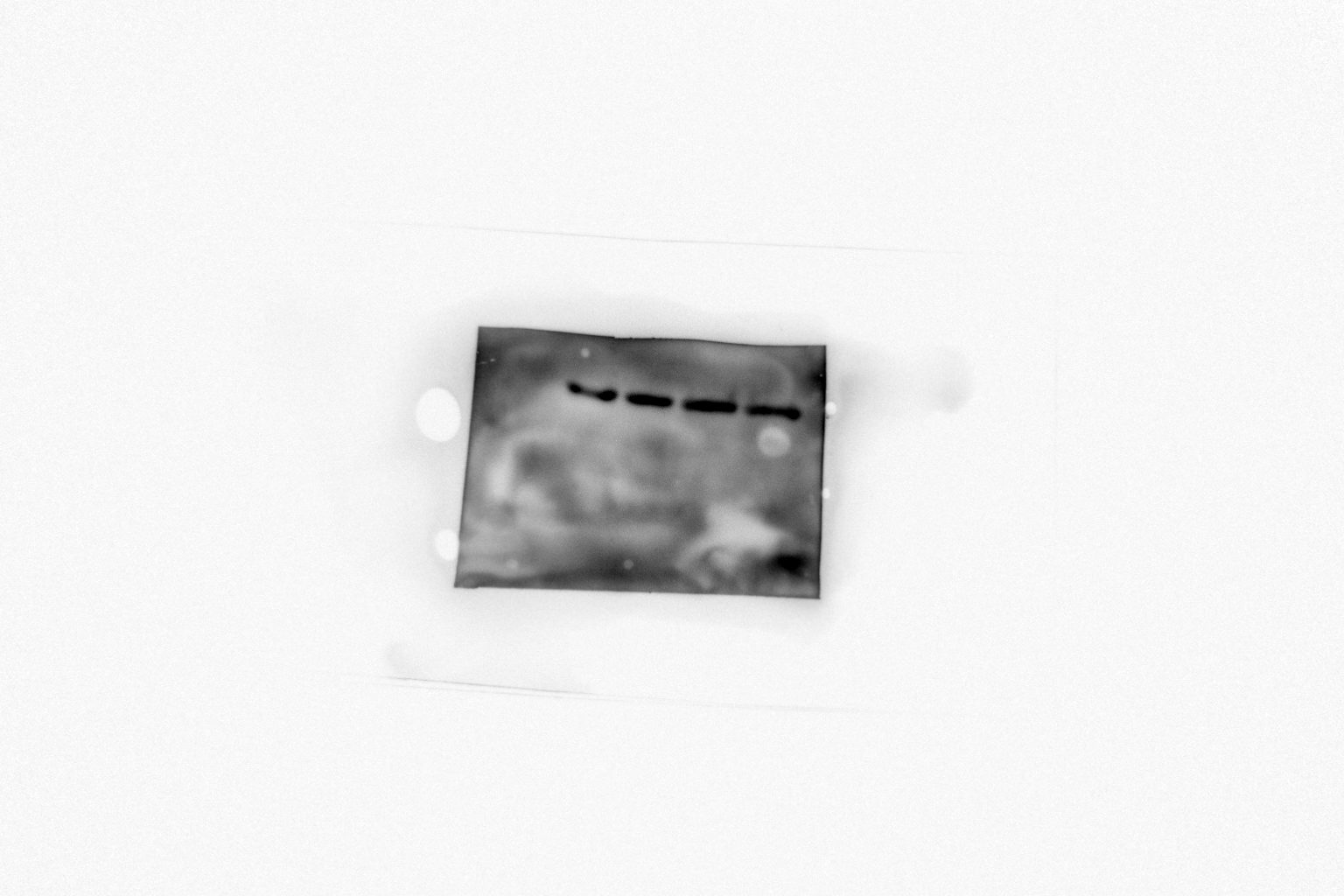

Supplement: Source data 3. [file elife-64960-data3.zip › source data folder 3/Figure 7 figure supplement 1 source data 1 S7A H3K4me3.tif]

Supplementary Figure 7A

MV4;11 (7d)

0 nM  
100 nM pino

- $\alpha$ -H3K4me3

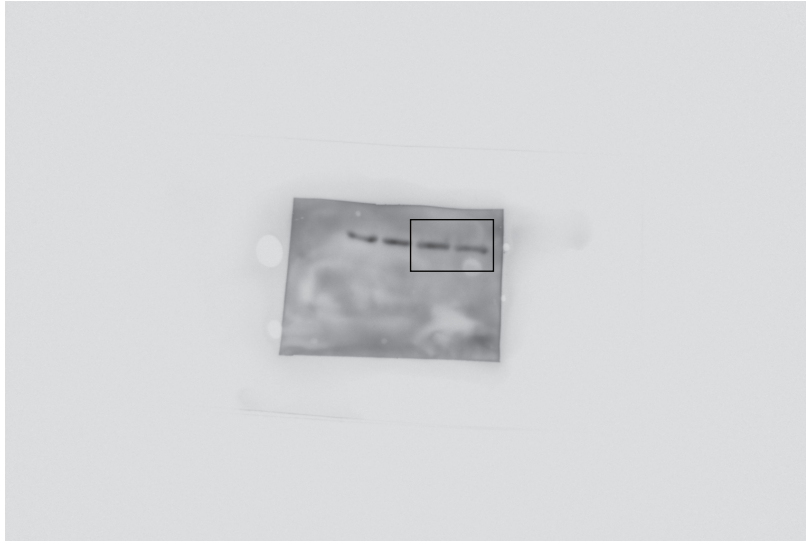

0 nM  
100 nM pino

- $\alpha$ -LEDGF

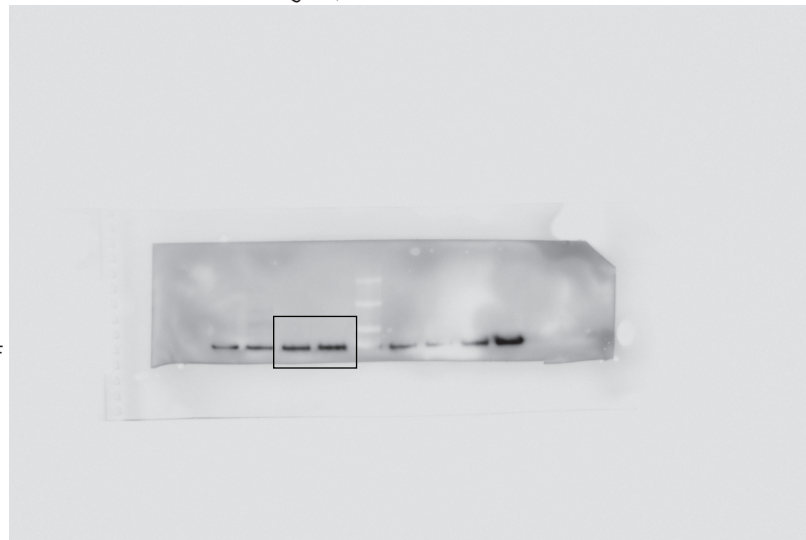

Supplement: Source data 3. [file elife-64960-data3.zip › source data folder 3/Figure 7 figure supplement 1 source data 8 7A blot labels.pdf]

Supplementary Figure 7C

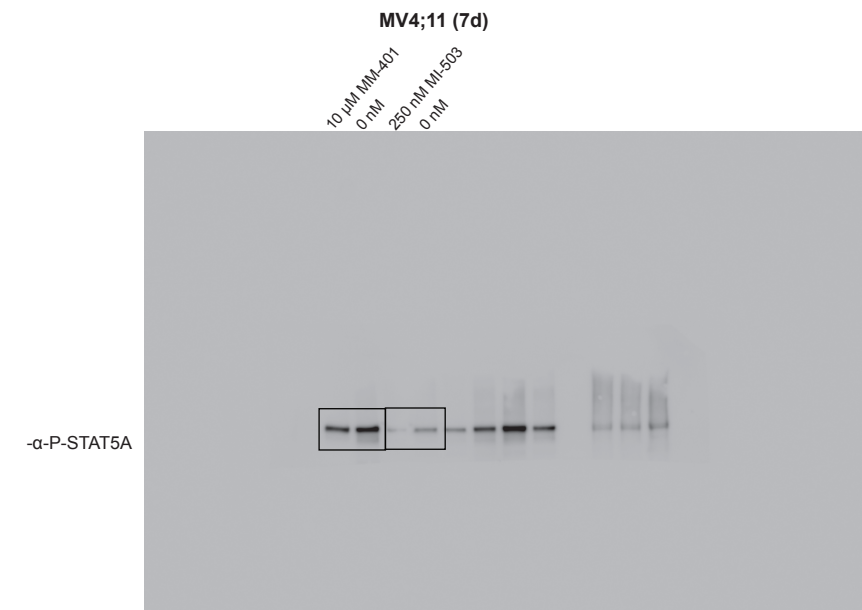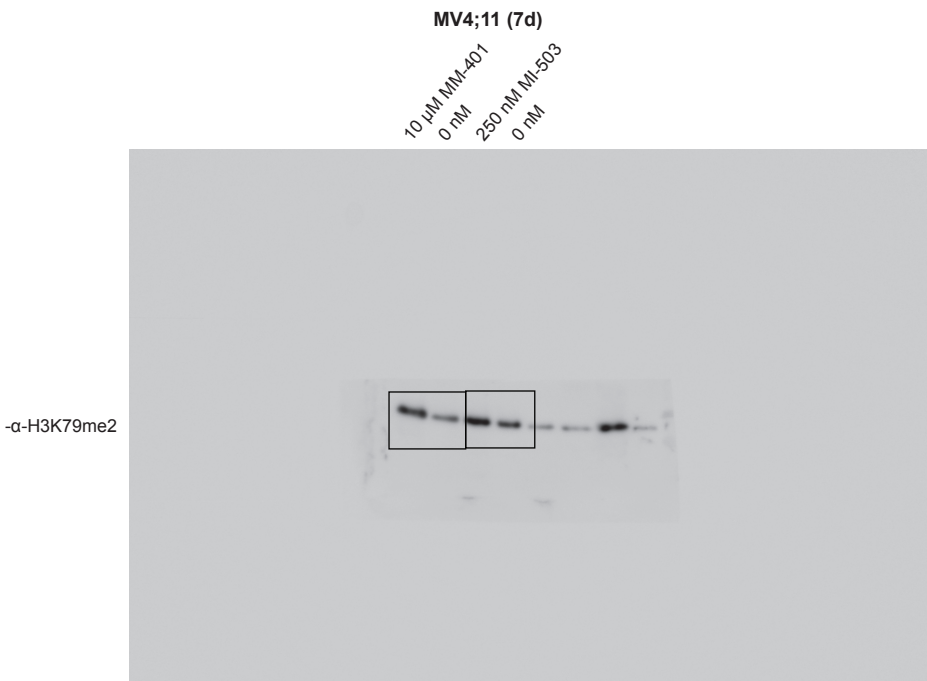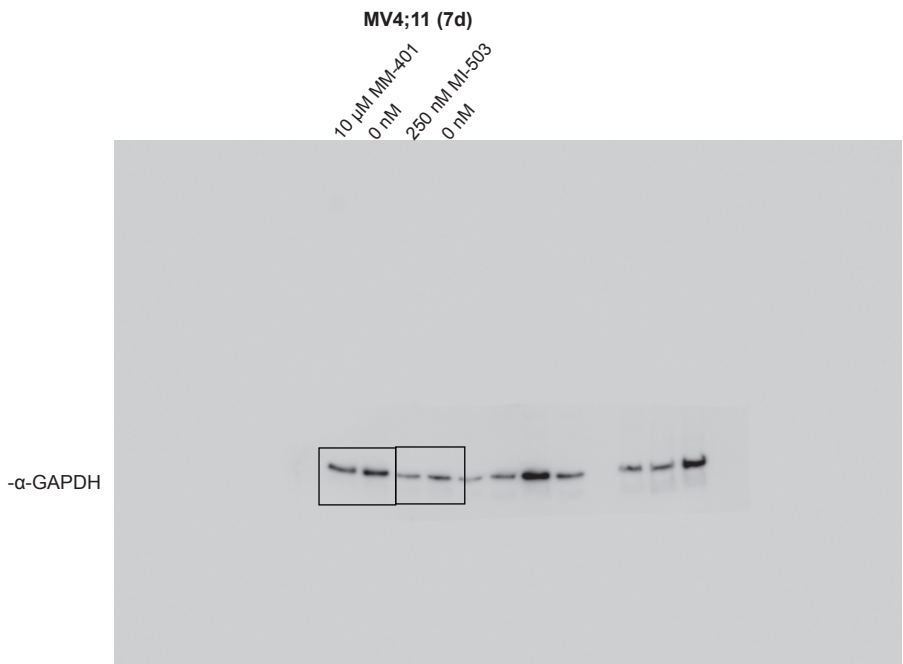

Supplement: Source data 3. [file elife-64960-data3.zip › source data folder 3/Figure 7 figure supplement 1 source data 10 7C blot labels.pdf]

MV4;11 10  $\mu$ M EI1 (7d)

DMSO  
10  $\mu$ M

- $\alpha$ -P-STAT5

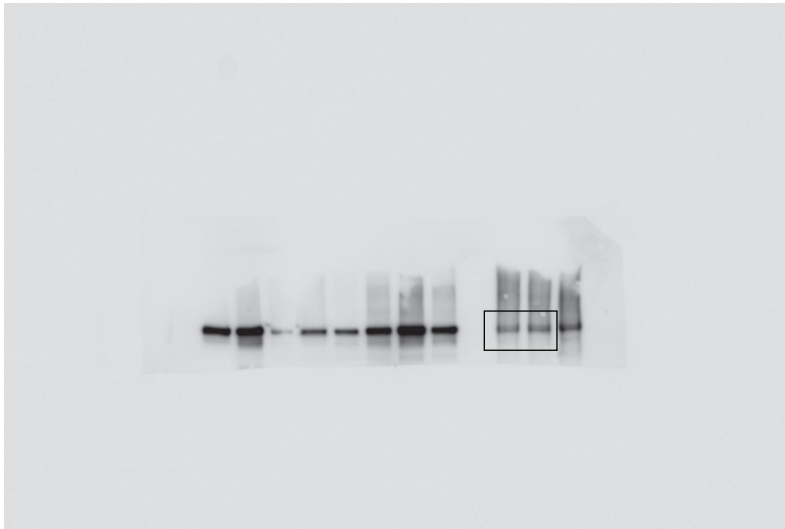

MV4;11 10  $\mu$ M EI1 (7d)

DMSO  
10  $\mu$ M

- $\alpha$ -GAPDH

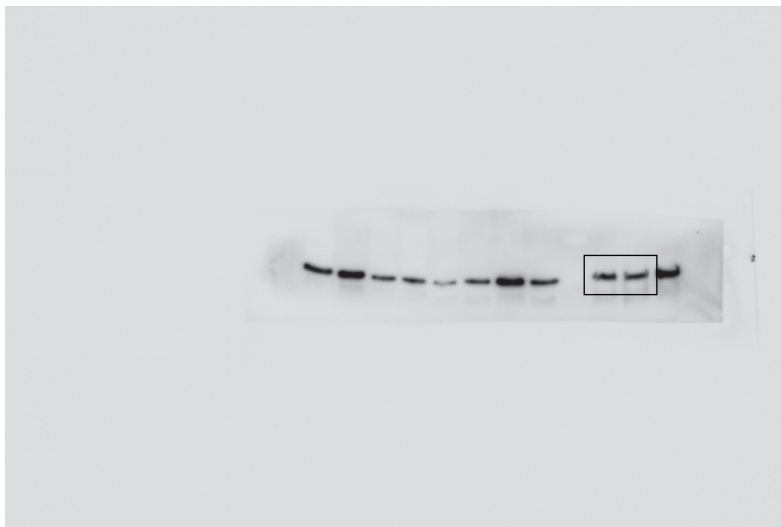

Supplement: Source data 3. [file elife-64960-data3.zip › source data folder 3/Figure 6 figure supplement 1 source data 3 6C blot labels.pdf]

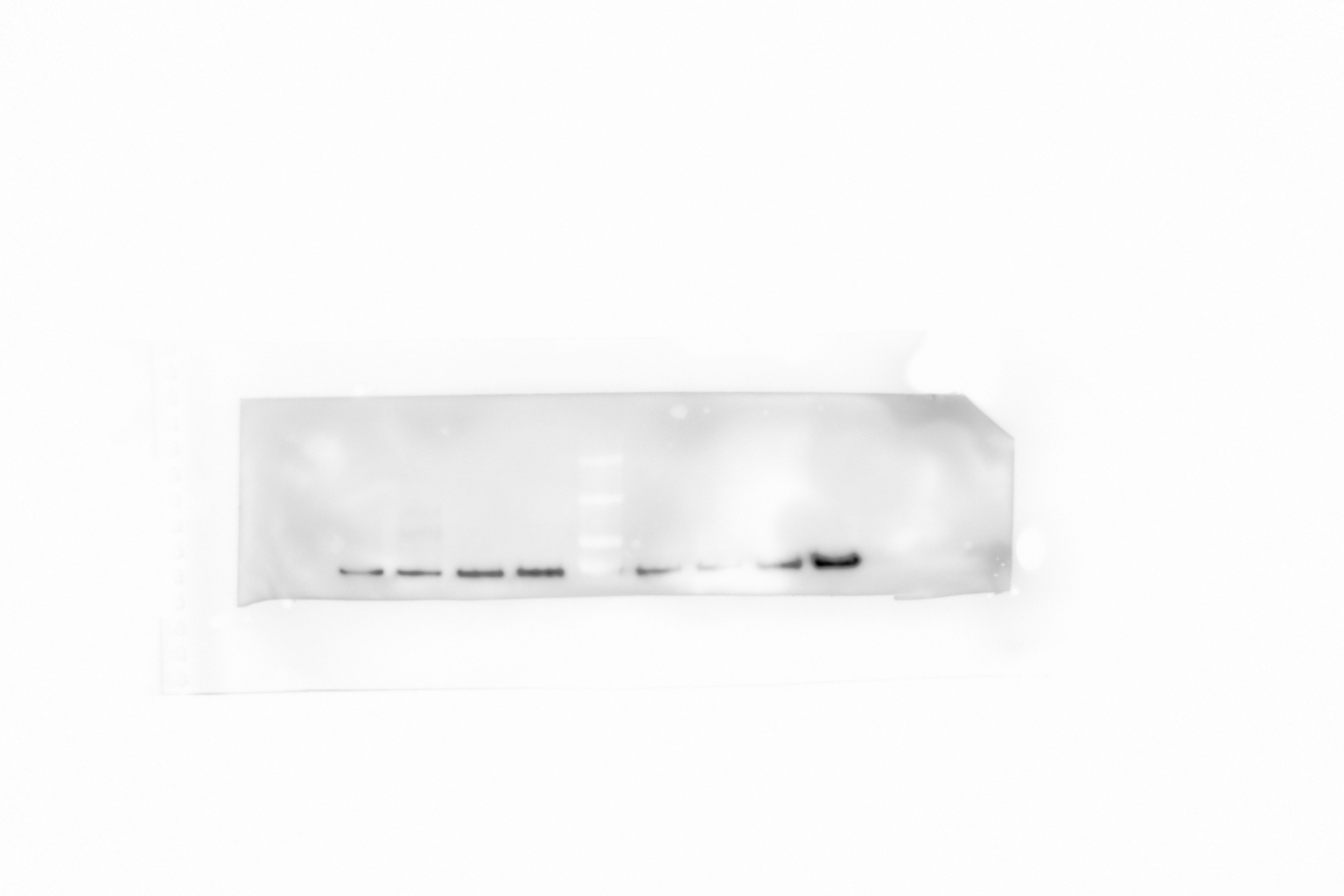

Supplement: Source data 3. [file elife-64960-data3.zip › source data folder 3/Figure 7 figure supplement 1 source data 2 S7A LEDGF.tif]

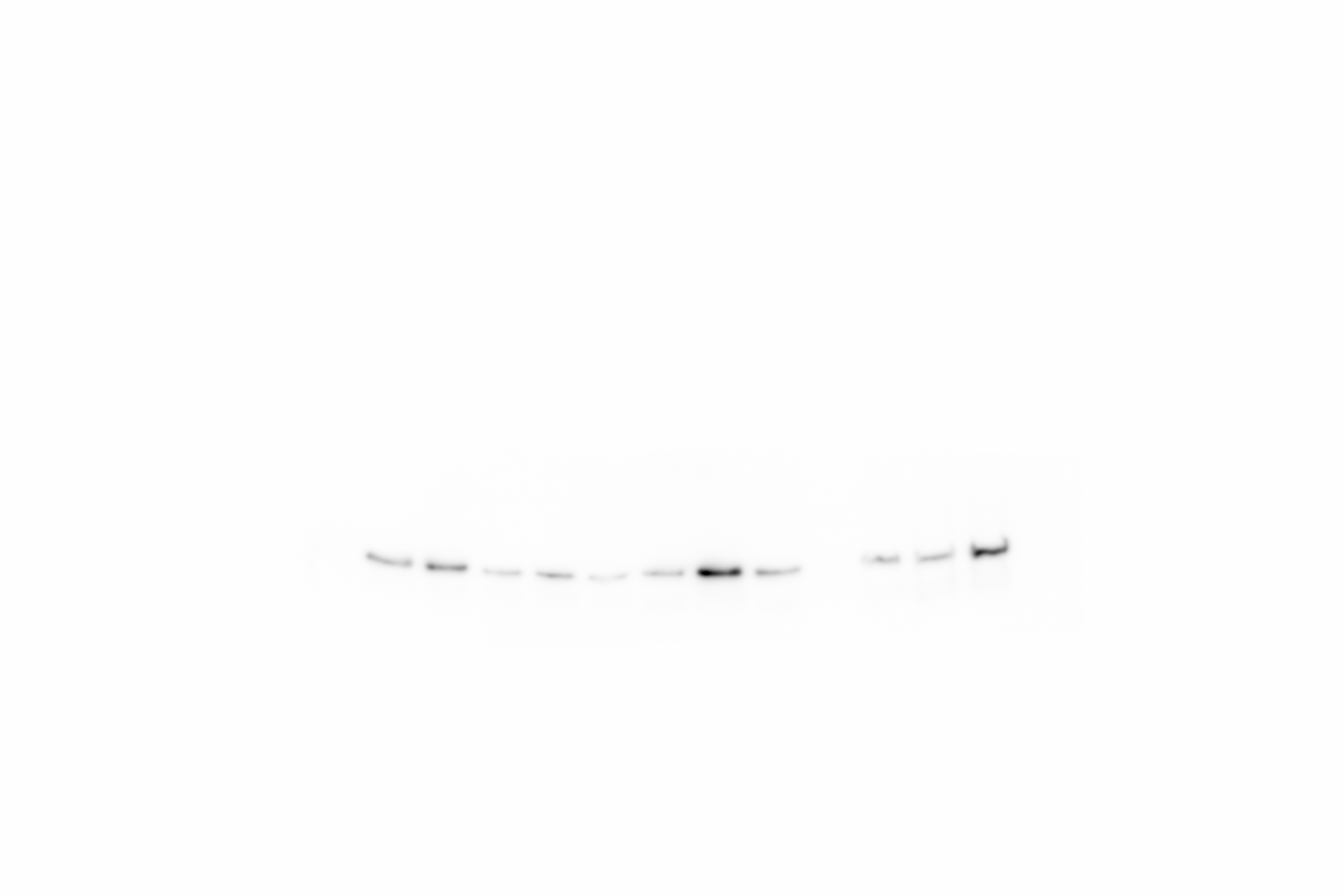

Supplement: Source data 3. [file elife-64960-data3.zip › source data folder 3/Figure 7 figure supplement 1 source data 5 S7C GAPDH.tif]

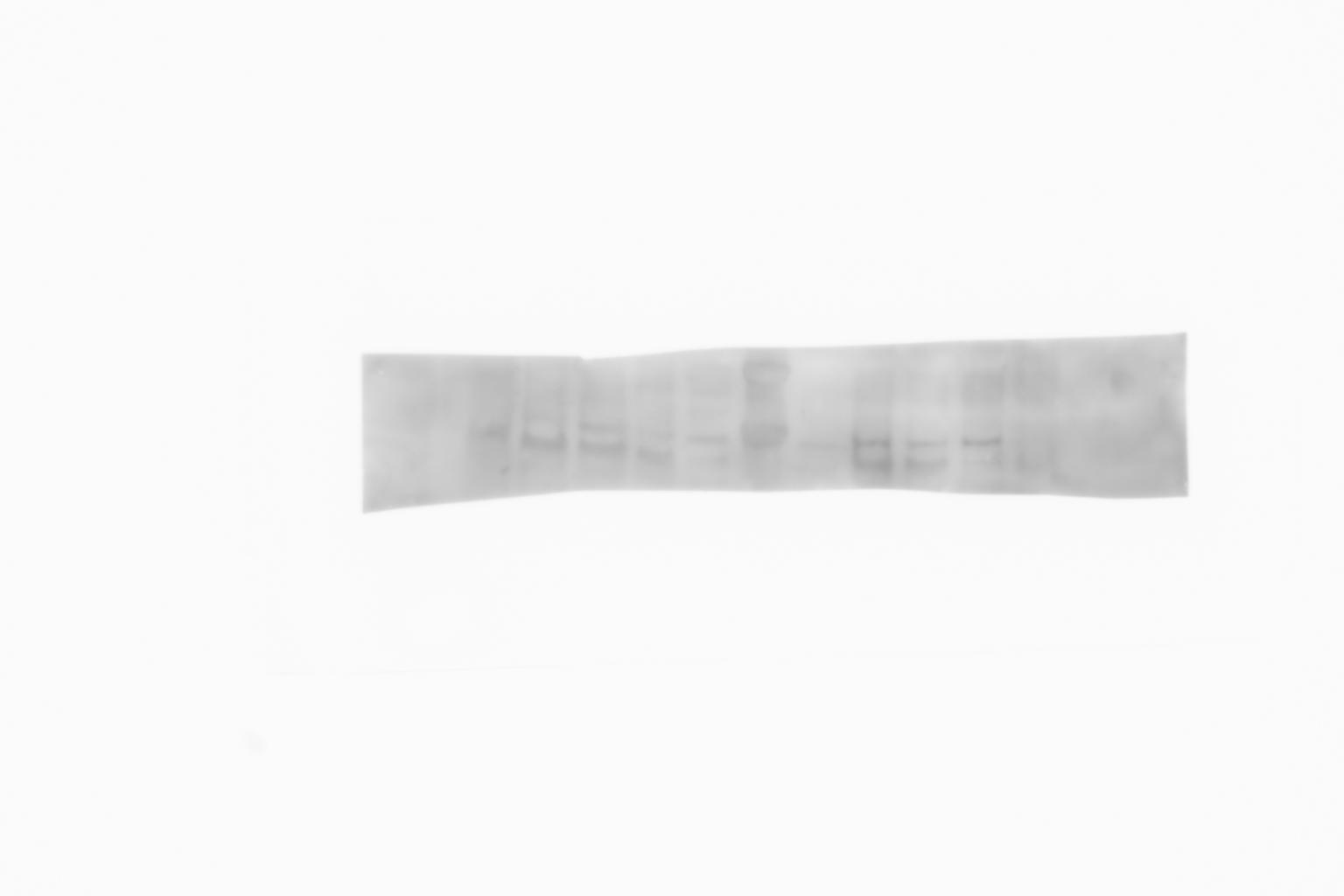

Supplement: Source data 3. [file elife-64960-data3.zip › source data folder 3/Figure 6 source data 4 6C GAPDH.tif]

**Supplementary Figure 7B**

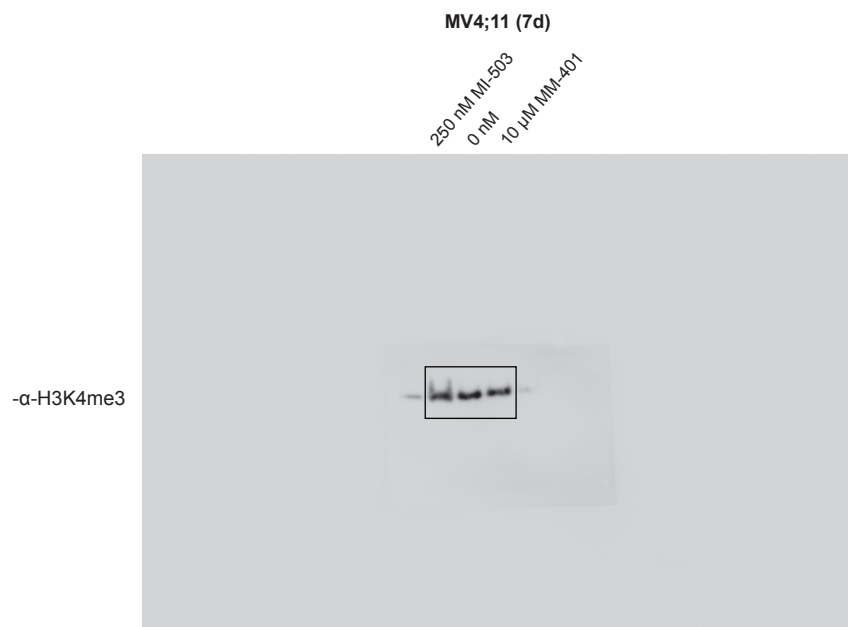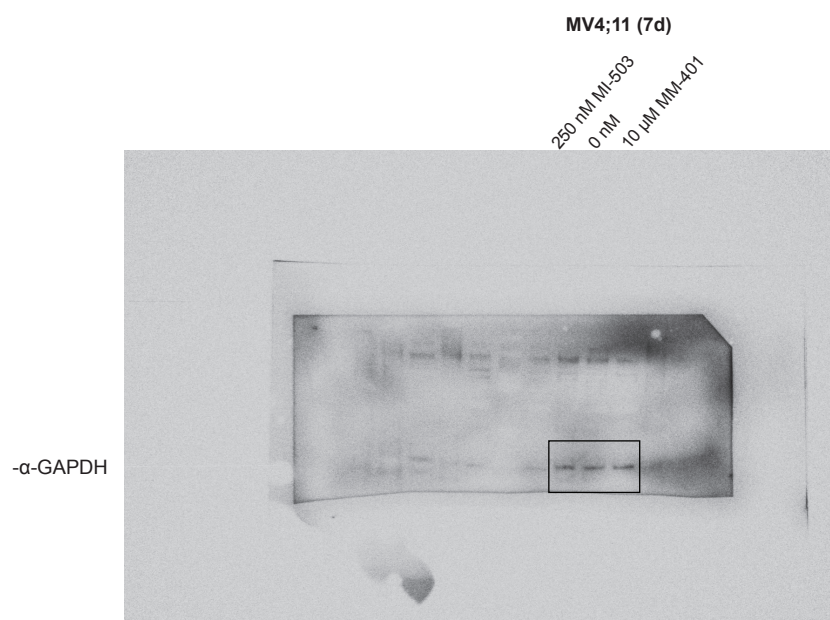

Supplement: Source data 3. [file elife-64960-data3.zip › source data folder 3/Figure 7 figure supplement 1 source data 9 7B blot labels.pdf]

Figure 6B

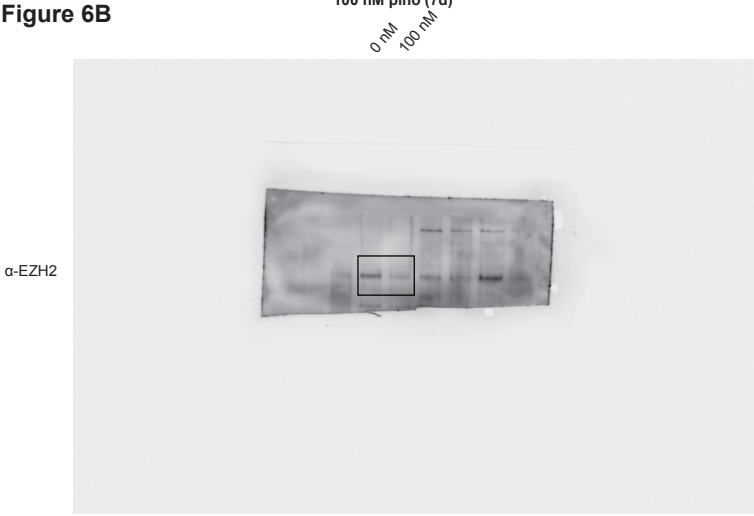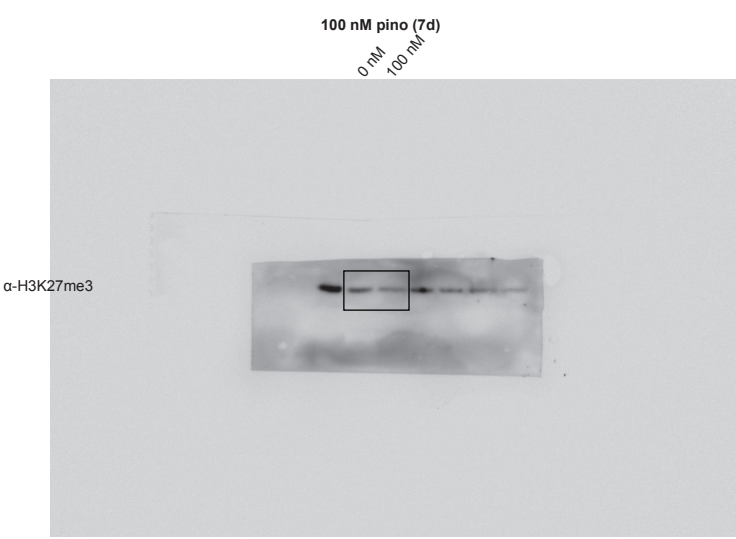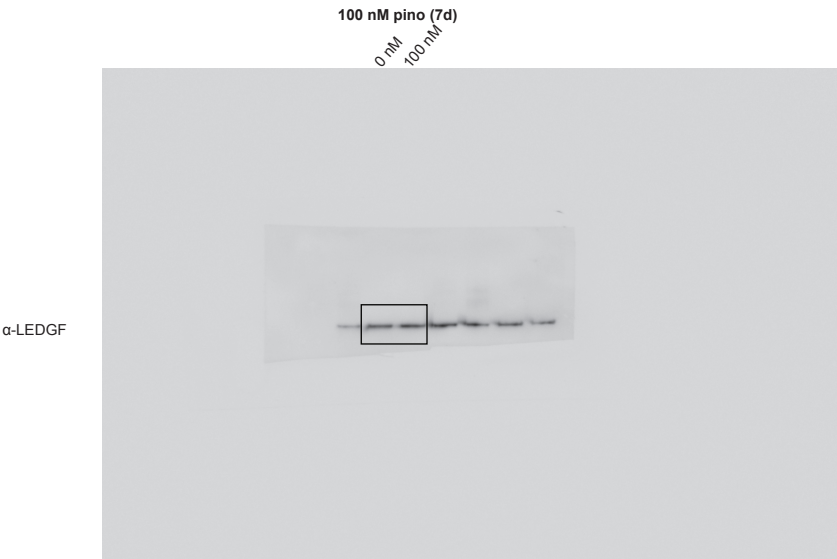

Supplement: Source data 3. [file elife-64960-data3.zip › source data folder 3/Figure 6 source data 6 6B blot labels.pdf]
