# Supplementary material for: Non-canonical H3K79me2-dependent pathways promote the survival of MLL-rearranged leukemia
Source: eLife. 2021 Jul 15;10:e64960. doi: 10.7554/eLife.64960 (PMC8315800; doi:10.7554/eLife.64960)

Figure 4B (left)

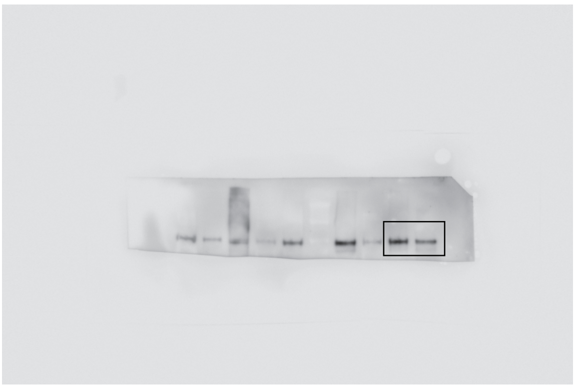

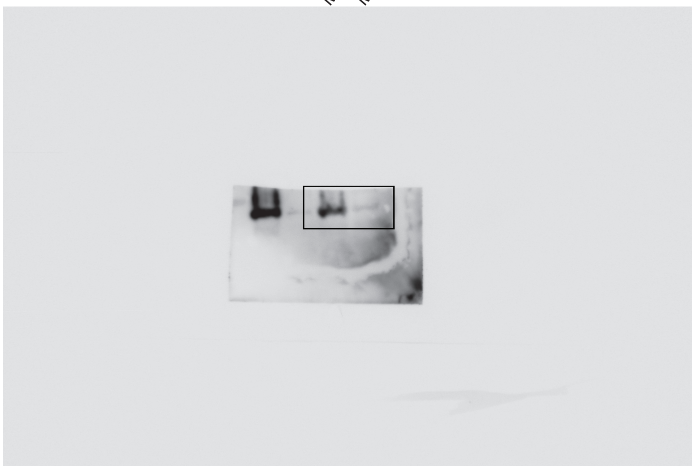

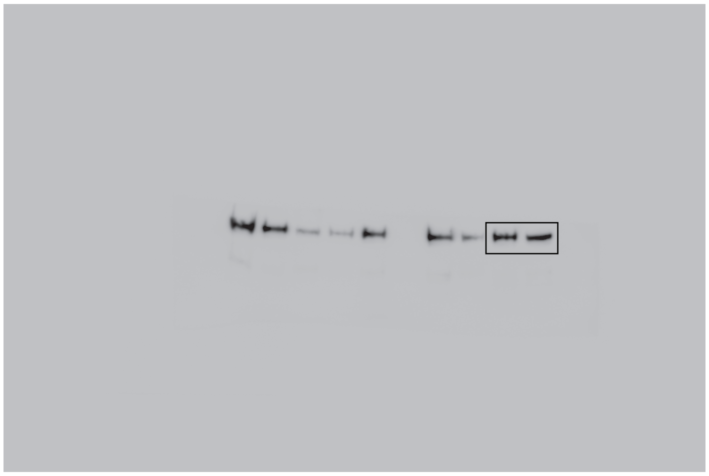

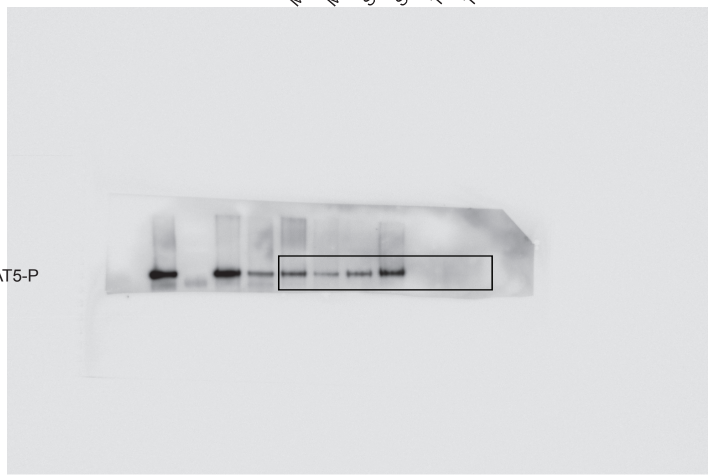

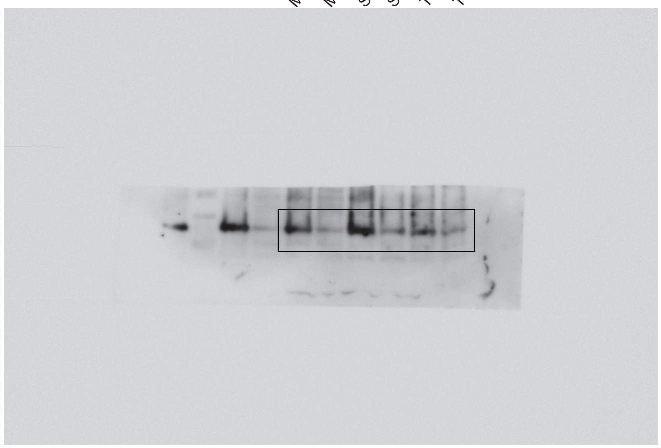

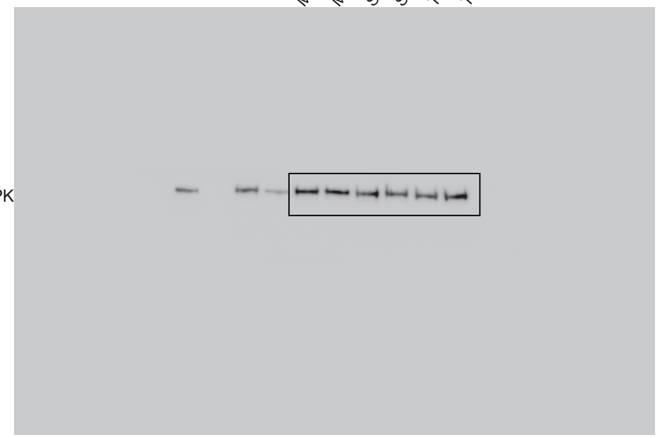

Supplement: Source data 2. [file elife-64960-data2.zip › source data folder 2/Figure 4 source data 16 4B left blot labels.pdf]
