# Supplementary material for: Non-canonical H3K79me2-dependent pathways promote the survival of MLL-rearranged leukemia
Source: eLife. 2021 Jul 15;10:e64960. doi: 10.7554/eLife.64960 (PMC8315800; doi:10.7554/eLife.64960)

Supplementary Figure 5A

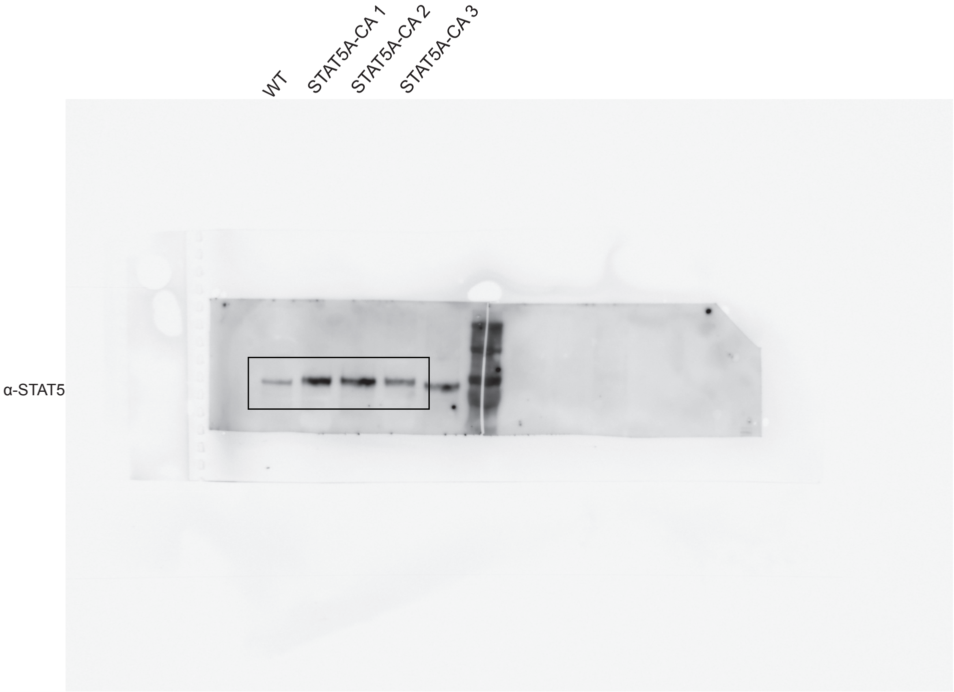

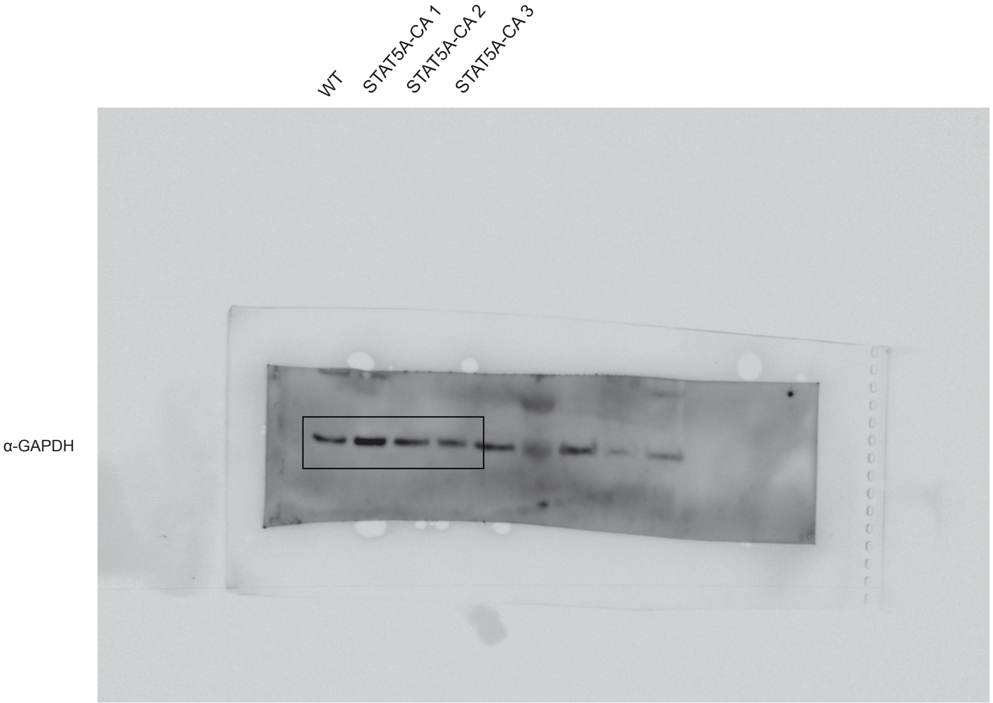

Supplement: Source data 3. [file elife-64960-data3.zip › source data folder 3/Figure 5 figure supplement 1 source data 3 5 blot labels.pdf]
